# SIMON: Open-Source Knowledge Discovery Platform

**DOI:** 10.1016/j.patter.2020.100178

**Published:** 2021-01-08

**Authors:** Adriana Tomic, Ivan Tomic, Levi Waldron, Ludwig Geistlinger, Max Kuhn, Rachel L. Spreng, Lindsay C. Dahora, Kelly E. Seaton, Georgia Tomaras, Jennifer Hill, Niharika A. Duggal, Ross D. Pollock, Norman R. Lazarus, Stephen D.R. Harridge, Janet M. Lord, Purvesh Khatri, Andrew J. Pollard, Mark M. Davis

**Affiliations:** 1 Oxford Vaccine Group, Department of Paediatrics, University of Oxford, Oxford, UK; 2 Institute of Immunity, Transplantation, and Infection, Stanford University School of Medicine, Stanford, CA, USA; 3 Deep Medicine, Nuffield Department of Women's and Reproductive Health, University of Oxford, Oxford, UK; 4 Graduate School of Public Health and Health Policy, City University of New York, New York, NY, USA; 5 Institute for Implementation Science and Population Health, City University of New York, New York, NY, USA; 6 RStudio, PBC, Boston, MA, USA; 7 Duke Human Vaccine Institute, Duke University, Durham, NC, USA; 8 MRC-Versus Arthritis Centre for Musculoskeletal Ageing Research, Institute of Inflammation and Ageing, University of Birmingham Research Labs, Birmingham, UK; 9 Centre for Human and Applied Physiological Sciences, King's College London, UK; 10 NIHR Birmingham Biomedical Research Centre, University Hospital Birmingham NHS Foundation Trust and University of Birmingham, Birmingham, UK; 11 Center for Biomedical Informatics Research, Department of Medicine, Stanford University, Stanford, CA, USA; 12 Department of Microbiology and Immunology, Stanford University School of Medicine, Stanford, CA, USA; 13 Howard Hughes Medical Institute, Stanford University, Stanford, CA, USA

**Keywords:** machine learning, data science, data mining, software, systems biology, computational biology, bioinformatics, artificial intelligence, autoML

## Abstract

Data analysis and knowledge discovery has become more and more important in biology and medicine with the increasing complexity of biological datasets, but the necessarily sophisticated programming skills and in-depth understanding of algorithms needed pose barriers to most biologists and clinicians to perform such research. We have developed a modular open-source software, SIMON, to facilitate the application of 180+ state-of-the-art machine-learning algorithms to high-dimensional biomedical data. With an easy-to-use graphical user interface, standardized pipelines, and automated approach for machine learning and other statistical analysis methods, SIMON helps to identify optimal algorithms and provides a resource that empowers non-technical and technical researchers to identify crucial patterns in biomedical data.

## Highlights

• SIMON is an open-source software for analysis of high-dimensional biomedical data
• SIMON facilitates application of 180+ machine learning algorithms
• Easy-to-use graphical use interface and no programming expertise required
• SIMON empowers biomedical researchers to identify patterns in biomedical data

## The Bigger Picture

Over the past years, technological advances have enabled the generation of large amounts of data at multiple scales. The integration of high-dimensional data is particularly important in biomedical sciences, as they can be used to identify biological mechanisms and predict clinical outcomes well in advance of their occurrence. Because of the lack of powerful analytical tools that can be used by the average biomedical researcher, translation of such knowledge has been extremely slow. We have developed an open-source software, SIMON, to facilitate the application of machine learning to high-dimensional biomedical data. In SIMON, analysis is performed using an intuitive graphical user interface and standardized, automated machine learning approach allowing non-technical researchers to identify patterns and extract knowledge from high-dimensional data and build high-quality predictive models.

## Graphical Abstract

**Figure:**
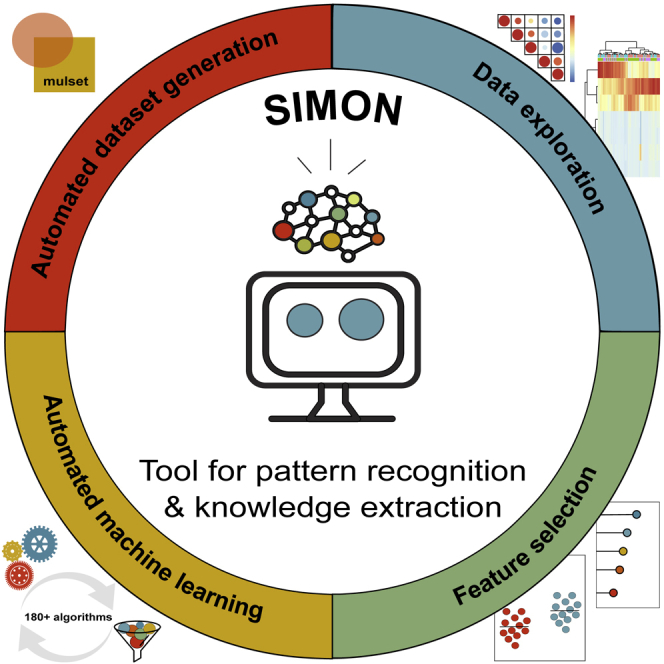

## Teaser

Tomic et al. developed SIMON, an open-source software for application of machine learning algorithms to high-dimensional biomedical data ranging from the transcriptome to flow cytometry to the microbiome. Using a graphical user interface, standardized pipelines for predictive modeling, and automated machine learning, SIMON empowers non-technical biomedical researchers to identify patterns in their data and build high-quality predictive models.

## Introduction

Over the past several years, due to the technological breakthroughs in genome sequencing,^1^ high-dimensional flow cytometry,^2^, ^3^, ^4^ mass cytometry,^5^^,^^6^ and multiparameter microscopy,^7^^,^^8^ the amount and complexity of biological data have become increasingly intractable and it is no longer feasible to extract knowledge without using sophisticated computer algorithms. Therefore, researchers are in need of novel computational approaches that can cope with the complexity and heterogeneity of data in an objective and unbiased way. Machine learning (ML), a subset of artificial intelligence, is a computational approach developed to identify patterns from the data in order to make predictions on new data.^9^ ML has had a profound impact on biological research,^10^, ^11^, ^12^ including genomics,^13^ proteomics,^14^, ^15^, ^16^ cell image analysis,^17^ drug discovery and development,^18^ and cell phenotyping,^6^^,^^19^^,^^20^ which revolutionized our understanding of biological complexity. Recently, using systems-level analysis of genetic, transcriptional, and proteomic signatures to predict patients' response to vaccines,^21^^,^^22^ therapies, and disease progression,^23^, ^24^, ^25^, ^26^, ^27^ ML has become the primary computational approach used in “precision medicine.”^28^

The biggest challenge is the proper application of ML methods and the translation of the results into meaningful insights. The analysis of massive datasets and extraction of knowledge using ML require knowledge of many different computational libraries for data pre-processing and cleaning, data partitioning, model building and tuning, evaluation of the performance of the model, and minimizing overfitting.^11^ Tools to achieve these tasks have been mainly developed in either R (https://www.r-project.org/)^29^^,^^30^ or Python (www.python.org/),^31^ which have today become leading statistical programming languages in data science. Because R and Python are free and open source, they have been quickly adopted by a large community of programmers who are building new libraries and improving existing ones. As of May 2020, there are 15,658 R packages available in the CRAN package repository (https://cran.r-project.org/). Many of the packages offer different modeling functions and have different syntaxes for model training, predictions, and determination of variable importance. Due to the lack of a unified method for proper application of ML processes, even experienced bioinformaticians struggle with these time-consuming ML tasks. To provide a uniform interface and standardize the process of building predictive models, ML libraries were developed, for example, mlr3^32^ (https://mlr3.mlr-org.com), classification and regression training (caret)^30^^,^^33^ (https://rdrr.io/cran/caret), scikit-learn^34^ (https://scikit-learn.org), mlPy^35^ (https://mlpy.fbk.eu), and SciPy (https://www.scipy.org/), including also ones for deep learning, such as TensorFlow (https://www.tensorflow.org/), PyTorch (https://pytorch.org/), and Keras (https://keras.io/). Since those libraries do not have a graphical user interface, usage requires extensive programming experience and general knowledge of R or Python, making them inaccessible for many life science researchers. Therefore, there is an increased effort to harmonize those libraries and develop a software that will facilitate application of ML in life sciences.

The software should provide a standardized ML method for data pre-processing, data partitioning, building predictive models, evaluation of model performance, and selection of features. Moreover, such software should be adapted for biological datasets that have a high percentage of missing values,^36^ have unbalanced participant distributions (i.e., a high number of infected subjects, but only a relatively small number of healthy controls),^37^ or suffer from a “curse of dimensionality,” i.e., poor predictive power, as can be observed when the number of features is much greater than the number of samples.^38^ In addition, beyond the ML process, the software should support exploratory analysis and visualization of the results using a user-friendly graphical interface. The fast-paced technological development has dramatically increased the size of biological datasets and the computational power needed for analysis. Therefore, open-source web-based software supporting cloud processing architecture is essential.

## Results

To address these challenges, we developed SIMON (Sequential Iterative Modeling “Over Night”), a free and open-source software for application of ML in life sciences that facilitates production of high-performing ML models and allows researchers to focus on the knowledge discovery process. SIMON provides a user-friendly, uniform interface for building and evaluating predictive models using a variety of ML algorithms. Currently, there are 182 different ML algorithms available (Table S1). The entire ML process, which is based on the caret^33^ library, from model building and evaluation to feature selection, is fully automated, as described.^39^ This allows advanced ML users to focus on other important aspects necessary to build highly accurate models, such as data pre-processing, feature engineering, and model deployment. It also makes the entire ML process more accessible to domain-knowledge experts who formulate the research hypothesis and collect the data, but lack programming ML skills. In addition, to prevent optimistic accuracy estimates and to optimize the model for generalization to unseen data, SIMON introduces a unified process for model training, hyperparameter tuning, and model evaluation by generation of training, validation, and test sets. A training set is used for building models, which are evaluated using 10-fold cross-validation; a validation set is used for hyperparameter tuning, and finally, models are evaluated in an unbiased way using a test set, also known as a holdout set, that has never been used for training. Models can be downloaded as Rdata formats, which is crucial for usability and reproducibility. In addition to the standardized ML process, the initial install version offers a set of core components specifically suited to the analysis of biomedical data, such as a multiset intersection function for integration of data with many missing values (https://cran.r-project.org/web/packages/mulset/index.html), a method for identifying differentially expressed genes using significance analysis in microarrays,^40^ a graphical representation of the clustering analysis important for detection of batch effects, a graphical display of the correlation analysis, and graphical visualizations of the ML results that can be downloaded as publication-ready figures in scalable vector graphics format. Finally, SIMON is available in two versions as a single mode and a server version. The single mode is developed as a SIMON Docker container (https://www.docker.com/), ensuring code reproducibility and solving installation compatibility issues across major operating systems (Windows, MacOS, and Linux). In both versions parallel computing is supported, which is essential for more efficient ML analysis by distributing the workload across several processors. To promote collaboration and data sharing and support distributed cloud processing, SIMON is also available as a server version. The server version can be installed on a private or public Linux cloud service. Distributed cloud processing (multiNode) is implemented utilizing OpenStack, a free and open-source cloud computing platform (https://www.openstack.org/). The advantage of the server version is that it has multiNode capability, which allows users to distribute workload on multiple computers simultaneously to optimize SIMON performance. The multiNode process can be used to horizontally scale analysis to large infrastructures, such as high-performance computing clusters to meet the computational needs and accommodate parallel processing of large amounts of data. In addition, in the server version, users can configure data storage either on a local server or in a cloud-using service that is interoperable with the Amazon Web Services S3 application programming interface.^41^ SIMON has also been translated into multiple languages by a collaborative open-source effort. SIMON source code is regularly updated, and both source code and compiled software are available from the project's website at http://www.genular.org/.

**Figure 1:**
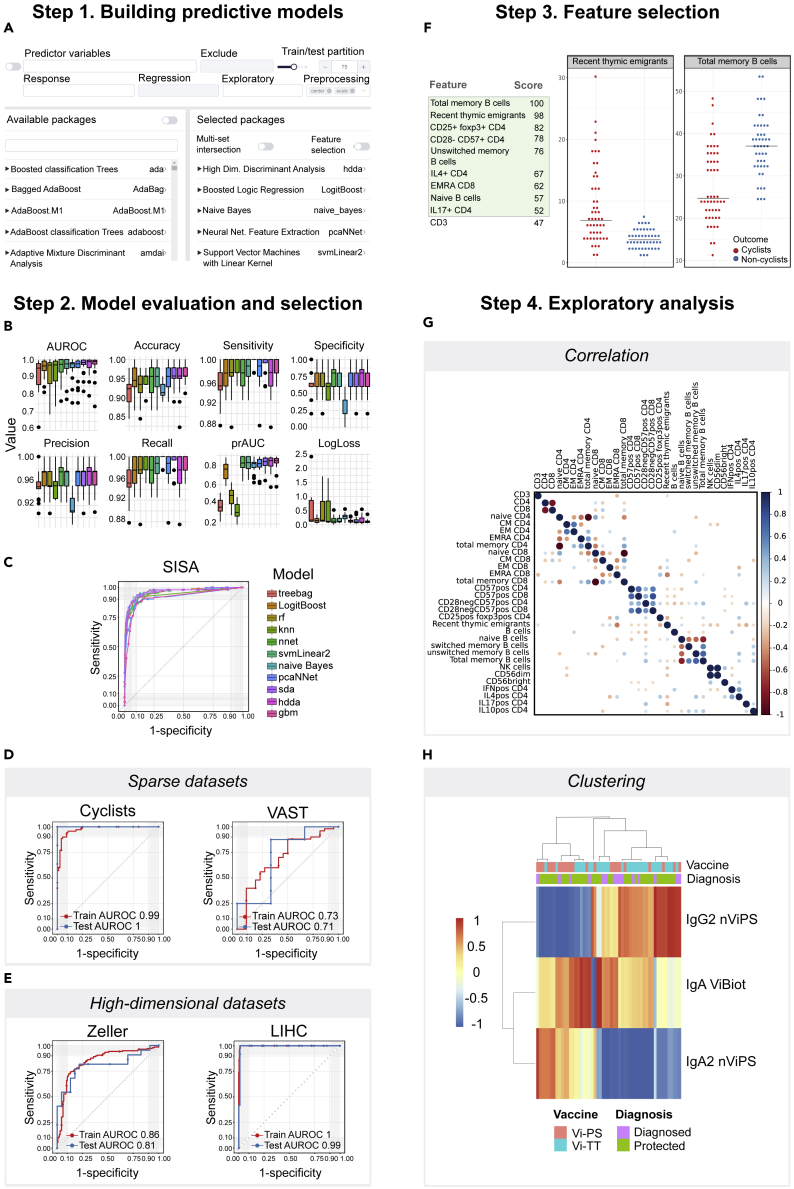
SIMON Machine Learning Workflow Step 1. Building predictive models. (A) Screenshot of the SIMON graphical user interface demonstrating input selection for machine learning analysis, such as predictors and response (outcome) variables, additional exploration classes, training/test split, pre-processing functions, and desired machine learning algorithms. Step 2. Model evaluation and selection. Comparison of (B) box plots of performance measurements calculated for 11 predictive models and (C) receiver operating characteristic (ROC) curves built on the SISA dataset. Each boxplot shows the distribution of data as minimum (Q1−1.5×IQR), first quartile (Q1), median (Q2), third quartile (Q3), and maximum (Q3+1.5×IQR). Data outside of minimum and maximum values (outliers) are shown as circles. IQR, interquartile range.Comparison of ROC curves calculated from the training (average value calculated using 10-fold cross-validation repeated three times) and test sets on (D) datasets with missing values (Cyclists and VAST) and (E) high-dimensional datasets (Zeller and LIHC). Step 3. Feature selection. (F) The variable importance score table for each feature and graphical visualization of the selected features from the Cyclists dataset. Step 4. Exploratory analysis. (G) Correlation analysis on the Cyclists dataset. (H) Clustering analysis on the VAST dataset.

We demonstrate the accuracy, ease of use, and power of SIMON on five different biomedical datasets and build predictive models for arboviral infection severity (SISA),^42^ the identification of the cellular immune signature associated with a high-level of physical activity (Cyclists),^43^ the determination of the humoral responses that mediate protection against *Salmonella* Typhi infection (VAST),^44^ early stage detection of colorectal cancer from microbiome data (Zeller),^45^^,^^46^ and the detection of liver hepatocellular carcinoma cells (LIHC)^47^ (Figure 1B–1E; Supplemental Information, Videos S1 and S6). To build models using the SISA dataset containing clinical parameters (described in the Experimental Procedures and available as Table S2), 11 ML algorithms were used, 5 from the original publication^42^ (treebag, k nearest neighbors, random forest, stochastic generalized boosting model, and neural network) and, in addition, “sda,” shrinkage discriminant analysis; “hdda,” high-dimensional discriminant analysis; “svmLinear2,” support vector machine with linear kernel; “pcaNNet,” neural networks with feature extraction; “LogitBoost,” boosted logistic regression, and naive Bayes. Due to the unified ML process for training, tuning, and evaluating predictive models, users can test a variety of ML algorithms in SIMON. Since the same training and test sets are used by different algorithms, resulting models can be compared and the best-performing models can be selected. After manually setting initial parameters for data partitioning, predictor and outcome variables, exploratory classes, pre-processing, and selecting ML algorithms (Figure 1A), SIMON automatically performs all necessary ML analysis steps to build, tune, and evaluate predictive models. The process of building all 11 models on the SISA dataset in SIMON finished in 59 s on a standard laptop (Intel Core i7 Processor 7700HQ and 16 GB of RAM). In SIMON, users can evaluate model performance using standard performance measurements such as accuracy, sensitivity, specificity, precision, recall, area under the receiver operating characteristic curve (AUROC), precision-recall area under curve (prAUC), and logarithmic loss (LogLoss) on training and holdout test sets (Figure 1B; Videos S2 and S3). The shrinkage discriminant analysis model (sda) had the highest AUROC of 0.97 on the training set and also performed well on the holdout test set (test AUROC 0.96) (Figure 1C, Table S3, the model is available as the Data S1).

Video S1. Introduction to SIMON

Video S2. Exploration of Models in SIMON

Video S3. Evaluation and Comparison of Models in SIMON

Video S6. Running SIMON Analysis

To demonstrate SIMON's capabilities for analyzing biomedical datasets with missing data, we applied SIMON to (1) the Cyclists dataset studying the impact of physical activity on the immune system in adulthood based on immunophenotyping using flow cytometry^43^ (Table S4) and (2) the VAST dataset containing serological analysis of the antibody responses collected from a clinical trial that was undertaken to evaluate typhoid vaccine efficacy^48^ (Table S5). Description of both datasets is available in the Experimental Procedures. The percentage of missing values was 8% in the Cyclists dataset and 21% in the VAST dataset, due to either the exclusion of samples not passing quality control criteria or the lack of sample volume to repeat experiments and obtain reportable data. To build models using the datasets with missing values, we used the multiset intersection (“mulset”) function^39^ to identify shared features between donors and generate resamples (Supplemental Information). Because the mulset function generates multiple resamples from the initial dataset based on shared features, it is useful for removal of missing values and can be used for integration of data collected from different assays and across clinical studies.^39^ For the Cyclists dataset, the mulset function generated 146 resamples. The models were built for each of the 146 resamples using five ML algorithms (naive Bayes, svmLinear2, pcaNNet, logistic regression, and hdda) to identify immune cell subsets enriched in the cohort of master cyclists. The analysis finished in 41 min and 24 s. The model with the highest performance measures was built with naive Bayes on the resample with 96 donors that shared 31 features (train AUROC 0.99 and test AUROC 1) (Figure 1D, Table S6, and Data S2). The mulset function generated 206 resamples from the initial VAST dataset with varying number of donors and features. Resamples with fewer than 10 donors in the test set were removed prior to the ML process to prevent too optimistic predictive estimates using the holdout set. Therefore, the ML analysis was performed on 58 resamples using the same five ML algorithms as for the Cyclists dataset. The entire analysis finished in 31 min and 1 s. The top performing model was built on the resample with 47 donors that shared 13 features with the naive Bayes algorithm (train AUROC 0.73 and test AUROC 0.71) (Figure 1D, Table S7, and Data S3).

We also applied SIMON to (1) a dataset with a large number of features measured using whole-metagenome shotgun sequencing of fecal samples (Zeller dataset, Table S8) and (2) the liver hepatocellular carcinoma dataset containing RNA-sequencing data from The Cancer Genome Atlas (TCGA) with an unbalanced sample distribution of tumor and adjacent normal tissue samples (LIHC dataset, Table S9). Both datasets are described in the Experimental Procedures. For the Zeller dataset, models were built using ML algorithms known to perform well in situations where more features were measured than individuals, such as shrinkage discriminant analysis,^49^ high-dimensional discriminant analysis,^50^ and neural network with feature extraction.^51^ Two additional algorithms were included, svmLinear2 and LogitBoost. The complete analysis was performed in less than 1 min (0:38 min). The sda algorithm built the model with the highest performance (train AUROC 0.86 and test AUROC 0.81), having a higher performance measure than the published LASSO linear regression model^45^ (train AUROC 0.84 and test AUROC 0.85) (Figure 1E, Table S10, and Data S4). For the LIHC dataset we used the same five ML algorithms as for the Zeller dataset, and analysis finished in 11 min and 30 s. For such a highly unbalanced dataset the precision-recall AUC (prAUC)^52^ is a much better performance measurement than AUROC that reported near-perfect performance (Figure 1E). The prAUC provides information on how well the model correctly detects cancer cells, while it is less stringent on the evaluation of healthy cells. To avoid obtaining overly optimistic prediction results (often observed on unbalanced datasets), we ranked models based on the prAUC of the training set (Table S11). The model that had the best performance was built using the svmLinear2 algorithm (train prAUC 0.83) and it also performed well on the holdout test set (prAUC 0.73) (Data S5).

“Drowsiness” contributed the most to the top-performing SISA model, confirming the findings from the original study^42^ (Table S12). To standardize the process for evaluation of the features and their contribution to the models, we implemented the variable importance score evaluation functions from the caret library.^33^ This allows users to compare features selected across models. In the case of the SISA dataset, drowsiness contributed the most in all of the models built (Table S13), indicating the importance of this symptom and its correlation with hospitalization. The features that contributed the most to the Cyclists model were total memory, unswitched memory, and naive B cells; recent thymic emigrants; CD8^+^ T cells with TEMRA phenotype; and regulatory T cells (CD25^+^ Foxp3^+^ CD4^+^ T cells) (Table S14; Video S4). In comparison to age-matched physically inactive individuals (non-cyclists), the master cyclists had increased frequencies of recent thymic emigrants, naive B cells, and CD3 cells, and decreased frequencies of memory B cells and CD8 T cells with TEMRA phenotype, confirming that aging of the immune system, i.e., immunosenescence, can be reduced by high levels of physical activity^43^ (Figures 1F and S1). To further explore the relationship between selected features, users can perform correlation analysis to reveal highly correlated features (Figure 1G; Supplemental Information, Video S5). Naive and memory B cells were identified as being highly correlated (Figure 1G), as expected, since these subsets were determined from the same flow cytometry plots and their relationship is inversely correlated. Removal of those highly correlated features can help to build more accurate models. Removal of naive B cells resulted in building a predictive model with the same performance measurements as the model built on the entire dataset (train AUROC 0.99 and test AUROC 1) (Table S15), while removal of total memory B cells lowered the accuracy estimates (train AUROC 0.98 and test AUROC 1) (Table S16), indicating the importance of memory B cells to discriminate between master cyclists and non-cyclists. In the VAST dataset, individuals with higher IgA, IgA1, IgA2, and IgG2 titers against native Vi polysaccharide (nViPS) antigen and higher IgA and IgG3 titers against biotinylated Vi polysaccharide (ViBiot) on the day of the challenge were protected against the typhoid challenge, supporting the data from univariate analysis^44^ (Table S17 and Figure S2). Moreover, using the clustering function of SIMON's exploratory analysis module, we quickly found that the IgA2 signature dominates the responses after vaccination with a purified Vi polysaccharide (Vi-PS), while the IgG2 signature was dominant for the Vi tetanus toxoid conjugate (Vi-TT) vaccine^44^ (Figure 1H, Supplemental Information). For the Zeller dataset, the same features as originally reported^45^ contributed the most to the model, including *Fusobacterium nucleatum* and *Peptostreptococcus stomatis* (Table S18). The features that contributed the most to the LIHC model were well-known genes identified to be upregulated in LIHC, such as *GABRD* and *PLVAP*,^53^ and genes enriched in adjacent normal tissue samples, *ANGPTL6*,^54^ *VIPR1*,^55^ and *OIT3*,^56^ as a typical signature for healthy liver tissue (Table S19, Figure S3).

Video S4. Feature Selection in SIMON

Video S5. Exploratory Features in SIMON

## Discussion

We have developed SIMON, a powerful software platform for data mining that facilitates pattern recognition and knowledge extraction from high-quality, heterogeneous biological and clinical data, especially where there are missing data, an unbalanced distribution, and/or high dimensionality. It can be used for the identification of genetic, microbial, and immunological correlates of protection and it can help in guiding the further analysis of the biomedical data.

Over the past years, technological advances have enabled the generation of large amounts of data at multiple scales. Monitoring and analyzing these complex datasets is particularly important in the biomedical sciences, as they serve to advance knowledge about health and disease, as well as predicting clinical outcomes in advance of their occurrence. Despite major clinical and economic consequences of these approaches, due to the lack of powerful analytical tools that can be used by the average biomedical researcher, the translation of such knowledge can be extremely slow. Although several commercial softwares are available, for instance, Google's cloud-based AutoML (https://cloud.google.com/automl), DataRobot (https://www.datarobot.com/), BigML (https://bigml.com/), MLjar (https://mljar.com), and RapidMiner (https://rapidminer.com/), they come at a high price and have hidden ML methods and algorithms, and thus have not been adopted by the biomedical community. In academia, open-source ML software is being developed, for example, Waikato Environment for Knowledge Analysis (WEKA)^57^ (https://www.cs.waikato.ac.nz/∼ml/weka/), Orange^58^ (https://orange.biolab.si/), the Konstanz Information Miner (KNIME)^59^ (https://www.knime.com/), and ELKI^60^ (https://elki-project.github.io/). For the development of SIMON, our aim was to integrate the capabilities of commercial software openly and freely for everyone. The currently available open-source software offers only a limited number of the most commonly used ML algorithms using a user-friendly graphical interface, while focusing on the manual configuration to achieve optimal model predictive performance. Therefore, usage of these softwares requires extensive knowledge of the ML process, so the primary users are data scientists, statisticians, and ML experts. In contrast, commercial software versions are implementing an automated ML (autoML) process that rapidly builds high-performance models by identifying the optimal ML method, including the selection of an appropriate algorithm, optimization of model hyperparameters, and evaluation of the best-performing models.^61^ AutoML improves the efficiency of the ML process, and the resulting models often outperform hand-designed ones.^61^^,^^62^ By implementing this simplified application of ML in SIMON, non-experts can build high-performing models. In addition to auto-Weka, which provides a graphical user interface for an open-source version of the autoML^63^ constrained to the most commonly used ML algorithms, there are also frameworks, such as auto-sklearn,^64^ TPOT,^65^ and AutoPrognosis,^66^ highlighting the importance of the application of autoML to biomedical data. Although the process of selection of algorithms and optimization of hyperparameters is automated in SIMON, the data pre-processing steps and exploratory analysis of the resulting models require background knowledge about the data distribution and correlation, transformations, and processing steps before running the analysis and evaluation of predictive models built by SIMON.

Another advantage of commercial over open-source software, which we implemented in SIMON, is the architecture of commercial software supports running ML processes in the cloud or in the server mode. The SIMON server edition provides an option for web-based collaborative efforts that reflect the necessity to accommodate the increased size of datasets, the complexity of models, and data privacy concerns, for instance, for sharing human genomic data. Because the integration of biomedical data across clinical studies and research groups around the globe can enable training of more detailed models and lead to higher-quality insights, the SIMON server mode offered as an open-source version of ML software is a valuable tool.

SIMON is developed as a modular open-source software, which allows us to extend our work by integrating novel features in the future versions. Although this version of the software can analyze multiple datasets, ranging from clinical and cytometry data to transcriptome, microbiome, and proteome with missing data, high dimensionality, and unbalanced distributions, future multi-omics datasets integrating different modalities or time-series datasets will require new methods, such as ensemble methods, automated feature selection,^67^ and forecasting algorithms. Moreover, as the number of predictive models built using biomedical data increases, SIMON will be able to identify which algorithms work the best for a particular dataset.

Overall, SIMON is designed to provide a uniform knowledge discovery interface adaptable to the increasing size of biomedical datasets that can allow even non-expert biomedical researchers to solve important problems when faced with complex and heterogeneous datasets.

## Experimental Procedures

### Resource Availability

#### Lead Contact

The lead contact for this article is Adriana Tomic (info@adrianatomic.com).

#### Materials Availability

Datasets used in Figure 1 were either obtained directly from authors (VAST^44^ and Cyclists^43^ datasets) or downloaded from publications^42^ (SISA dataset) and R packages (Zeller dataset from the MetagenomicData^46^ and LIHC from the GSEABenchmarkeR^47^) with help from the authors. The SISA dataset contains data from 543 individuals hospitalized due to arboviral infection with dengue, chikungunya, or Zika virus from a surveillance study in Ecuador collected from 2013 to 2017. In the SISA dataset we excluded columns with high level of missing values (pregnancy, “WomPreg,” and complete blood count test, which was not performed for all donors and includes the columns “PLT_count,” “Lymphocytes,” “CBC_N%,” “WBC_calc,” and “CBC_HCT”). In addition, nine donors with missing values were removed. The final SISA dataset after removal of columns and rows with missing values is available as Table S2. The Cyclists dataset contains data from the immune responses of 120 elderly individuals with a high-level of physical activity, i.e., master cyclists, and 75 age-matched controls with a low level of physical activity (non-cyclists) analyzed using flow cytometry (Table S4). The VAST dataset contains data from 72 individuals enrolled in the clinical study to evaluate humoral responses in a typhoid vaccine efficacy trial in a controlled human infection model. Only day 0 (day of the challenge) log-transformed data were used and are available for download as Table S5. Individuals were vaccinated with either a purified Vi-PS vaccine (35 individuals) or the Vi-TT vaccine (37 individuals) 1 month prior to oral challenge with live *Salmonella* Typhi. Of 72 individuals, 26 developed an acute typhoid infection following challenge. The Zeller dataset contains information on the microbiome species abundance in healthy individuals and colorectal cancer patients (Table S8). The data were accessed through the MetagenomicData package. In total 184 individuals were included, of which 93 were healthy controls and 91 colorectal cancer patients. The LIHC dataset obtained from the GSEABenchmarkeR package contains RNA expression data from 374 LIHC cells and 50 adjacent normal cells (Table S9).

#### Data and Code Availability

The source code of SIMON is available at https://github.com/genular/simon-frontend. All data used in SIMON analysis are available as Supplemental tables, while ML models are available as Supplemental data in the RData format. Datasets are available for the download from the Zenodo data repository: VAST (Zenodo Data: http://doi.org/10.5281/zenodo.4121322),^68^ SISA Zenodo Data: http://doi.org/10.5281/zenodo.4121831),^69^ Cyclists (Zenodo Data: http://doi.org/10.5281/zenodo.4115626),^70^ Zeller (Zenodo Data: http://doi.org/10.5281/zenodo.4121516),^71^ and LIHC (Zenodo Data: http://doi.org/10.5281/zenodo.4121594).^72^

### Installing SIMON

SIMON can be installed directly from the GitHub (https://github.com/genular/simon-frontend) or a pre-built version can be installed from DockerHub (https://www.docker.com/). Users need to install Docker (version 17.05 or later required) following instructions available on the Docker website (https://docs.docker.com/). Installation instructions for Windows (https://docs.docker.com/docker-for-windows/install/), MacOS (https://docs.docker.com/docker-for-mac/install/), and Linux (https://docs.docker.com/install/linux/docker-ce/ubuntu/) are provided. After Docker installation, users must download and run a SIMON image from DockerHub. To do that users must run Terminal on Linux and MacOS or Windows Power Shell if using Windows OS and type following command:

docker run --rm --detach --name genular --tty --interactive --env IS_DOCKER='true' --env TZ=Europe/London --volume genular_data:/mnt/usrdata --publish 3010:3010 --publish 3011:3011 --publish 3012:3012 --publish 3013:3013 genular/simon:latest

Variable ‘TZ=’ stands for time zone and can be replaced with appropriate time zone. Once the command is executed, SIMON will be downloaded and started. To access SIMON, open a web browser (Firefox recommended, available at https://www.mozilla.org/firefox/) and type http://localhost:3010. Create an administrator account. SIMON will run until you shut down/restart your computer or stop it manually using the following command: docker stop genular. Advance instructions for installing a server version are provided on our GitHub page.

## Supplemental Information

Document S1. Supplemental Experimental Procedures and Figures S1–S3

Table S1. List of Machine Learning Algorithms Implemented in SIMON

Table S2. SISA Dataset

Table S3. List of Models Built for the SISA Dataset

Table S4. Cyclists Dataset

Table S5. VAST Dataset

Table S6. List of Models Built for the Cyclists Dataset

Table S7. List of Models Built for the VAST Dataset

Table S8. Zeller Dataset

Table S9. LIHC Dataset

Table S10. List of Models Built for the Zeller Dataset

Table S11. List of Models Built for the LIHC Dataset

Table S12. List of Features and Their Variable Importance Score in the SISA Dataset Selected in the Top Performing Model

Table S13. List of Features and Their Variable Importance Score in the SISA Dataset Selected in All Models

Table S14. List of Features and Their Variable Importance Score in the Cyclists Dataset

Table S15. List of Models Built after Removal of Naive B Cells

Table S16. List of Models Built after Removal of Memory B Cells

Table S17. List of Features and Their Variable Importance Score in the VAST Dataset

Table S18. List of Features and Their Variable Importance Score in the Zeller Dataset

Table S19. List of Features and Their Variable Importance Score in the LIHC Dataset

Data S1–S5

Document S2. Article plus Supplemental Information

## Footnotes

Supplemental Information can be found online at https://doi.org/10.1016/j.patter.2020.100178.
