# Supplementary material for: SIMON: Open-Source Knowledge Discovery Platform
Source: Patterns (N Y). 2021 Jan 8;2(1):100178. doi: 10.1016/j.patter.2020.100178 (PMC7815964; doi:10.1016/j.patter.2020.100178)
Supplement: Document S1. Supplemental Experimental Procedures and Figures S1–S3 [file mmc1.pdf]

PATTER, Volume *2*

Supplemental Information

SIMON: Open-Source Knowledge Discovery Platform

Adriana Tomic, Ivan Tomic, Levi Waldron, Ludwig Geistlinger, Max Kuhn, Rachel L. Spreng, Lindsay C. Dahora, Kelly E. Seaton, Georgia Tomaras, Jennifer Hill, Niharika A. Duggal, Ross D. Pollock, Norman R. Lazarus, Stephen D.R. Harridge, Janet M. Lord, Purvesh Khatri, Andrew J. Pollard, and Mark M. Davis

Supplemental materials

Supplemental Experimental Procedures

Step-by-step instructions how to run SIMON analysis for the following use cases: (1) Identifying clinical biomarkers that can predict the severity of the arboviral infection severity; (2) Predicting antibody signature to mediate protection against *Salmonella* Typhi challenge infection; (3) Identifying cellular immune signature associated with high-level of physical activity; (4) Building predictive model for the early-stage detection of colorectal cancer using microbiome; and (5) Building predictive model for detection of liver hepatocellular carcinoma cells using transcriptome data.

Use case 1. Identifying clinical biomarkers that can predict the severity of the arboviral infection severity.

Step 1. Uploading data. SISA dataset (available as **Supplementary table 2**) needs to be uploaded as CSV file in the following format: donors/samples in rows and features that were measured (i.e. clinical measurements) in columns (*'Click here'* red arrow on image below). One of the columns contains information about the outcome, in this case this is the column named *'Hospitalized'* and the outcome is labelled with zero if '*non-hospitalized'* and with one is *'hospitalized'*. Note that SIMON can analyze data using either text or numeric values for the outcome variable.

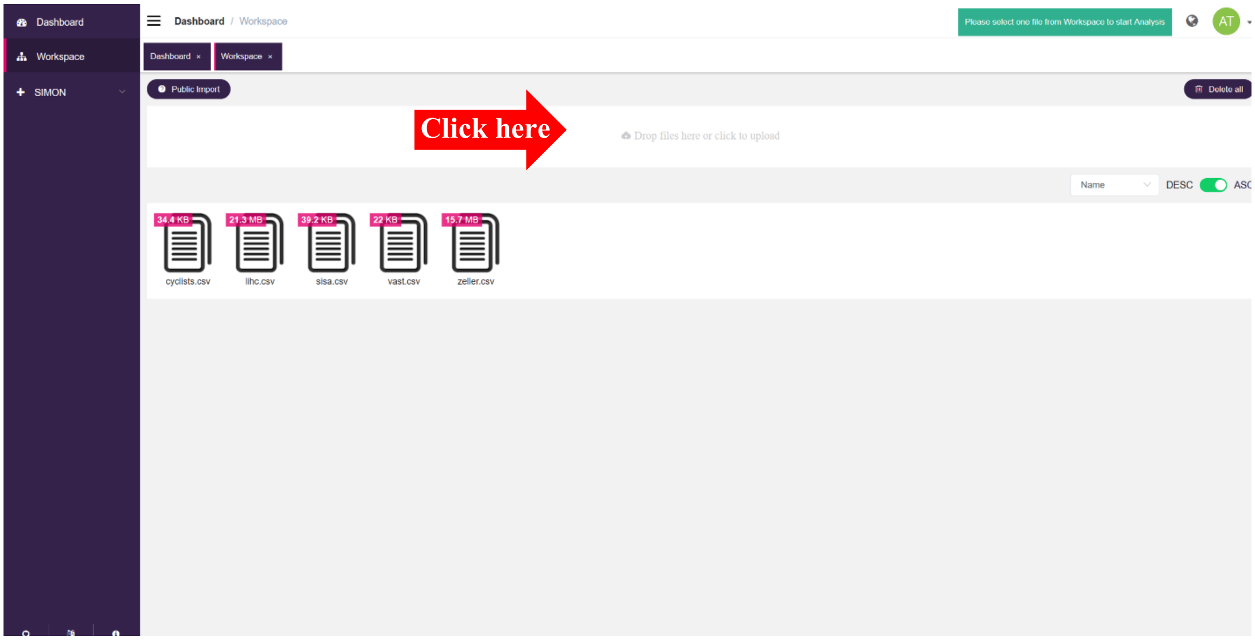

Step 2. Selecting dataset. To start the analysis, user must select the SISA dataset by clicking the document icon (A). The selected SISA dataset will be highlighted in grey and in the upper right-hand side the green

tab will show the name of the SISA dataset (B). Now in the left-hand side menu (in purple) the ‘*Analysis’* tab becomes available (C).

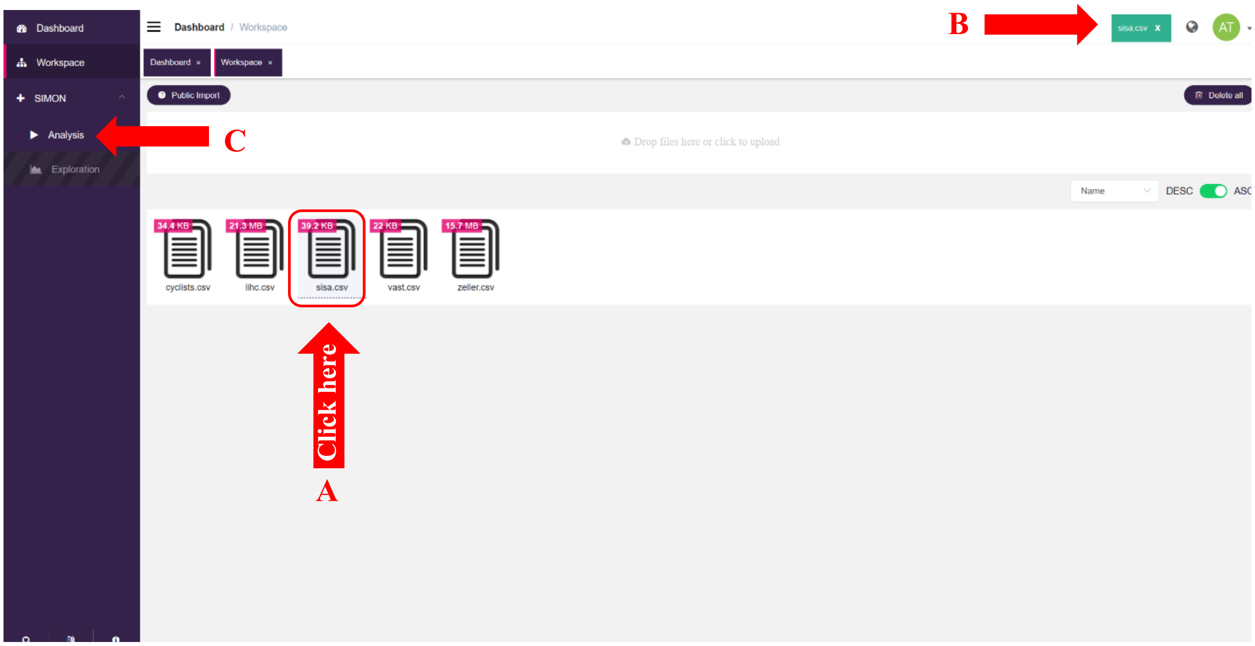

Step 3. Setting-up analysis. Once SISA dataset is selected, users can start with the analysis by clicking the *‘Analysis’* on the left-hand side menu (in purple). This opens new window *‘Analysis’* where users can select analysis parameters and ML algorithms. For the SISA dataset, in the Box 1, users must select all predictor variables by clicking the button next to the input form (A) since we want to use all clinical measurements for the prediction model. Alternatively, if users want to select only some features, by clicking in the input field *‘Predictor variables’* first 50 available columns are shown in the drop-down menu and users can choose which columns they want to use for analysis. If there are more than 50 columns available, users can type which columns they want to use. Next, we select the outcome we want to predict in the ‘*Response’* input field, in the SISA dataset that is the *‘Hospitalized’* column (B). We then select which columns to exclude (C). In the SISA dataset we have excluded column without any information for the predictive model (donor identification numbers in the *IDindex* column). The initial SISA dataset is split into training (85% of the data) and test sets (15% of the data) (D). Finally, for the pre-processing step, data was centered (mean subtracted from values) and scaled (values divided by standard deviation) (E).

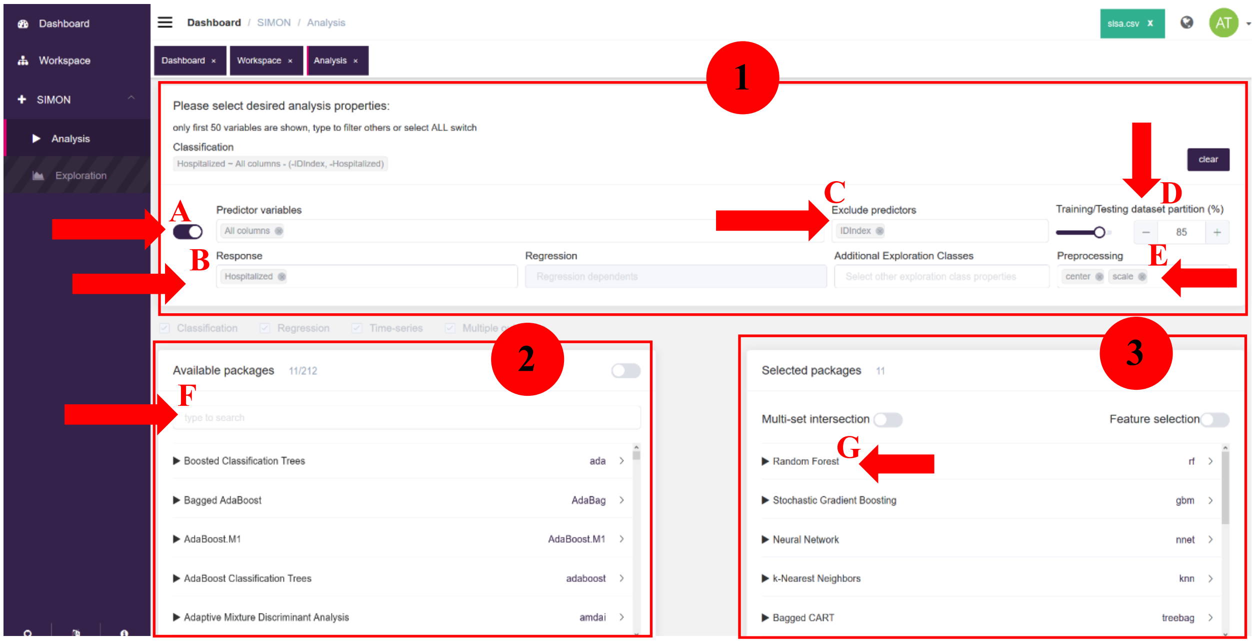

Users can choose which pre-processing functions they want to apply. Available pre-processing functions are Box-Cox transformation (BoxCox), power transformation (expoTrans), Yeo-Johnson transformation (YeoJohnson), subtract mean from values (center), divide by standard deviation (scale), normalize values (range), K-nearest neighbors Imputation (knnimpute), Imputation using Bagging of regression tress (bagImpute), median impute (medianImpute), prinicipal component analysis (pca), data projected onto a unit circle (spatialSign), correlation filtering (corr), remove zero-variance (zv), remove near zero-variance (nzv) and exclude predictors that have only one unique value (conditional).

In the Box 2, users select which ML algorithms to use, while 5 of the default ML algorithms are already selected in the Box 3. For the analysis of the SISA dataset, we will, in addition to five already selected, select additional six ML algorithms: shrinkage discriminant analysis, treebag, k nearest neighbors, random forest, stochastic generalized boosting model and neural network. Name of the ML algorithm is typed in the input field (F). The full names of the packages for the selected algorithms are: *'Shrinkage discriminant analysis'*, Shrinkage discriminant analysis (sda); *'Treebag'*, Bagged CART; *'k nearest neighbors'*, k-Nearest Neighbors (knn); *'Random forest',* Random forest (rf); '*Stochastic generalized boosting model'*, Stochastic gradient boosting (gbm) and *'Neural network',* Neural network (nnet). Once the name of the algorithm is typed and user clicks on the desired package, that algorithm is automatically added to the list of selected algorithms in the Box 3. Note, that sometimes different R packages are available for same algorithm, as it is the case for Random forest algorithm. SIMON allows users to inspect selected algorithms by clicking on their names (G). Users then obtain additional information about the algorithm (H) and they

can click to obtain the reference to the original publication (I) to be sure that they select appropriate algorithms.

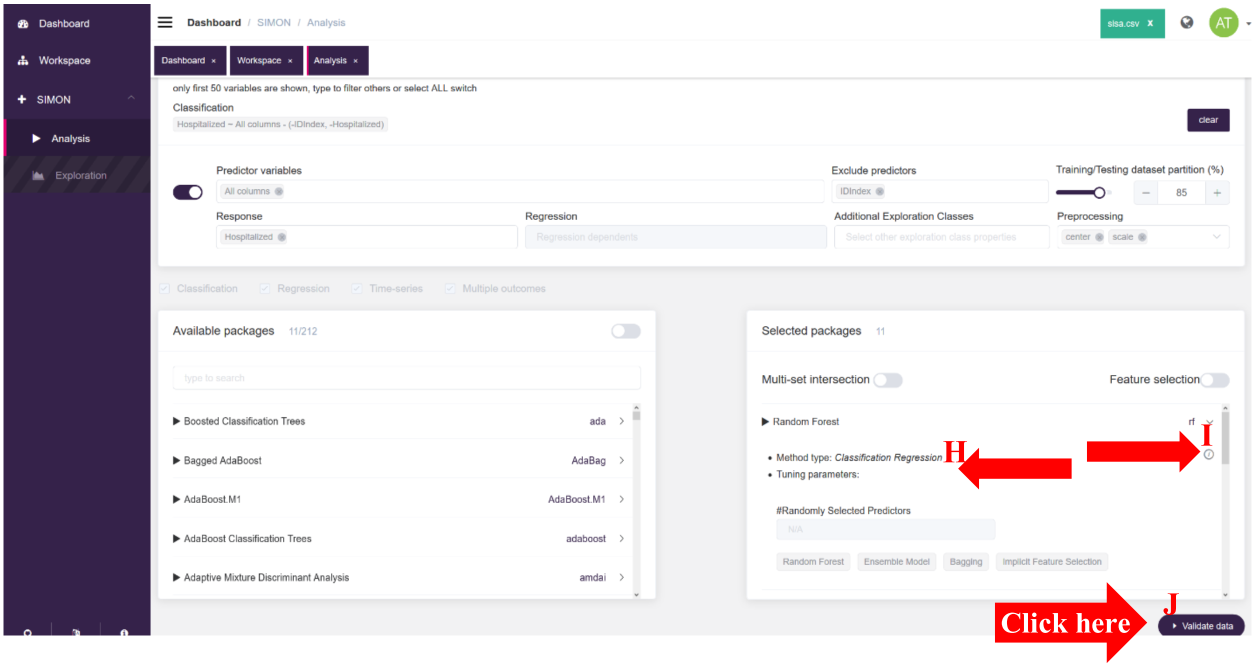

Finally, analysis is initiated by clicking the *'Validate data'* button (J). The following screen shows and analysis is started by clicking on the *'Process'* button (K).

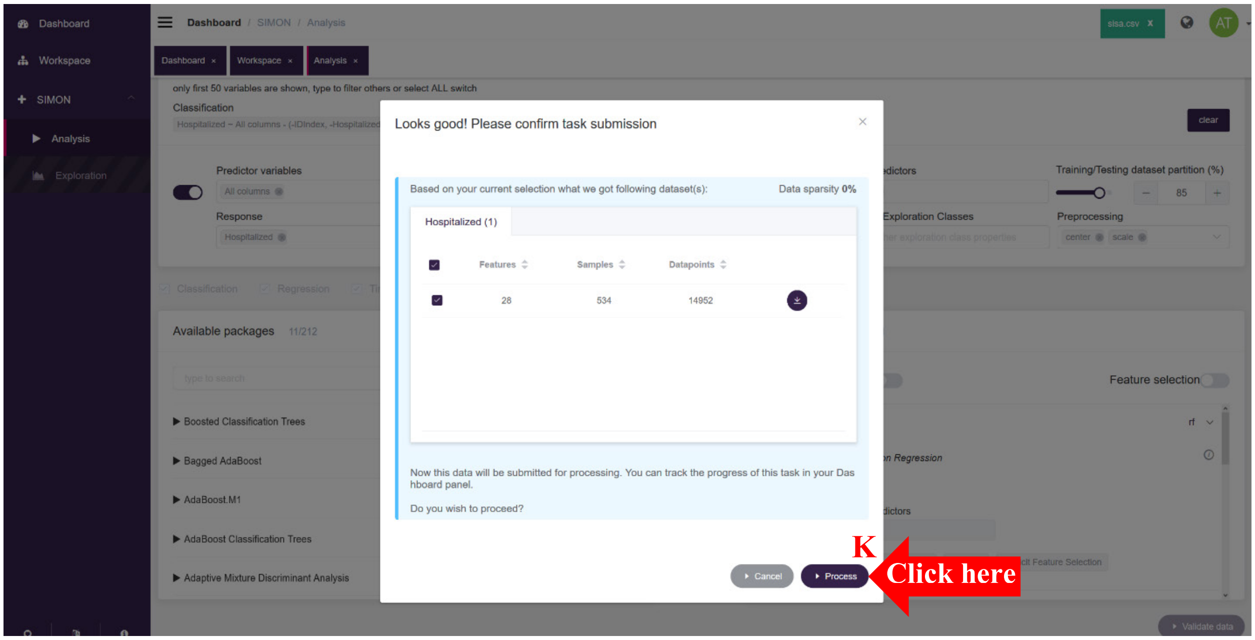

New window *'Dashboard'* will open immediately and SISA analysis will be created and initiated (L). To assess the current status of the analysis, we look under *'Status'* column (M), *'Processing'* means that the analysis is going, once analysis is complete it says *'Completed'* in green. *'Sparsity (%)'* column tells us the

percentage of missing values in the dataset; *'Models processed'*, number of processed models; *'Successful models'*, number of successfully finished models. In the *'Operations'* column users can get information about the models and their performance measures during the analysis (first button, "circled *i*" icon), download dataset (second full green button) and delete the analysis (third red button).

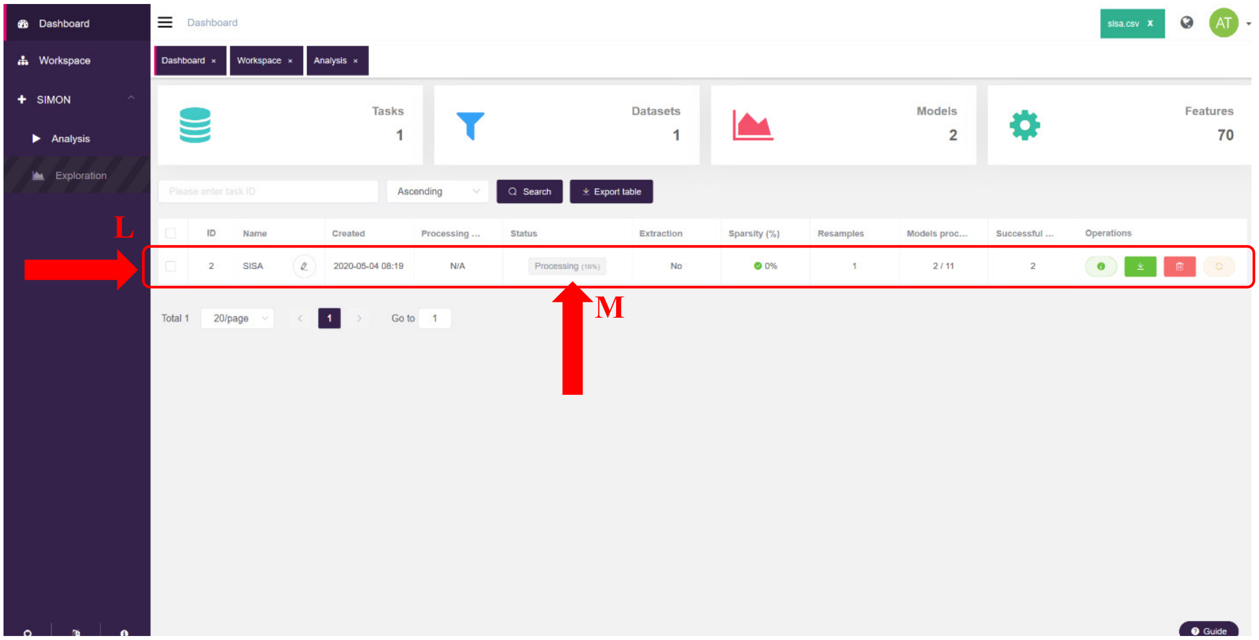

Step 4. Model evaluation and selection. After the analysis is done, users can explore built predictive models by clicking on the checkbox next to the SISA analysis row (A). Upon selection of SISA analysis row, the *'Exploration'* tab (B) becomes available in the menu on the left-hand side. By clicking on the *'Exploration'* tab, new window opens where users can explore built models.

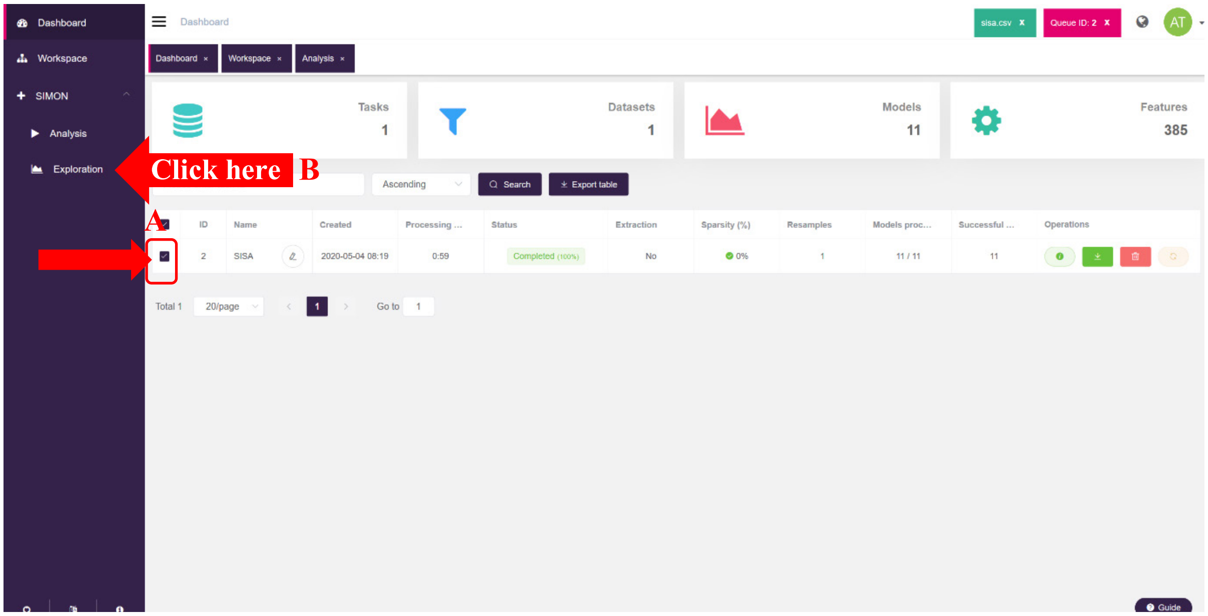

Now, in the new window, users select desired performance measurements by clicking on the drop-down menu in the input Box 1. SIMON calculates different performance measurements for training set (train AUC, train F1, train prAUC, train recall, train precision, train sensitivity and train specificity) and test set (accuracy, F1, kappa, predict AUC, predict prAUC, precision, recall, sensitivity and specificity). For the SISA dataset, we choose AUROC as performance measurement by selecting train AUC and predict AUC. Now, we click in the dataset in the Box 2 (C). This opens Box 3 containing table of all models that were built. We order models based on the train AUC value (D). The model built with the highest train AUC was built with the *sda* algorithm. To compare models, users select desired models by clicking the check box next to each model. We will compare top five models by clicking in the 'select all' check box (E). Once all five models are selected, users can download the table with all models and performance measurements as CSV file and models with the data as RData objects by clicking on F. The initial dataset, training and test set can be saved as CSV files by clicking the download icon next to the dataset row in the Box 2 (G).

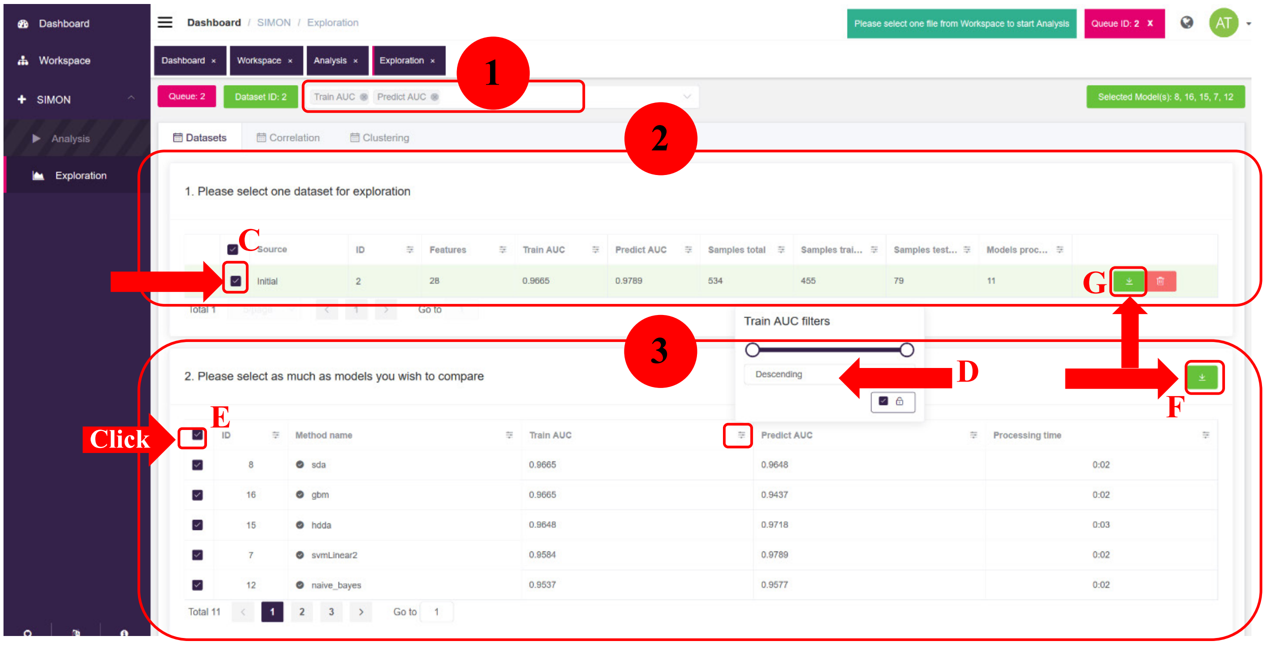

By selecting models to compare, the *'Training Summary'* tab will appear below Box 3 (H). Users must select at least two models for the tab to appear. Here, users visualize model comparison and can download box plots graphs showing performance measures calculated for the training set (I) and ROC plots for the training set (J) for all models selected as SVG files. To select all 11 models, as we did in the Figure 1, users must navigate to pages 2 and 3 and click select all check box.

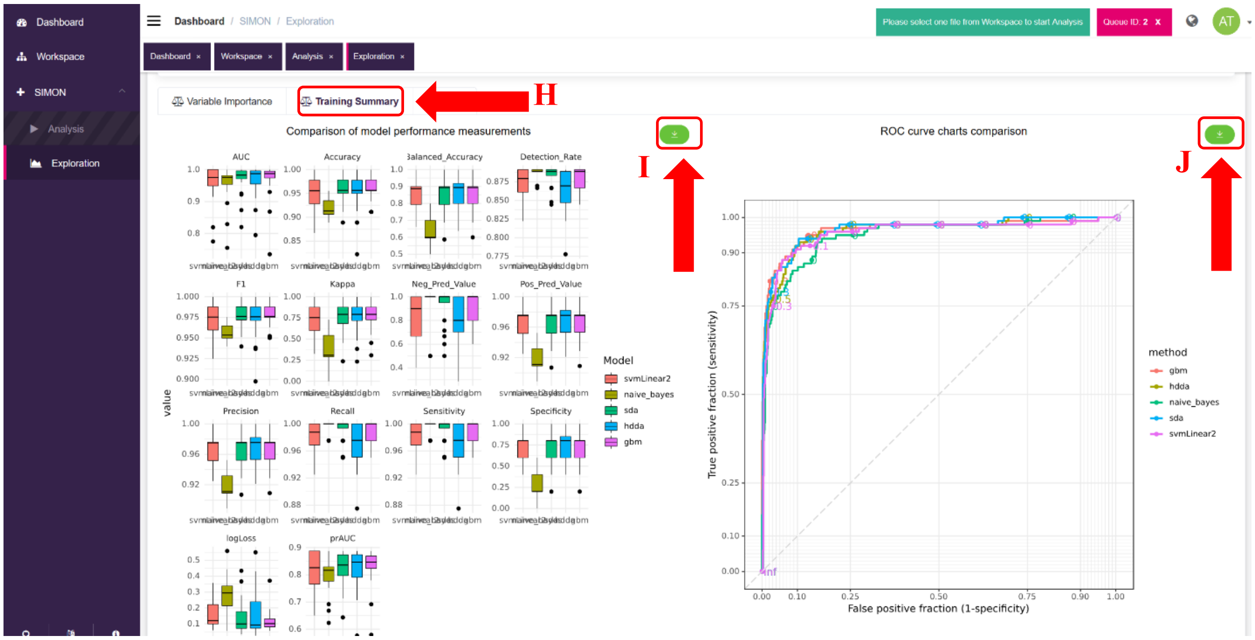

Step 5. Feature selection. To explore features that contributed the most to the sda model, we select only sda model in the Box 3 and click the *'Variable importance'* tab next to the *Training Summary'* tab (A). This opens table where features are ranked based on the Variable Importance Score (*'Score'* column). Features that have Variable Importance score above 50 are highlighted in green (B). The table can be downloaded as the CSV file by clicking download button (C). Users can select two or more models and compare ranking of features across models.

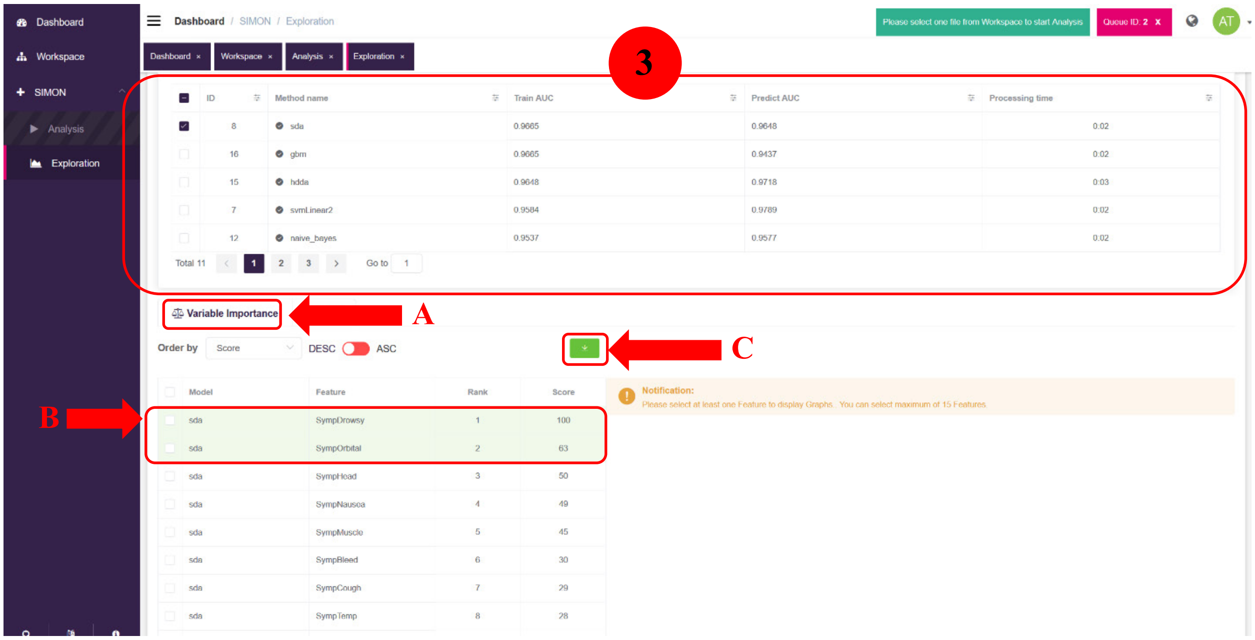

By selecting desired features, users can visualize distribution of data between both groups using dot plots. Plots can be adjusted by selecting *'Theme'*, *'Color'*, font size (*'Font'*), dot size (*'Dot'*) and height/width ratio (*'Ratio'*) as described in the ggplot2 R package (https://ggplot2.tidyverse.org/) (D). To apply changes to the graphs users must press *'Redraw plot'* red button (E) and graphs can be downloaded as SVG files by pressing download button (F).

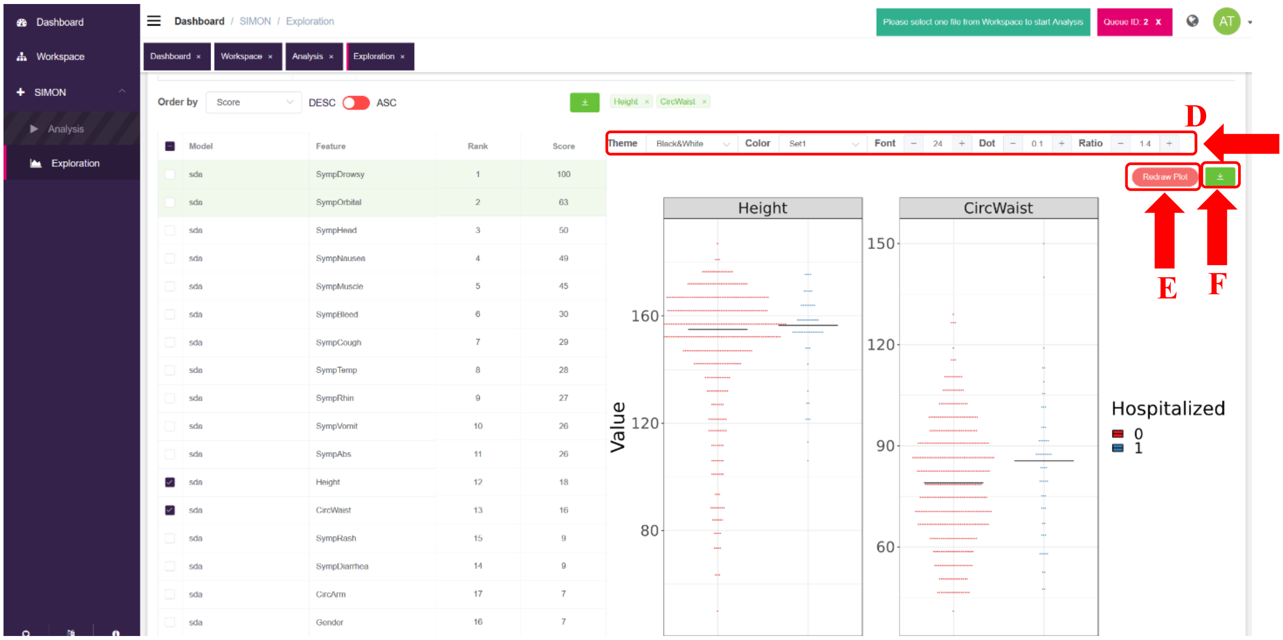

Step 6. Exploratory analysis. In the 'Exploration' window, upon selection of dataset for analysis, two tabs are shown: *'Correlation'* and *'Clustering'* (A). By clicking on *'Correlation'* tab, users can perform correlation analysis on the selected dataset using three different correlation methods (Pearson, Kendall and Spearman) and different parameters can be applied by clicking the *'Plot image'* red button (B). Correlation plot can be saved as SVG file by clicking download button (C). *'Clustering'* tab will be explained in the Use case 2.

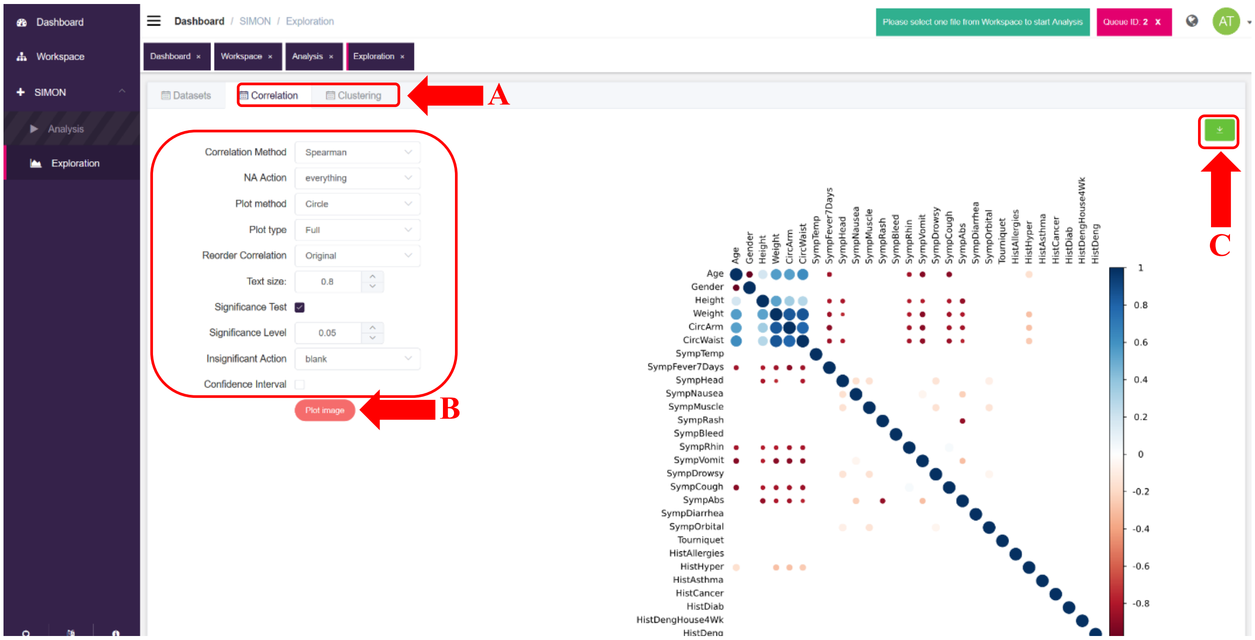

Use case 2. Predicting antibody signature to mediate protection against *Salmonella* Typhi challenge infection.

The first two steps (Step 1. Uploading data and Step 2. Selecting dataset.) are the same as explained under Use case 1, therefore we will start from Step 3.

Step 3. Setting-up analysis. To perform SIMON analysis on the VAST dataset, users must select all predictor variables by clicking the button next to the *'Predictor variables'* input form (A) and *'Diagnosis'* column as the outcome in the *'Response'* input form (B). *'Vaccine'* column is selected under *'Additional Exploration Classes'* (C). The initial dataset is split into training (75% of the data) and test sets (25% of the data) (D) and we applied 'center' and 'scale' as pre-processing steps (E). In total, five ML algorithms were selected (F). Since the VAST dataset has missing values, in the first step of SIMON we will use *'Multi-set intersection'* function (G).

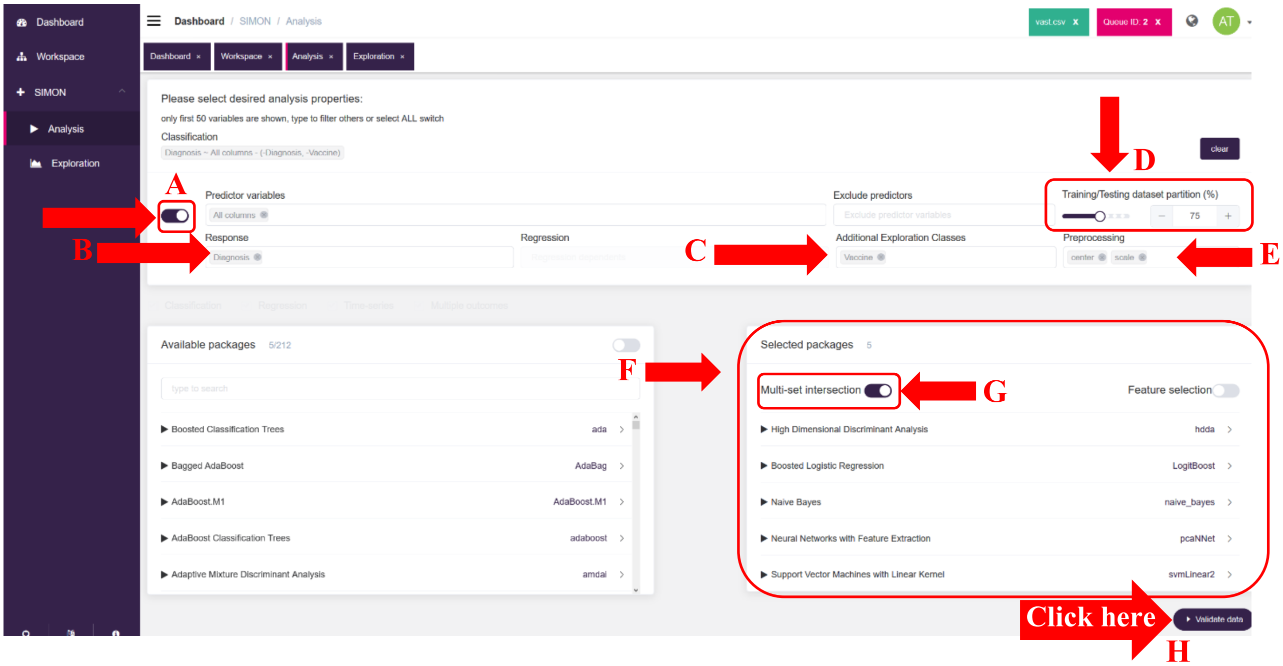

By clicking *'Validate data'* button (H), multi-set intersection function will generate resamples and the pop-up window shows 206 generated resamples with different number of 'Features' and donors ('Samples' column). Each resample can be saved by clicking on the download button and analysis can be performed by selected resamples. In the VAST dataset, we performed analysis on 58 resamples with 40 or more samples in total. Click *'Process'* button to start analysis.

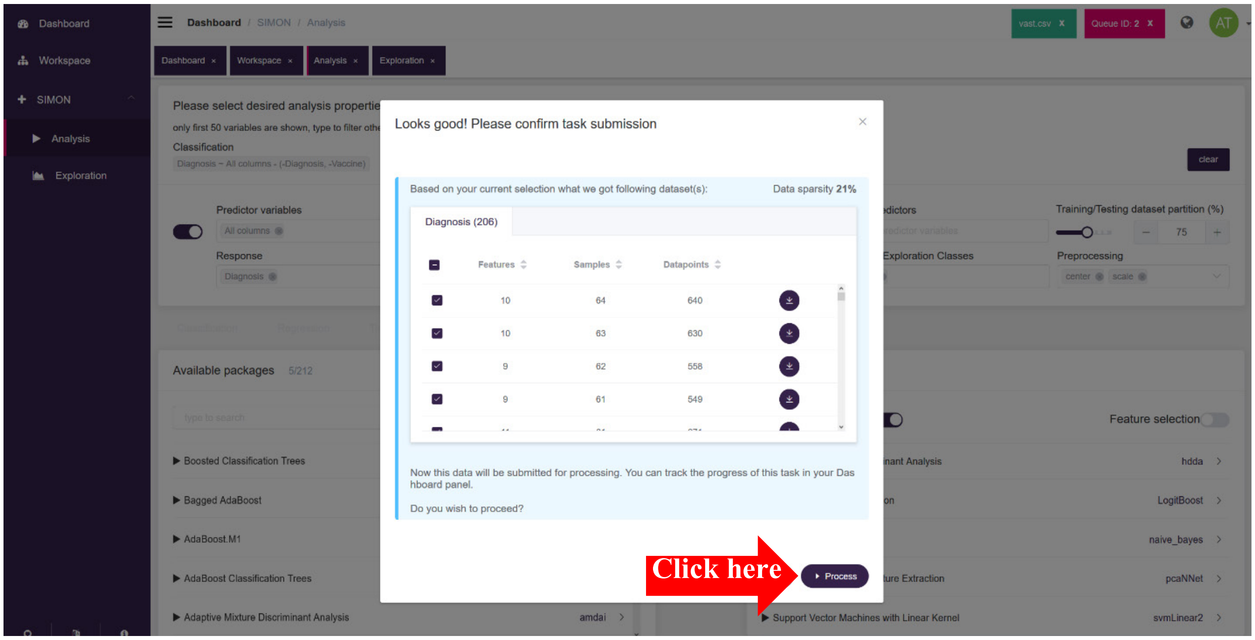

Step 4. Model evaluation and selection. We open *'Exploration'* window (tab becomes available upon selection of VAST analysis row in the *'Dashboard'*) and select train AUC and predict AUC as performance

measurements (A). Then, we order datasets (i.e. resamples) based on the maximum train AUC value and using slider remove all models with train AUC value below 0.72 (B). Now, we select the first dataset with 10 ‘Features’ (C) and 45 ‘Samples total’ (D) and in the table below, we explore all models built for that dataset by ordering models based on the train AUC (E). The model built with the highest performace measurements was built with the pcaNNet algorithm (F), but despite high train AUC value, that model does not perform so well on the unseen test data (predict AUC 0.64). Such a model is considered overfitted. We must examine other resamples to find optimal models with better perfomance on the test data. Each dataset/resample and accompanying split into training and test sets can be saved as CSV file by clicking the download button (G). To highest perfoming model was built on the dataset/resample that has 13 features and 47 donors (samples) (ID 38). We select that dataset and evaluate models using box plots and ROC curves as described for Use case 1.

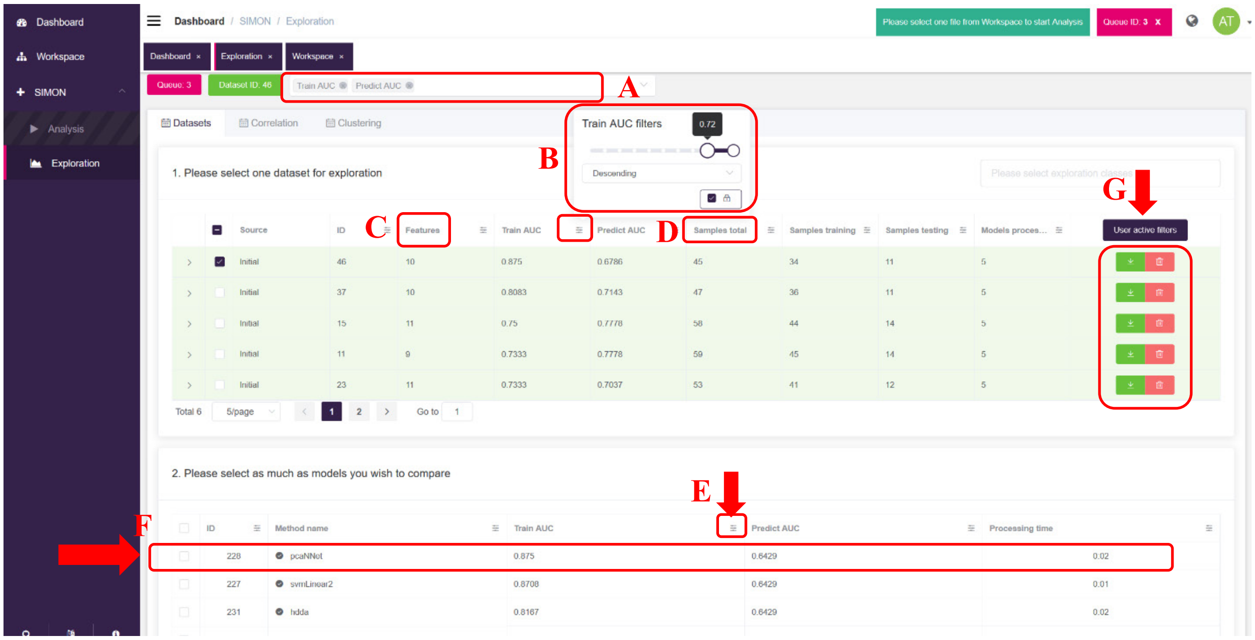

Step 5. Feature selection. Upon selecting the best performing model built with the naïve Bayes algorithm, we can explore the features that contributed the most to this model in the *‘Variable importance’* tab. The variable importance score table can be downloaded as a CSV file and graphs as SVG files by clicking download buttons (A and B).

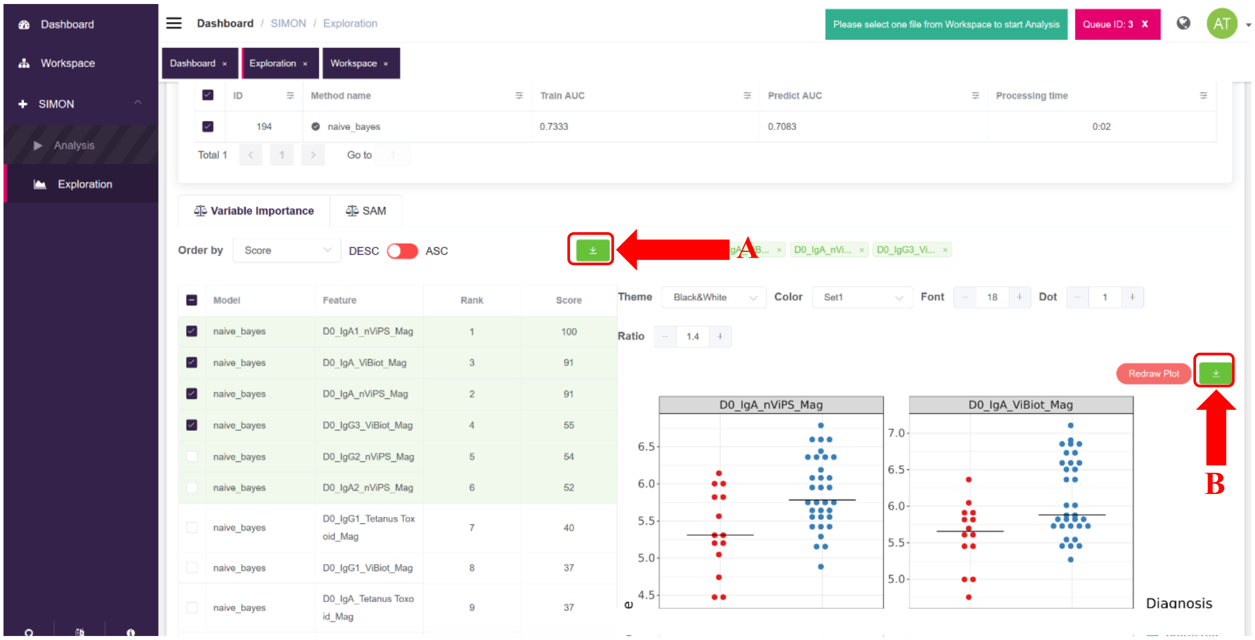

Step 6. Exploratory analysis. In addition to the *'Correlation'* tab explained above in the Use case 1, users can perform clustering analysis in the *'Clustering'* tab. In the VAST dataset we want to explore if the individuals are grouped based on the vaccine they received ('*Vaccine'* column selected under *'Additional Exploration Classes'*). We select *'Diagnosis'* and *'Vaccine'* as columns and 3 top features as rows. After setting up the desired parameters for the clustering analysis, we click *'Plot image'* button (A). The heatmap can be saved as a CSV file by clicking on the download button (B). We can also perform clustering analysis as described above in Use case 1.

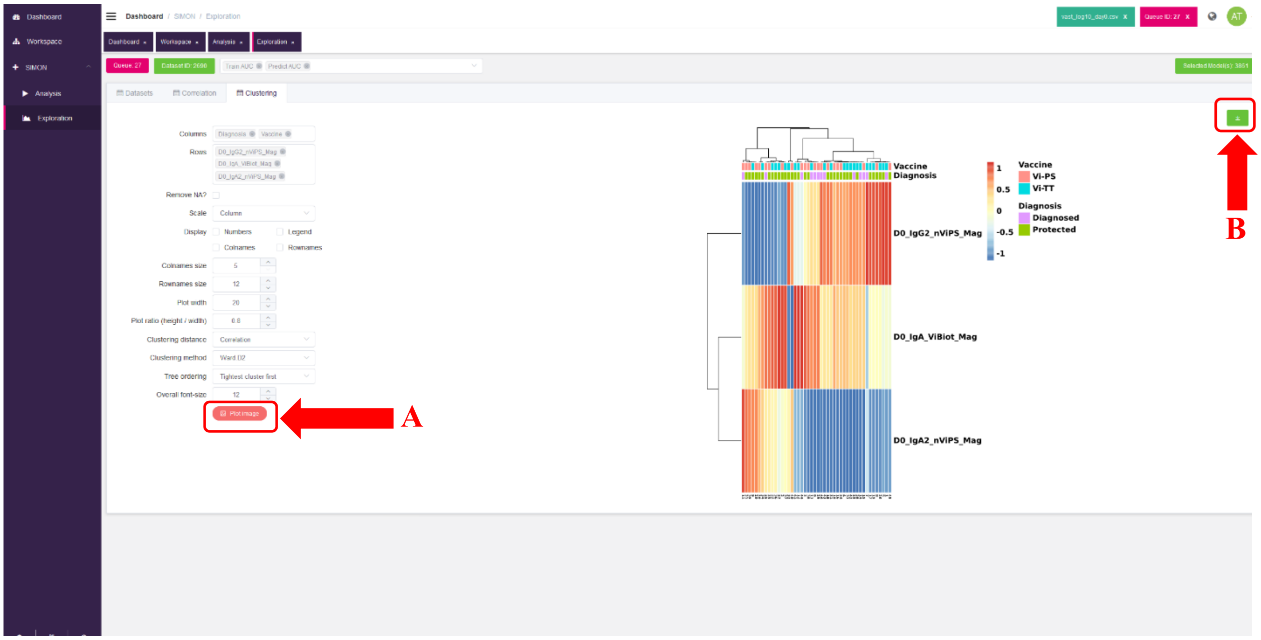

Use case 3. Identifying cellular immune signature associated with high-level of physical activity.

Steps 1-2 and 4-6 were performed as described above for the first two use cases.

Step 3. Setting-up analysis. For the Cyclists dataset, we used all columns as *'Predictors variables'* (A) and outcome column as the *'Response'*(B). Other parameters, training/test split and preprocessing were performed as shown in the screenshot below. Similar to the use case 2, we used multi-set intersection function for the initial dataset to find resamples (C). In total, 146 resamples were identified and analysis was performed using all resamples.

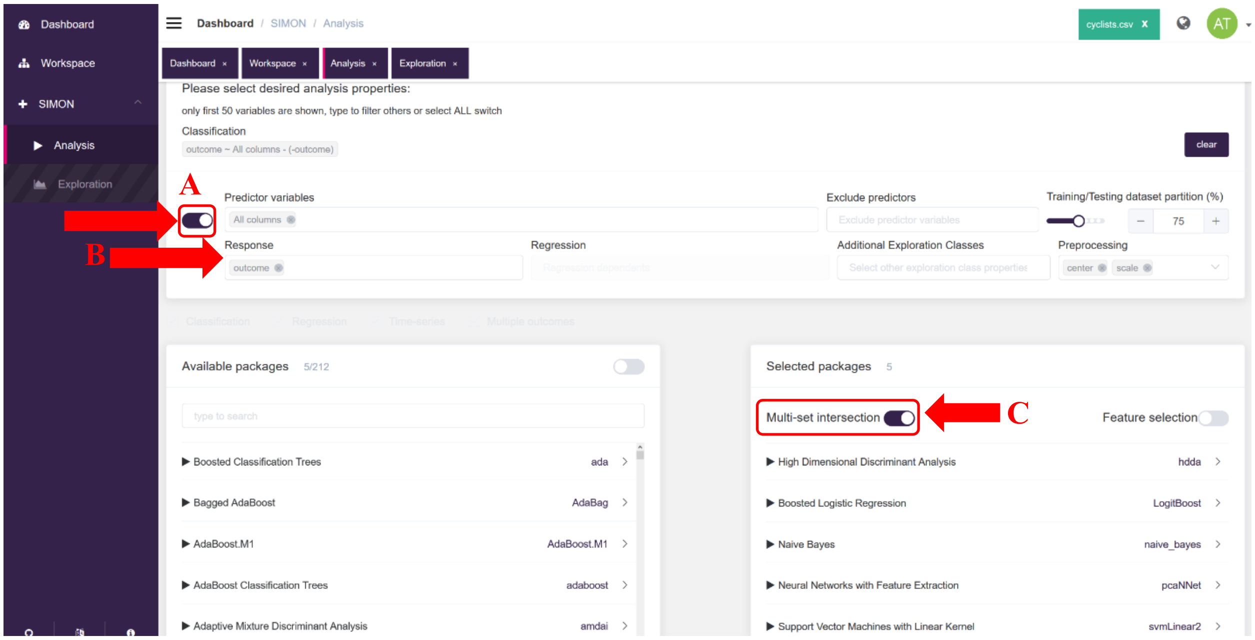

Use case 4. Building predictive model for the early-stage detection of colorectal cancer using microbiome.

Steps 1-2 and 4-6 were performed as described above.

Step 3. Setting-up analysis. After uploading and selecting the Zeller dataset, in the *'Analysis'* window, we selected all columns as *'Predictors variables'* (A) and we typed *'Study condition'* in the *'Response'* input form to find the outcome column (B). The initial dataset was divided 75% into training and 25% test set (C). For the preprocessing we applied '*center*', *'scale'* and remove near zero-variance ('*nzv'*) (D). In total, five ML algorithms were selected (E) and analysis was started by pressing *'Validate data'* button.

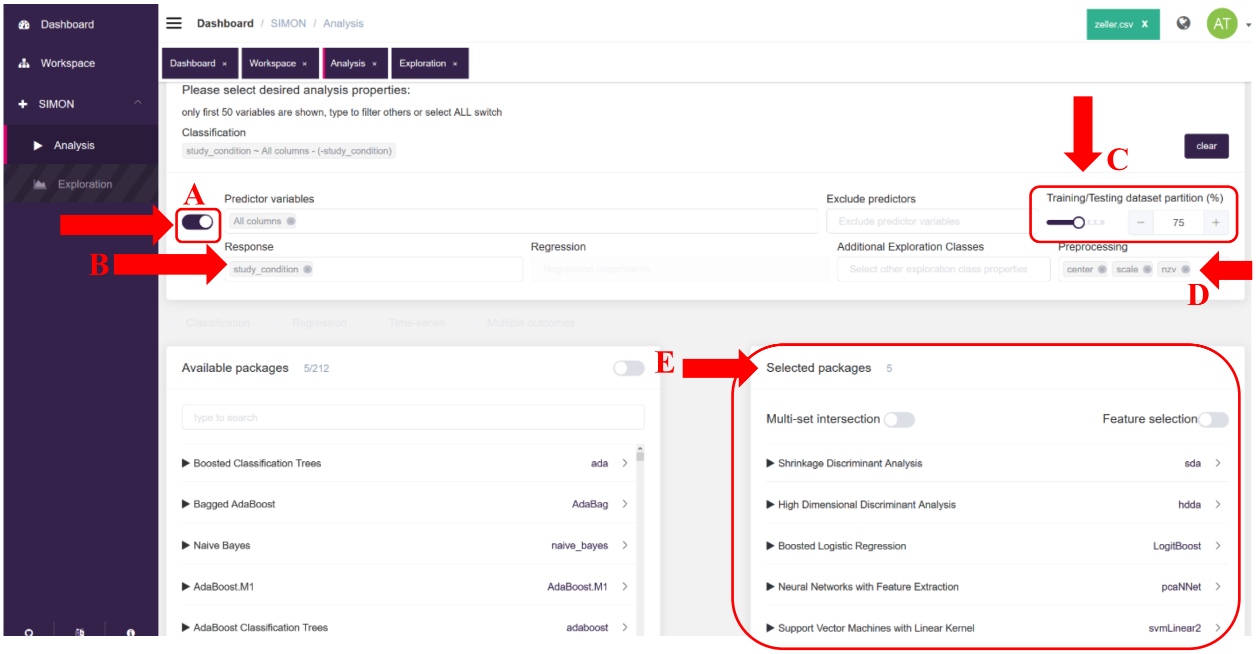

Use case 5. Building predictive model for detection of liver hepatocellular carcinoma cells using transcriptome data.

Steps 1-2 and 5-6 were performed as described above for the other Use cases.

Step 3. Setting-up analysis. For the LIHC dataset, analysis was started with selecting all columns as *'Predictors variables'* (A) and *'Group'* column (tumor or healthy cells) as the *'Response'* (B). The initial dataset was divided 75% into training and 25% test set (C). For the preprocessing we applied '*center*', *'scale'* and remove near zero-variance ('*nzv*') (D). In total, five ML algorithms were selected (E) and analysis was started (*'Validate data'* button).

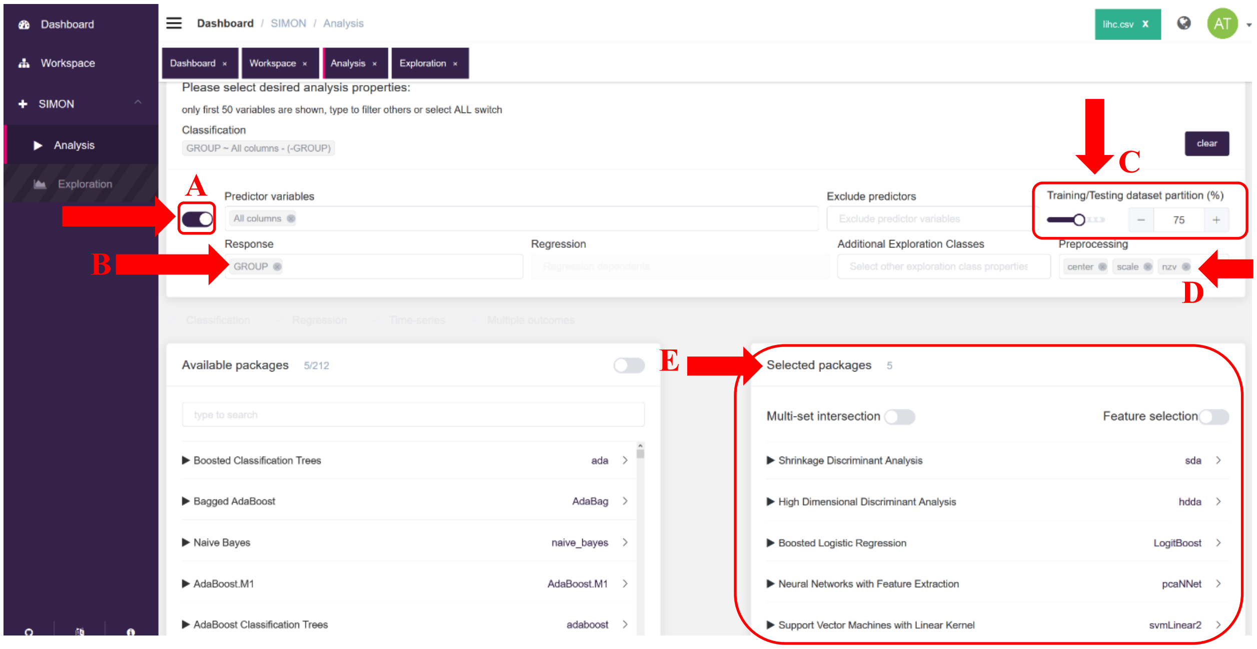

Step 4. Model evaluation and selection. The LIHC is the example of highly imballanced dataset, therefore in the *'Exploration'* window (tab becomes available upon selection of LIHC analysis row in the *'Dashboard'*) and select precision-recall AUC (train prAUC for the training set and prAUC for the test set) (A). The models are then ranked based on the train prAUC (B). The first model that has high train prAUC value, also performed well on the left-out test set. We save the generated model by clicking the download button (C). Visualization of model performance measurements is performed as described for other Use cases.

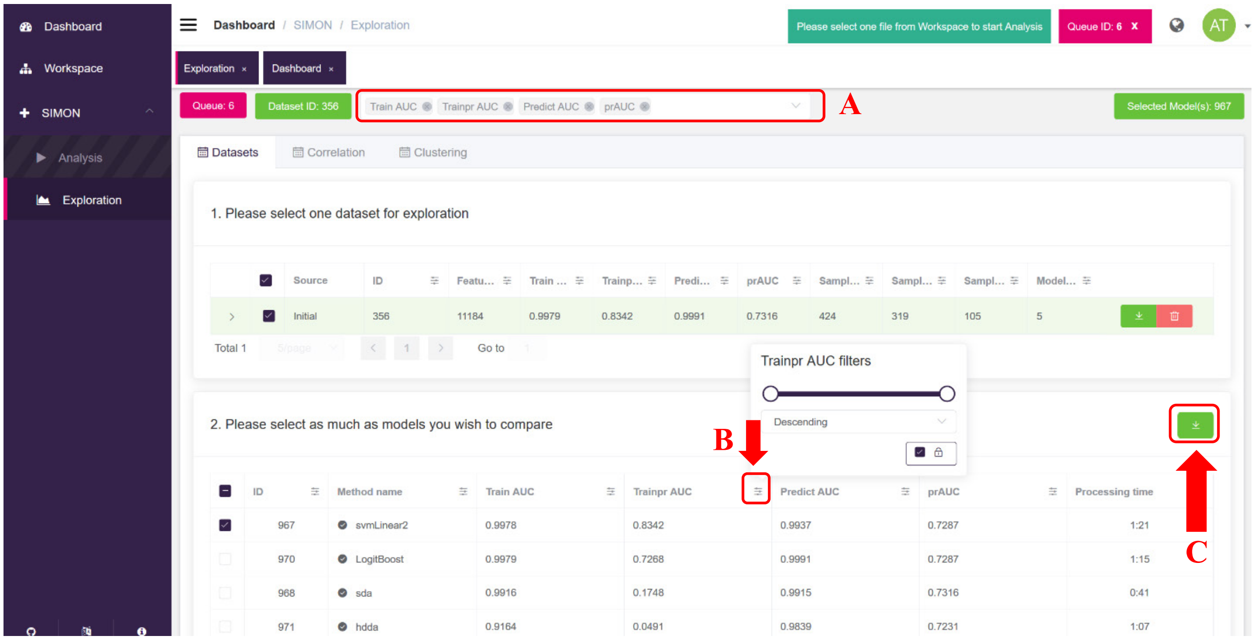

Supplemental figures

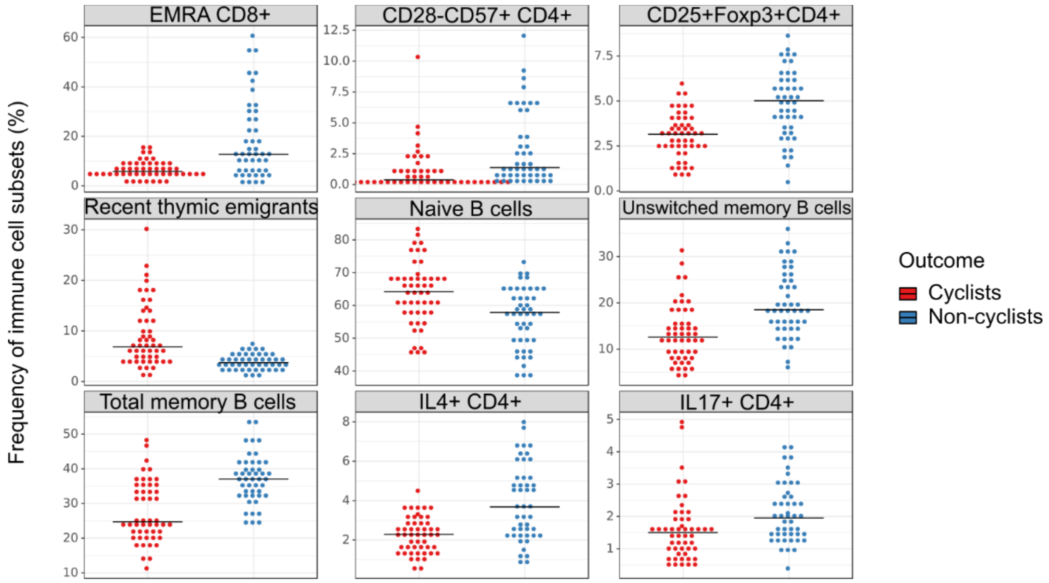

Figure S1. Frequency of immune cell subsets associated with high-level of physical activity. Dot plots represent distribution of immune cell subsets between cyclists (red dots) and non-cyclists (blue dots) as frequency (percentage of parent immune cell population) for the top nine selected features that contribute the most to the Cyclists model to discriminate between cyclists and non-cyclists. Each dot is one individual, lines indicate median values.

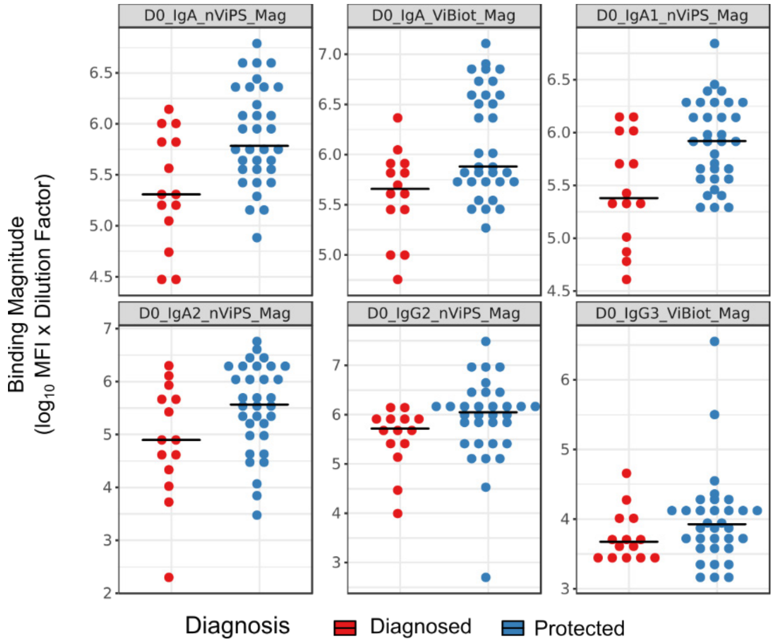

Figure S2. Antibody-mediated signature associated with the effective vaccine against *Salmonella* Typhi infection. Dot plots represent binding magnitude of indicated antibodies between diagnosed (red dots) and protected (blue dots) individuals. Each dot represents one individual, while lines indicate median values. The binding magnitude is log-transformed and given as Mean Fluorescence Intensity (MFI) multiplied by dilution factor. *D0*, day 0 (day of the challenge); *nViPS*, native Vi polysaccharide antigen; *ViBiot*, biotinylated Vi polysaccharide antigen; *Mag*, magnitude.

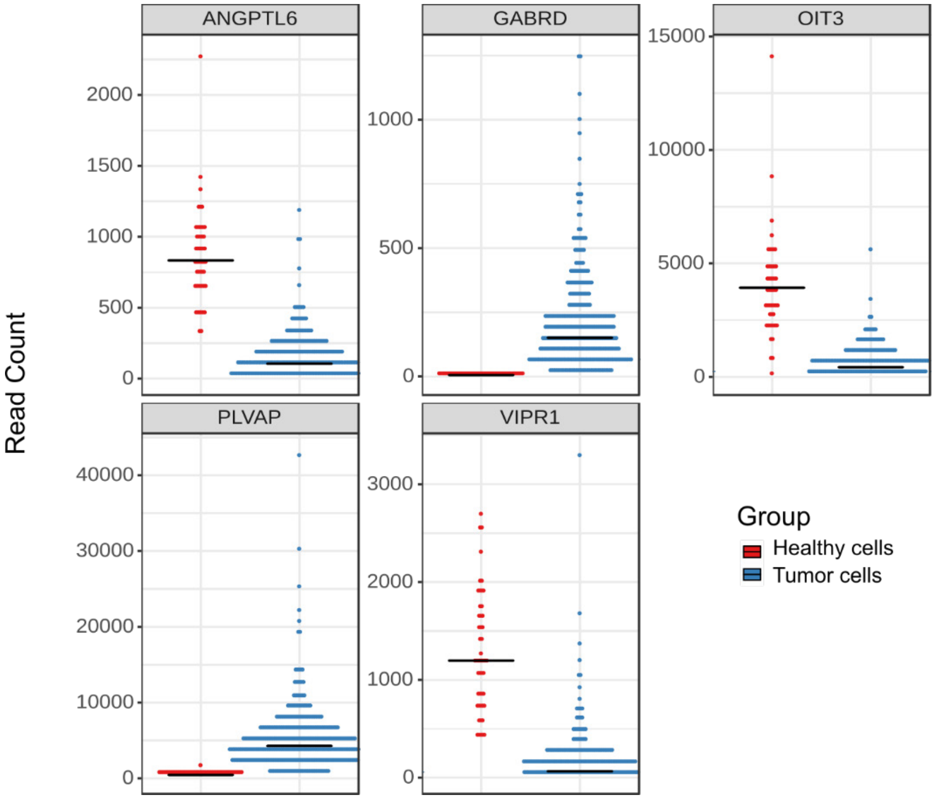

Figure S3. Gene expression signature specific for tumor cells. Dot plots represent read counts for top five genes that discriminate between healthy (red dots) and tumor (blue dots) cells selected in the top performing model. Each dot represents one sample, while lines indicate median values.

List of references of R packages used for Table S1:

1. Package: ada - Culp, M., Johnson, K. and Michailidis, G. (2016). ada: The R Package Ada for Stochastic Boosting. R package version 2.0-5. https://CRAN.R-project.org/package=ada
2. Package: plyr – Wickham, H. (2011). The Split-Apply-Combine Strategy for Data Analysis. Journal of Statistical Software 40(1), 1-29. URL http://www.jstatsoft.org/v40/i01/
3. Package: adabag – Alfaro, E., Gamez, M. and Garcia, N. (2013). adabag: An R Package for Classification with Boosting and Bagging. Journal of Statistical Software, 54(2), 1-35. URL http://www.jstatsoft.org/v54/i02/
4. Package: fastAdaboost – Chatterjee, S. (2016). fastAdaboost: a Fast Implementation of Adaboost. R package version 1.0.0. https://CRAN.R-project.org/package=fastAdaboost
5. Package: adaptDA – Bouveyron, C. (2014). adaptDA: Adaptive Mixture Discriminant Analysis. R package version 1.0. https://CRAN.R-project.org/package=adaptDA
6. Package: nnet - Venables, W. N. and Ripley, B. D. (2002) Modern Applied Statistics with S. Fourth Edition. Springer, New York. ISBN 0-387-95457-0
7. Package: bnclassify – Mihaljevic, B., Larranaga, P. and Bielza, C. (2018). bnclassify: Learning Bayesian Network Classifiers. The R Journal 10 (2): 455-468
8. Package: caret – Kuhn, M. (2020). caret: Classification and Regression Training. R package version 6.0-86. https://CRAN.R-project.org/package=caret
9. Package: earth – Milborrow, S. Derived from mda:mars by Trevor Hastie and Rob Tibshirani. Uses Alan Miller's Fortran utilities with Thomas Lumley's leaps wrapper. (2017). earth: Multivariate Adaptive Regression Splines. R package version 4.5.0. https://CRAN.R-project.org/package=earth
10. Package: mgcv - Wood, S.N. (2011) Fast stable restricted maximum likelihood \nand marginal likelihood estimation of semiparametric generalized linear \nmodels. Journal of the Royal Statistical Society (B) 73(1):3-36
11. Package: arm – Gelman, A. and Su, Y. (2018). arm: Data Analysis Using Regression and Multilevel/HierarchicalModels. R package version 1.10-1. https://CRAN.R-project.org/package=arm

12. Package: binda – Gibb, S. and Strimmer, K. (2015). binda: Multi-Class Discriminant Analysis using Binary Predictors. R package version 1.0.3. https://CRAN.R-project.org/package=binda
13. Package: party – Hothorn, T., Hornik, K.and Zeileis, A. (2006). Unbiased Recursive Partitioning: A Conditional Inference Framework. Journal of Computational and Graphical Statistics, 15(3), 651--674.
14. Package: partykit - Hothorn, T. and Zeileis, A. (2015). partykit: A Modular Toolkit for Recursive Partytioning in R. Journal of Machine Learning Research 16, 3905-3909. URL http://jmlr.org/papers/v16/hothorn15a.html
15. Package: bst – Wang, Z. (2019). bst: Gradient Boosting. R package version 0.3-17. https://CRAN.R-project.org/package=bst
16. Package: C50 – Kuhn, M. and Quinlan, R. (2020). C50: C5.0 Decision Trees and Rule-Based Models. R package version 0.1.3. https://CRAN.R-project.org/package=C50
17. Package: CHAID - The FoRt Student Project Team (2015). CHAID: CHi-squared Automated Interaction Detection R package version 0.1-2.
18. Package: rrcov – Todorov, V. and Filzmoser, P. (2009). An Object-Oriented Framework for Robust Multivariate Analysis. Journal of Statistical Software 32(3), 1-47. URL http://www.jstatsoft.org/v32/i03/
19. Package: rrcovHD – Todorov, V. (2019). rrcovHD: Robust Multivariate Methods for High Dimensional Data. R package version 0.2-6. https://CRAN.R-project.org/package=rrcovHD
20. Package: sparsediscrim – Ramey, J.A. (2017). sparsediscrim: Sparse and Regularized Discriminant Analysis. https://github.com/ramhiser/sparsediscrim; http://ramhiser.com
21. Package: deepboost – Marcous, D. and Sandbank, Y. (2017). deepboost: Deep Boosting Ensemble Modeling. R package version 0.1.6. https://CRAN.R-project.org/package=deepboost
22. Package: deepnet – Rong, X. (2014). deepnet: deep learning toolkit in R. R package version 0.2. https://CRAN.R-project.org/package=deepnet
23. Package: kerndwd - Wang, B. and Zou, H. (2018). kerndwd: Distance Weighted Discrimination (DWD) and Kernel Methods. R package version 2.0.2. https://CRAN.R-project.org/package=kerndwd

24. Package: kernlab - Karatzoglou, A., Smola, A., Hornik, K. and Zeileis, A. (2004). kernlab - An S4 Package for Kernel Methods in R. Journal of Statistical Software 11(9), 1-20. URL http://www.jstatsoft.org/v11/i09/
25. Package: earth – Milborrow, S.. Derived from mda:mars by Trevor Hastie and Rob Tibshirani. Uses Alan Miller's Fortran utilities with Thomas Lumley's leaps wrapper. (2017). earth: Multivariate Adaptive Regression Splines. R package version 4.5.0. https://CRAN.R-project.org/package=earth
26. Package: elmNN – Gosso, A. (2012). elmNN: Implementation of ELM (Extreme Learning Machine) algorithm for SLFN (Single Hidden Layer Feedforward Neural Networks). R package version 1.0. https://CRAN.R-project.org/package=elmNN
27. Package: evtree - Grubinger, T., Zeileis, A. and Pfeiffer, K. (2014). evtree: Evolutionary Learning of Globally Optimal Classification and Regression Trees in R. Journal of Statistical Software, 61(1), 1-29. URL http://www.jstatsoft.org/v61/i01
28. Package: caret – Kuhn, M. Contributions from Wing, J., Weston, S. Williams, A., Keefer, C., Engelhardt, A., Cooper, T., Mayer, Z., Kenkel, B., the R Core Team, Benesty, M., Lescarbeau, R., Ziem, A., Scrucca, L., Tang, Y., Candan, C. and Hunt, T. (2017). caret: Classification and Regression Training. R package version 6.0-76. https://CRAN.R-project.org/package=caret
29. Package: frbs - Riza, L.S., Bergmeir, C., Herrera, F. and Benitez, J.M. (2015). frbs: Fuzzy Rule-Based Systems for Classification and Regression in R. Journal of Statistical Software, 65(6), 1-30. URL http://www.jstatsoft.org/v65/i06/
30. Package: mgcv - Wood, S.N. (2011) Fast stable restricted maximum likelihood \nand marginal likelihood estimation of semiparametric generalized linear \nmodels. Journal of the Royal Statistical Society (B) 73(1):3-36
31. Package: mboost - Hothorn, T., Buehlmann, P., Kneib, T., Schmid, M. and Hofner, B.(2017). mboost: Model-Based Boosting, R package version 2.8-0, https://CRAN.R-project.org/package=mboost.
32. Package: gam – Hastie, T. (2017). gam: Generalized Additive Models. R package version 1.14-4. https://CRAN.R-project.org/package=gam
33. Package: h2o - LeDell, E., Gill, N., Aiello, S., Fu, A., Candel, A., Click, C., Kraljevic, T., Nykodym, T., Aboyoun, P., Kurka, M. and Malohlava, M. (2020). h2o: R Interface for the

'H2O' Scalable Machine Learning Platform. R package version 3.28.0.4. https://CRAN.R-project.org/package=h2o

34. Package: gbm - Greenwell, B., Boehmke, B., Cunningham, J. and GBM Developers (2019). gbm: Generalized Boosted Regression Models. R package version 2.1.5. https://CRAN.R-project.org/package=gbm

35. Package: glm2 – Marschner, I.C. (2011). Fitting generalized linear models with convergence problems. The R Journal, 3(2), 12-15. URL https://CRAN.R-project.org/package=glm2

36. Package: MASS - Venables, W. N. and Ripley, B. D. (2002) Modern Applied Statistics with S. Fourth Edition. Springer, New York. ISBN 0-387-95457-0

37. Package: gpls – Ding, B. (2018). gpls: Classification using generalized partial least squares. R package version 1.54.0.

38. Package: hda – Szepannek, G. (2016). hda: Heteroscedastic Discriminant Analysis. R package version 0.2-14. https://CRAN.R-project.org/package=hda

39. Package: HDclassif - Berge, L., Bouveyron, C. and Girard, S. (2012). HDclassif: An R Package for Model-Based Clustering and Discriminant Analysis of High-Dimensional Data. Journal of Statistical Software, 46(6), 1-29. URL http://www.jstatsoft.org/v46/i06/

40. Package: pls – Mevik, B., Wehrens, R. and Liland, K.H. (2019). pls: Partial Least Squares and Principal Component Regression. R package version 2.7-2. https://CRAN.R-project.org/package=pls

41. Package: kknn – Schliep, K. and Hechenbichler, K. (2016). kknn: Weighted k-Nearest Neighbors. R package version 1.3.1. https://CRAN.R-project.org/package=kknn

42. Package: klaR - Weihs, C., Ligges, U., Luebke, K. and Raabe, N. (2005). klaR Analyzing German Business Cycles. In Baier, D., Decker, R. and Schmidt-Thieme, L. (eds.). Data Analysis and Decision Support, 335-343, Springer-Verlag, Berlin.

43. Package: logicFS – Schwender, H. and Tietz, T. (2018). logicFS: Identification of SNP Interactions. R package version 2.2.0.

44. Package: caTools – Tuszynski, J. (2020). caTools: Tools: moving window statistics, GIF, Base64, ROC, AUC, etc. R package version 1.17.1.4. https://CRAN.R-project.org/package=caTools

45. Package: LogicReg – Kooperberg, C. and Ruczinski, I. (2019). LogicReg: Logic Regression. R package version 1.6.2. https://CRAN.R-project.org/package=LogicReg
46. Package: class - Venables, W. N. and Ripley, B. D. (2002) Modern Applied Statistics with S. Fourth Edition. Springer, New York. ISBN 0-387-95457-0
47. Package: mda - S original by Hastie, T. and Tibshirani, R. Original R port by Leisch, F., Hornik, K. and Ripley, B.D. (2017). mda: Mixture and Flexible Discriminant Analysis. R package version 0.4-10. https://CRAN.R-project.org/package=mda
48. Package: HiDimDA - Duarte Silva, A.P. (2015). HiDimDA: High Dimensional Discriminant Analysis. R package version 0.2-4. https://CRAN.R-project.org/package=HiDimDA
49. Package: RSNNS - Bergmeir, C. and Benitez, J.M. (2012). Neural Networks in R Using the Stuttgart Neural Network Simulator: RSNNS. Journal of Statistical Software, 46(7), 1-26. URL http://www.jstatsoft.org/v46/i07/
50. Package: keras – Allaire, J.J. and Chollet, F. (2019). keras: R Interface to 'Keras'. R package version 2.2.5.0. https://CRAN.R-project.org/package=keras
51. Package: FCNN4R – Klima, G. (2016), FCNN4R: Fast Compressed Neural Networks for R, R package version 0.6.2.
52. Package: monmlp – Cannon, A.J. (2017). monmlp: Monotone Multi-Layer Perceptron Neural Network. R package version 1.1.4. https://CRAN.R-project.org/package=monmlp
53. Package: nnet - Venables, W. N. and Ripley, B. D. (2002) Modern Applied Statistics with S. Fourth Edition. Springer, New York. ISBN 0-387-95457-0
54. Package: naivebayes – Majka, M. (2017). naivebayes: High Performance Implementation of the Naive Bayes Algorithm. R package version 0.9.1. https://CRAN.R-project.org/package=naivebayes
55. Package: nodeHarvest – Meinshausen, N. (2015). nodeHarvest: Node Harvest for Regression and Classification. R package version 0.7-3. https://CRAN.R-project.org/package=nodeHarvest
56. Package: ordinalNet - Wurm, M., Rathouz, P. and Hanlon, B. (2020). ordinalNet: Penalized Ordinal Regression. R package version 2.7. https://CRAN.R-project.org/package=ordinalNet

57. Package: e1071 - Meyer, D., Dimitriadou, E., Hornik, K., Weingessel, A. and Leisch, F. (2019). e1071: Misc Functions of the Department of Statistics Probability Theory Group (Formerly: E1071) TU Wien. R package version 1.7-3. https://CRAN.R-project.org/package=e1071
58. Package: ranger – Wright, M.N. and Ziegler, A. (2017). ranger: A Fast Implementation of Random Forests for High Dimensional Data in C++ and R. Journal of Statistical Software, 77(1), 1-17. doi:10.18637/jss.v077.i01
59. Package: dplyr - Wickham, H., François, R., Henry, L. and Müller, K. (2020). dplyr: A Grammar of Data Manipulation. R package version 0.8.5. https://CRAN.R-project.org/package=dplyr
60. Package: obliqueRF – Menze, B. and Splitthoff, N. (2012). obliqueRF: Oblique Random Forests from Recursive Linear Model Splits. R package version 0.3. https://CRAN.R-project.org/package=obliqueRF
61. Package: snn - Sun, W., Qiao, X. and Cheng, G. (2015). snn: Stabilized Nearest Neighbor Classifier. R package version 1.1. https://CRAN.R-project.org/package=snn
62. Package: pamr - Hastie, T., Tibshirani, R., Narasimhan, B. and Chu, G. (2019). pamr: Pam: Prediction Analysis for Microarrays. R package version 1.56.1. https://CRAN.R-project.org/package=pamr
63. Package: randomForest – Liaw, A. and Wiener, M. (2002). Classification and Regression by randomForest. R News 2(3), 18-22.
64. Package: foreach - Microsoft and Weston, S. (2020). foreach: Provides Foreach Looping Construct. R package version 1.5.0. https://CRAN.R-project.org/package=foreach
65. Package: partDSA – Molinaro, A.M., Lostritto, K. and van der Laan, M.J. (2010). partDSA: Deletion/Substitution/Addition Algorithm for Partitioning the Covariate Space in Prediction. Bioinformatics, 26(10), 1357-63.
66. Package: penalizedLDA – Witten, D. (2015). penalizedLDA: Penalized Classification using Fisher's Linear Discriminant. R package version 1.1. https://CRAN.R-project.org/package=penalizedLDA
67. Package: stepPlr – Park, M.Y. and Hastie, T. (2018). stepPlr: L2 Penalized Logistic Regression with Stepwise VariableSelection. R package version 0.93. https://CRAN.R-project.org/package=stepPlr

68. Package: proxy – Meyer, D. and Buchta, C. (2019). proxy: Distance and Similarity Measures. R package version 0.4-23. https://CRAN.R-project.org/package=proxy
69. Package: protoclass – Bien, J. and Tibshirani, R. (2013). protoclass: Interpretable classification with prototypes. R package version 1.0. https://CRAN.R-project.org/package=protoclass
70. Package: randomGLM – Song, L. and Langfelder, P. (2013). randomGLM: Random General Linear Model Prediction. R package version 1.02-1. https://CRAN.R-project.org/package=randomGLM
71. Package: rBorist – Seligman, M. (2019). Rborist: Extensible Parallelizable Implementation of the Random Forest Algorithm. R package version 0.2-3. https://CRAN.R-project.org/package=Rborist
72. Package: LiblineaR – Helleputte, T. (2017). LiblineaR: Linear Predictive Models Based On The Liblinear C/C++ Library.R package version 2.10-8.
73. Package: rFerns – Kursa, M.B. (2014). rFerns: An Implementation of the Random Ferns Method for General-Purpose Machine Learning. Journal of Statistical Software 61(10) 1-13. URL http://www.jstatsoft.org/v61/i10/
74. Package: robustDA – Bouveyron, C. and Girard, S. (2015). robustDA: Robust Mixture Discriminant Analysis. R package version 1.1. https://CRAN.R-project.org/package=robustDA
75. Package: rocc – Lauss, M. (2019). rocc: ROC Based Classification. R package version 1.3. https://CRAN.R-project.org/package=rocc
76. Package: rotationForest – Ballings, M. and Van den Poel, D. (2017). rotationForest: Fit and Deploy Rotation Forest Models. R package version 0.1.3. https://CRAN.R-project.org/package=rotationForest
77. Package: rpart – Therneau, T., Atkinson, B. and Ripley, B. (2017). rpart: Recursive Partitioning and Regression Trees. R package version 4.1-11. https://CRAN.R-project.org/package=rpart
78. Package: rpartScore - Galimberti, G., Soffritti, G. and Di Maso, M. (2012). Classification Trees for Ordinal Responses in R: The rpartScore Package. Journal of Statistical Software, 47(10), 1-25. URL http://www.jstatsoft.org/v47/i10/

79. Package: RRF – Deng, H. (2013). Guided Random Forest in the RRF Package. arXiv:1306.0237.
80. Package: sda - Ahdesmaki, M., Zuber, V., Gibb, S. and Strimmer, K. (2015). sda: Shrinkage Discriminant Analysis and CAT Score Variable Selection. R package version 1.3.7. https://CRAN.R-project.org/package=sda
81. Package: sdwd – Wang, B. and Zou, H. (2020). sdwd: Sparse Distance Weighted Discrimination. R package version 1.0.3. https://CRAN.R-project.org/package=sdwd
82. Package: ipred – Peters, A. and Hothorn, T. (2019). ipred: Improved Predictors. R package version 0.9-9. https://CRAN.R-project.org/package=ipred
83. Package: sparseLDA – Clemmensen, L. and contributions by Kuhn, M. (2016). sparseLDA: Sparse Discriminant Analysis. R package version 0.1-9. https://CRAN.R-project.org/package=sparseLDA
84. Package: spls - Chung, D., Chun, H. and Keles, S. (2019). spls: Sparse Partial Least Squares (SPLS) Regression and Classification. R package version 2.2-3. https://CRAN.R-project.org/package=spls
85. Package: vbmp – Lama, N. and Girolami, M. (2018). vbmp: Variational Bayesian Multinomial Probit Regression. R package version 1.50.0. http://bioinformatics.oxfordjournals.org/cgi/content/short/btm535v1
86. Package: VGAM – Yee, T.W. (2015). Vector Generalized Linear and Additive Models: With an Implementation in R. New York, USA: Springer.
87. Package: wsrf - Zhao, H., Williams, G.J. and Huang, J.Z. (2017). wsrf: An R Package for Classification with Scalable Weighted Subspace Random Forests. Journal of Statistical Software, 77(3), 1-30. doi:10.18637/jss.v077.i03
88. Package: xgboost - Chen, T., He, T., Benesty, M., Khotilovich, V., Tang, Y., Cho, H., Chen, K., Mitchell, R., Cano, I., Zhou, T., Li, M., Xie, J., Lin, M., Geng, Y. and Li, Y. (2020). xgboost: Extreme Gradient Boosting. R package version 1.0.0.2. https://CRAN.R-project.org/package=xgboost
