# Supplementary material for: SIMON: Open-Source Knowledge Discovery Platform
Source: Patterns (N Y). 2021 Jan 8;2(1):100178. doi: 10.1016/j.patter.2020.100178 (PMC7815964; doi:10.1016/j.patter.2020.100178)
Supplement: Document S2. Article plus Supplemental Information [file mmc28.pdf]

Descriptor

Patterns

SIMON: Open-Source Knowledge Discovery Platform

Graphical Abstract

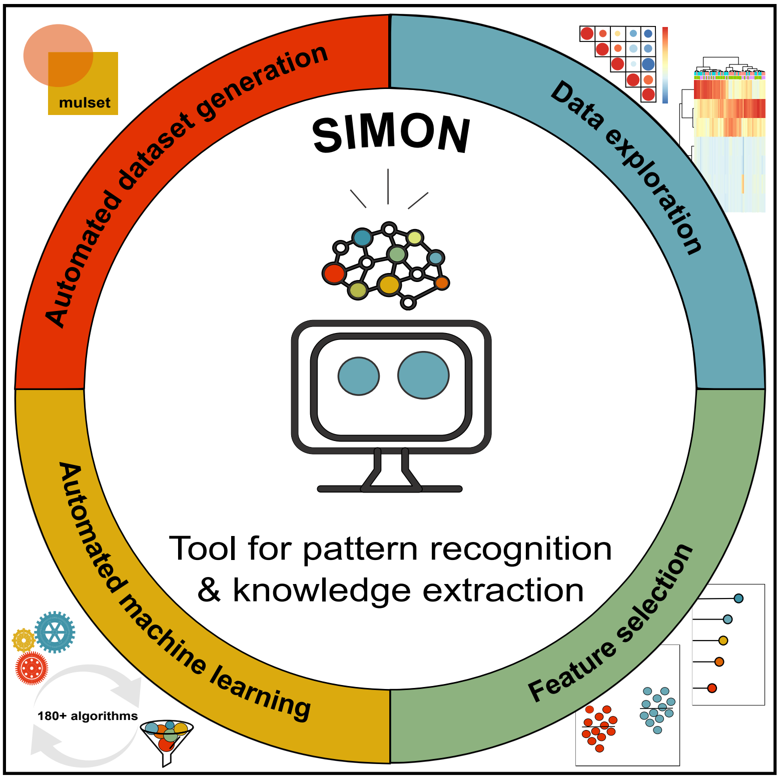

Highlights

- SIMON is an open-source software for analysis of high-dimensional biomedical data
- SIMON facilitates application of 180+ machine learning algorithms
- Easy-to-use graphical use interface and no programming expertise required
- SIMON empowers biomedical researchers to identify patterns in biomedical data

Authors

Adriana Tomic, Ivan Tomic, Levi Waldron, ..., Purvesh Khatri, Andrew J. Pollard, Mark M. Davis

Correspondence

info@adrianatomic.com (A.T.),
info@ivantomic.com (I.T.),
mmdavis@stanford.edu (M.M.D.)

In Brief

Tomic et al. developed SIMON, an open-source software for application of machine learning algorithms to high-dimensional biomedical data ranging from the transcriptome to flow cytometry to the microbiome. Using a graphical user interface, standardized pipelines for predictive modeling, and automated machine learning, SIMON empowers non-technical biomedical researchers to identify patterns in their data and build high-quality predictive models.

Tomic et al., 2021, Patterns 2, 100178
January 8, 2021 © 2020 The Author(s).
https://doi.org/10.1016/j.patter.2020.100178

CellPress

Patterns

CellPress
OPEN ACCESS

Descriptor

SIMON: Open-Source Knowledge Discovery Platform

Adriana Tomic,[1,2,15,16,*] Ivan Tomic,[3,15,*] Levi Waldron,[4,5] Ludwig Geistlinger,[4,5] Max Kuhn,[6] Rachel L. Spreng,[7] Lindsay C. Dahora,[7] Kelly E. Seaton,[7] Georgia Tomaras,[7] Jennifer Hill,[1] Niharika A. Duggal,[8] Ross D. Pollock,[9] Norman R. Lazarus,[9] Stephen D.R. Harridge,[9] Janet M. Lord,[8,10] Purvesh Khatri,[2,11] Andrew J. Pollard,[1,14] and Mark M. Davis[2,12,13,14,*]

[1]Oxford Vaccine Group, Department of Paediatrics, University of Oxford, Oxford, UK
[2]Institute of Immunity, Transplantation, and Infection, Stanford University School of Medicine, Stanford, CA, USA
[3]Deep Medicine, Nuffield Department of Women's and Reproductive Health, University of Oxford, Oxford, UK
[4]Graduate School of Public Health and Health Policy, City University of New York, New York, NY, USA
[5]Institute for Implementation Science and Population Health, City University of New York, New York, NY, USA
[6]RStudio, PBC, Boston, MA, USA
[7]Duke Human Vaccine Institute, Duke University, Durham, NC, USA
[8]MRC-Versus Arthritis Centre for Musculoskeletal Ageing Research, Institute of Inflammation and Ageing, University of Birmingham Research Labs, Birmingham, UK
[9]Centre for Human and Applied Physiological Sciences, King's College London, UK
[10]NIHR Birmingham Biomedical Research Centre, University Hospital Birmingham NHS Foundation Trust and University of Birmingham, Birmingham, UK
[11]Center for Biomedical Informatics Research, Department of Medicine, Stanford University, Stanford, CA, USA
[12]Department of Microbiology and Immunology, Stanford University School of Medicine, Stanford, CA, USA
[13]Howard Hughes Medical Institute, Stanford University, Stanford, CA, USA
[14]Senior author
[15]These authors contributed equally
[16]Lead Contact
*Correspondence: info@adrianatomic.com (A.T.), info@ivantomic.com (I.T.), mmdavis@stanford.edu (M.M.D.)
https://doi.org/10.1016/j.patter.2020.100178

THE BIGGER PICTURE Over the past years, technological advances have enabled the generation of large amounts of data at multiple scales. The integration of high-dimensional data is particularly important in biomedical sciences, as they can be used to identify biological mechanisms and predict clinical outcomes well in advance of their occurrence. Because of the lack of powerful analytical tools that can be used by the average biomedical researcher, translation of such knowledge has been extremely slow. We have developed an open-source software, SIMON, to facilitate the application of machine learning to high-dimensional biomedical data. In SIMON, analysis is performed using an intuitive graphical user interface and standardized, automated machine learning approach allowing non-technical researchers to identify patterns and extract knowledge from high-dimensional data and build high-quality predictive models.

1 2 3 4 5 **Development/Pre-production:** Data science output has been rolled out/validated across multiple domains/problem

SUMMARY

Data analysis and knowledge discovery has become more and more important in biology and medicine with the increasing complexity of biological datasets, but the necessarily sophisticated programming skills and in-depth understanding of algorithms needed pose barriers to most biologists and clinicians to perform such research. We have developed a modular open-source software, SIMON, to facilitate the application of 180+ state-of-the-art machine-learning algorithms to high-dimensional biomedical data. With an easy-to-use graphical user interface, standardized pipelines, and automated approach for machine learning and other statistical analysis methods, SIMON helps to identify optimal algorithms and provides a resource that empowers non-technical and technical researchers to identify crucial patterns in biomedical data.

This is an open access article under the CC BY license (http://creativecommons.org/licenses/by/4.0/).

INTRODUCTION

Over the past several years, due to the technological breakthroughs in genome sequencing,[1] high-dimensional flow cytometry,[2–4] mass cytometry,[5,6] and multiparameter microscopy,[7,8] the amount and complexity of biological data have become increasingly intractable and it is no longer feasible to extract knowledge without using sophisticated computer algorithms. Therefore, researchers are in need of novel computational approaches that can cope with the complexity and heterogeneity of data in an objective and unbiased way. Machine learning (ML), a subset of artificial intelligence, is a computational approach developed to identify patterns from the data in order to make predictions on new data.[9] ML has had a profound impact on biological research,[10–12] including genomics,[13] proteomics,[14–16] cell image analysis,[17] drug discovery and development,[18] and cell phenotyping,[6,19,20] which revolutionized our understanding of biological complexity. Recently, using systems-level analysis of genetic, transcriptional, and proteomic signatures to predict patients' response to vaccines,[21,22] therapies, and disease progression,[23–27] ML has become the primary computational approach used in "precision medicine."[28]

The biggest challenge is the proper application of ML methods and the translation of the results into meaningful insights. The analysis of massive datasets and extraction of knowledge using ML require knowledge of many different computational libraries for data pre-processing and cleaning, data partitioning, model building and tuning, evaluation of the performance of the model, and minimizing overfitting.[11] Tools to achieve these tasks have been mainly developed in either R (https://www.r-project.org/)[29,30] or Python (www.python.org/),[31] which have today become leading statistical programming languages in data science. Because R and Python are free and open source, they have been quickly adopted by a large community of programmers who are building new libraries and improving existing ones. As of May 2020, there are 15,658 R packages available in the CRAN package repository (https://cran.r-project.org/). Many of the packages offer different modeling functions and have different syntaxes for model training, predictions, and determination of variable importance. Due to the lack of a unified method for proper application of ML processes, even experienced bioinformaticians struggle with these time-consuming ML tasks. To provide a uniform interface and standardize the process of building predictive models, ML libraries were developed, for example, mlr3[32] (https://mlr3.mlr-org.com), classification and regression training (caret)[30,33] (https://rdrr.io/cran/caret), scikit-learn[34] (https://scikit-learn.org), mlPy[35] (https://mlpy.fbk.eu), and SciPy (https://www.scipy.org/), including also ones for deep learning, such as TensorFlow (https://www.tensorflow.org/), PyTorch (https://pytorch.org/), and Keras (https://keras.io/). Since those libraries do not have a graphical user interface, usage requires extensive programming experience and general knowledge of R or Python, making them inaccessible for many life science researchers. Therefore, there is an increased effort to harmonize those libraries and develop a software that will facilitate application of ML in life sciences.

The software should provide a standardized ML method for data pre-processing, data partitioning, building predictive models, evaluation of model performance, and selection of features. Moreover, such software should be adapted for biological datasets that have a high percentage of missing values,[36] have unbalanced participant distributions (i.e., a high number of infected subjects, but only a relatively small number of healthy controls),[37] or suffer from a "curse of dimensionality," i.e., poor predictive power, as can be observed when the number of features is much greater than the number of samples.[38] In addition, beyond the ML process, the software should support exploratory analysis and visualization of the results using a user-friendly graphical interface. The fast-paced technological development has dramatically increased the size of biological datasets and the computational power needed for analysis. Therefore, open-source web-based software supporting cloud processing architecture is essential.

RESULTS

To address these challenges, we developed SIMON (Sequential Iterative Modeling "Over Night"), a free and open-source software for application of ML in life sciences that facilitates production of high-performing ML models and allows researchers to focus on the knowledge discovery process. SIMON provides a user-friendly, uniform interface for building and evaluating predictive models using a variety of ML algorithms. Currently, there are 182 different ML algorithms available (Table S1). The entire ML process, which is based on the caret[33] library, from model building and evaluation to feature selection, is fully automated, as described.[39] This allows advanced ML users to focus on other important aspects necessary to build highly accurate models, such as data pre-processing, feature engineering, and model deployment. It also makes the entire ML process more accessible to domain-knowledge experts who formulate the research hypothesis and collect the data, but lack programming ML skills. In addition, to prevent optimistic accuracy estimates and to optimize the model for generalization to unseen data, SIMON introduces a unified process for model training, hyperparameter tuning, and model evaluation by generation of training, validation, and test sets. A training set is used for building models, which are evaluated using 10-fold cross-validation; a validation set is used for hyperparameter tuning, and finally, models are evaluated in an unbiased way using a test set, also known as a holdout set, that has never been used for training. Models can be downloaded as Rdata formats, which is crucial for usability and reproducibility. In addition to the standardized ML process, the initial install version offers a set of core components specifically suited to the analysis of biomedical data, such as a multiset intersection function for integration of data with many missing values (https://cran.r-project.org/web/packages/mulset/index.html), a method for identifying differentially expressed genes using significance analysis in microarrays,[40] a graphical representation of the clustering analysis important for detection of batch effects, a graphical display of the correlation analysis, and graphical visualizations of the ML results that can be downloaded as publication-ready figures in scalable vector graphics format. Finally, SIMON is available in two versions as a single mode and a server version. The single mode is developed as a SIMON Docker container (https://www.docker.com/), ensuring code reproducibility and solving installation compatibility issues across major operating systems (Windows, MacOS, and Linux).

CellPress
OPEN ACCESS

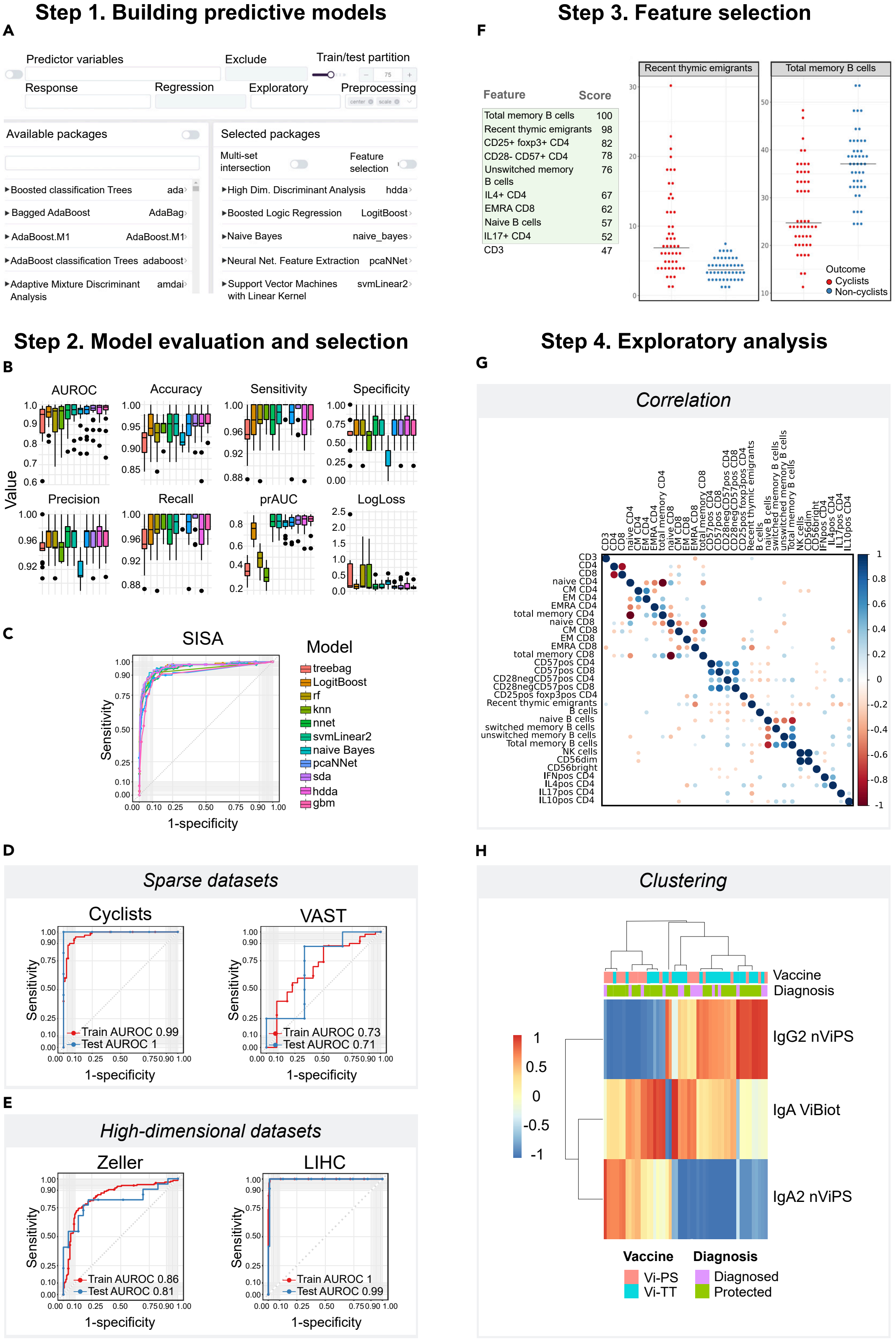

(legend on next page)

In both versions parallel computing is supported, which is essential for more efficient ML analysis by distributing the workload across several processors. To promote collaboration and data sharing and support distributed cloud processing, SIMON is also available as a server version. The server version can be installed on a private or public Linux cloud service. Distributed cloud processing (multiNode) is implemented utilizing OpenStack, a free and open-source cloud computing platform (https://www.openstack.org/). The advantage of the server version is that it has multiNode capability, which allows users to distribute workload on multiple computers simultaneously to optimize SIMON performance. The multiNode process can be used to horizontally scale analysis to large infrastructures, such as high-performance computing clusters to meet the computational needs and accommodate parallel processing of large amounts of data. In addition, in the server version, users can configure data storage either on a local server or in a cloud-using service that is interoperable with the Amazon Web Services S3 application programming interface.[41] SIMON has also been translated into multiple languages by a collaborative open-source effort. SIMON source code is regularly updated, and both source code and compiled software are available from the project's website at http://www.genular.org/.

We demonstrate the accuracy, ease of use, and power of SIMON on five different biomedical datasets and build predictive models for arboviral infection severity (SISA),[42] the identification of the cellular immune signature associated with a high-level of physical activity (Cyclists),[43] the determination of the humoral responses that mediate protection against *Salmonella* Typhi infection (VAST),[44] early stage detection of colorectal cancer from microbiome data (Zeller),[45,46] and the detection of liver hepatocellular carcinoma cells (LIHC)[47] (Figure 1B–1E; Supplemental Information, Videos S1 and S6). To build models using the SISA dataset containing clinical parameters (described in the Experimental Procedures and available as Table S2), 11 ML algorithms were used, 5 from the original publication[42] (treebag, k nearest neighbors, random forest, stochastic generalized boosting model, and neural network) and, in addition, "sda," shrinkage discriminant analysis; "hdda," high-dimensional discriminant analysis; "svmLinear2," support vector machine with linear kernel; "pcaNNet," neural networks with feature extraction; "LogitBoost," boosted logistic regression, and naive Bayes. Due to the unified ML process for training, tuning, and evaluating predictive models, users can test a variety of ML algorithms in SIMON. Since the same training and test sets are used by different algorithms, resulting models can be compared and the best-performing models can be selected. After manually setting initial parameters for data partitioning, predictor and outcome variables, exploratory classes, pre-processing, and selecting ML algorithms (Figure 1A), SIMON automatically performs all necessary ML analysis steps to build, tune, and evaluate predictive models. The process of building all 11 models on the SISA dataset in SIMON finished in 59 s on a standard laptop (Intel Core i7 Processor 7700HQ and 16 GB of RAM). In SIMON, users can evaluate model performance using standard performance measurements such as accuracy, sensitivity, specificity, precision, recall, area under the receiver operating characteristic curve (AUROC), precision-recall area under curve (prAUC), and logarithmic loss (LogLoss) on training and holdout test sets (Figure 1B; Videos S2 and S3). The shrinkage discriminant analysis model (sda) had the highest AUROC of 0.97 on the training set and also performed well on the holdout test set (test AUROC 0.96) (Figure 1C, Table S3, the model is available as the Data S1).

To demonstrate SIMON's capabilities for analyzing biomedical datasets with missing data, we applied SIMON to (1) the Cyclists dataset studying the impact of physical activity on the immune system in adulthood based on immunophenotyping using flow cytometry[43] (Table S4) and (2) the VAST dataset containing serological analysis of the antibody responses collected from a clinical trial that was undertaken to evaluate typhoid vaccine efficacy[48] (Table S5). Description of both datasets is available in the Experimental Procedures. The percentage of missing values was 8% in the Cyclists dataset and 21% in the VAST dataset, due to either the exclusion of samples not passing quality control criteria or the lack of sample volume to repeat experiments and obtain reportable data. To build models using the datasets with missing values, we used the multiset intersection ("mulset") function[39] to identify shared features between donors and generate resamples (Supplemental Information). Because the mulset function generates multiple resamples from the initial dataset based on shared features, it is useful for removal of missing values and can be used for integration of data collected from different assays and across clinical studies.[39] For the Cyclists dataset, the mulset function generated 146 resamples. The models were built for each of the 146 resamples using five ML algorithms (naive Bayes, svmLinear2, pcaNNet, logistic regression, and hdda) to identify immune cell subsets enriched in the cohort of master cyclists. The analysis finished in 41 min and 24 s. The model with the highest performance measures was built with naive Bayes on the resample with 96 donors that shared 31 features (train AUROC 0.99 and test AUROC 1) (Figure 1D, Table S6, and Data S2). The mulset function generated 206 resamples from the initial VAST dataset with varying number

Figure 1. SIMON Machine Learning Workflow

Step 1. Building predictive models. (A) Screenshot of the SIMON graphical user interface demonstrating input selection for machine learning analysis, such as predictors and response (outcome) variables, additional exploration classes, training/test split, pre-processing functions, and desired machine learning algorithms.

Step 2. Model evaluation and selection. Comparison of (B) box plots of performance measurements calculated for 11 predictive models and (C) receiver operating characteristic (ROC) curves built on the SISA dataset. Each boxplot shows the distribution of data as minimum (Q1−1.5×IQR), first quartile (Q1), median (Q2), third quartile (Q3), and maximum (Q3+1.5×IQR). Data outside of minimum and maximum values (outliers) are shown as circles. IQR, interquartile range.-Comparison of ROC curves calculated from the training (average value calculated using 10-fold cross-validation repeated three times) and test sets on (D) datasets with missing values (Cyclists and VAST) and (E) high-dimensional datasets (Zeller and LIHC).

Step 3. Feature selection. (F) The variable importance score table for each feature and graphical visualization of the selected features from the Cyclists dataset.

Step 4. Exploratory analysis. (G) Correlation analysis on the Cyclists dataset. (H) Clustering analysis on the VAST dataset.

of donors and features. Resamples with fewer than 10 donors in the test set were removed prior to the ML process to prevent too optimistic predictive estimates using the holdout set. Therefore, the ML analysis was performed on 58 resamples using the same five ML algorithms as for the Cyclists dataset. The entire analysis finished in 31 min and 1 s. The top performing model was built on the resample with 47 donors that shared 13 features with the naive Bayes algorithm (train AUROC 0.73 and test AUROC 0.71) (Figure 1D, Table S7, and Data S3).

We also applied SIMON to (1) a dataset with a large number of features measured using whole-metagenome shotgun sequencing of fecal samples (Zeller dataset, Table S8) and (2) the liver hepatocellular carcinoma dataset containing RNA-sequencing data from The Cancer Genome Atlas (TCGA) with an unbalanced sample distribution of tumor and adjacent normal tissue samples (LIHC dataset, Table S9). Both datasets are described in the Experimental Procedures. For the Zeller dataset, models were built using ML algorithms known to perform well in situations where more features were measured than individuals, such as shrinkage discriminant analysis,[49] high-dimensional discriminant analysis,[50] and neural network with feature extraction.[51] Two additional algorithms were included, svmLinear2 and LogitBoost. The complete analysis was performed in less than 1 min (0:38 min). The sda algorithm built the model with the highest performance (train AUROC 0.86 and test AUROC 0.81), having a higher performance measure than the published LASSO linear regression model[45] (train AUROC 0.84 and test AUROC 0.85) (Figure 1E, Table S10, and Data S4). For the LIHC dataset we used the same five ML algorithms as for the Zeller dataset, and analysis finished in 11 min and 30 s. For such a highly unbalanced dataset the precision-recall AUC (prAUC)[52] is a much better performance measurement than AUROC that reported near-perfect performance (Figure 1E). The prAUC provides information on how well the model correctly detects cancer cells, while it is less stringent on the evaluation of healthy cells. To avoid obtaining overly optimistic prediction results (often observed on unbalanced datasets), we ranked models based on the prAUC of the training set (Table S11). The model that had the best performance was built using the svmLinear2 algorithm (train prAUC 0.83) and it also performed well on the holdout test set (prAUC 0.73) (Data S5).

"Drowsiness" contributed the most to the top-performing SISA model, confirming the findings from the original study[42] (Table S12). To standardize the process for evaluation of the features and their contribution to the models, we implemented the variable importance score evaluation functions from the caret library.[33] This allows users to compare features selected across models. In the case of the SISA dataset, drowsiness contributed the most in all of the models built (Table S13), indicating the importance of this symptom and its correlation with hospitalization. The features that contributed the most to the Cyclists model were total memory, unswitched memory, and naive B cells; recent thymic emigrants; $CD8^+$ T cells with TEMRA phenotype; and regulatory T cells ($CD25^+$ $Foxp3^+$ $CD4^+$ T cells) (Table S14; Video S4). In comparison to age-matched physically inactive individuals (non-cyclists), the master cyclists had increased frequencies of recent thymic emigrants, naive B cells, and CD3 cells, and decreased frequencies of memory B cells and CD8 T cells with TEMRA phenotype, confirming that aging of the immune system, i.e., immunosenescence, can be reduced by high levels of physical activity[43] (Figures 1F and S1). To further explore the relationship between selected features, users can perform correlation analysis to reveal highly correlated features (Figure 1G; Supplemental Information, Video S5). Naive and memory B cells were identified as being highly correlated (Figure 1G), as expected, since these subsets were determined from the same flow cytometry plots and their relationship is inversely correlated. Removal of those highly correlated features can help to build more accurate models. Removal of naive B cells resulted in building a predictive model with the same performance measurements as the model built on the entire dataset (train AUROC 0.99 and test AUROC 1) (Table S15), while removal of total memory B cells lowered the accuracy estimates (train AUROC 0.98 and test AUROC 1) (Table S16), indicating the importance of memory B cells to discriminate between master cyclists and non-cyclists. In the VAST dataset, individuals with higher IgA, IgA1, IgA2, and IgG2 titers against native Vi polysaccharide (nViPS) antigen and higher IgA and IgG3 titers against biotinylated Vi polysaccharide (ViBiot) on the day of the challenge were protected against the typhoid challenge, supporting the data from univariate analysis[44] (Table S17 and Figure S2). Moreover, using the clustering function of SIMON's exploratory analysis module, we quickly found that the IgA2 signature dominates the responses after vaccination with a purified Vi polysaccharide (Vi-PS), while the IgG2 signature was dominant for the Vi tetanus toxoid conjugate (Vi-TT) vaccine[44] (Figure 1H, Supplemental Information). For the Zeller dataset, the same features as originally reported[45] contributed the most to the model, including *Fusobacterium nucleatum* and *Peptostreptococcus stomatis* (Table S18). The features that contributed the most to the LIHC model were well-known genes identified to be upregulated in LIHC, such as *GABRD* and *PLVAP*,[53] and genes enriched in adjacent normal tissue samples, *ANGPTL6*,[54] *VIPR1*,[55] and *OIT3*,[56] as a typical signature for healthy liver tissue (Table S19, Figure S3).

DISCUSSION

We have developed SIMON, a powerful software platform for data mining that facilitates pattern recognition and knowledge extraction from high-quality, heterogeneous biological and clinical data, especially where there are missing data, an unbalanced distribution, and/or high dimensionality. It can be used for the identification of genetic, microbial, and immunological correlates of protection and it can help in guiding the further analysis of the biomedical data.

Over the past years, technological advances have enabled the generation of large amounts of data at multiple scales. Monitoring and analyzing these complex datasets is particularly important in the biomedical sciences, as they serve to advance knowledge about health and disease, as well as predicting clinical outcomes in advance of their occurrence. Despite major clinical and economic consequences of these approaches, due to the lack of powerful analytical tools that can be used by the average biomedical researcher, the translation of such knowledge can be extremely slow. Although several commercial softwares are available, for instance, Google's cloud-based AutoML

(https://cloud.google.com/automl), DataRobot (https://www.datarobot.com/), BigML (https://bigml.com/), MLjar (https://mljar.com), and RapidMiner (https://rapidminer.com/), they come at a high price and have hidden ML methods and algorithms, and thus have not been adopted by the biomedical community. In academia, open-source ML software is being developed, for example, Waikato Environment for Knowledge Analysis (WEKA)[57] (https://www.cs.waikato.ac.nz/~ml/weka/), Orange[58] (https://orange.biolab.si/), the Konstanz Information Miner (KNIME)[59] (https://www.knime.com/), and ELKI[60] (https://elki-project.github.io/). For the development of SIMON, our aim was to integrate the capabilities of commercial software openly and freely for everyone. The currently available open-source software offers only a limited number of the most commonly used ML algorithms using a user-friendly graphical interface, while focusing on the manual configuration to achieve optimal model predictive performance. Therefore, usage of these softwares requires extensive knowledge of the ML process, so the primary users are data scientists, statisticians, and ML experts. In contrast, commercial software versions are implementing an automated ML (autoML) process that rapidly builds high-performance models by identifying the optimal ML method, including the selection of an appropriate algorithm, optimization of model hyperparameters, and evaluation of the best-performing models.[61] AutoML improves the efficiency of the ML process, and the resulting models often outperform hand-designed ones.[61,62] By implementing this simplified application of ML in SIMON, non-experts can build high-performing models. In addition to auto-Weka, which provides a graphical user interface for an open-source version of the autoML[63] constrained to the most commonly used ML algorithms, there are also frameworks, such as auto-sklearn,[64] TPOT,[65] and Auto-Prognosis,[66] highlighting the importance of the application of autoML to biomedical data. Although the process of selection of algorithms and optimization of hyperparameters is automated in SIMON, the data pre-processing steps and exploratory analysis of the resulting models require background knowledge about the data distribution and correlation, transformations, and processing steps before running the analysis and evaluation of predictive models built by SIMON.

Another advantage of commercial over open-source software, which we implemented in SIMON, is the architecture of commercial software supports running ML processes in the cloud or in the server mode. The SIMON server edition provides an option for web-based collaborative efforts that reflect the necessity to accommodate the increased size of datasets, the complexity of models, and data privacy concerns, for instance, for sharing human genomic data. Because the integration of biomedical data across clinical studies and research groups around the globe can enable training of more detailed models and lead to higher-quality insights, the SIMON server mode offered as an open-source version of ML software is a valuable tool.

SIMON is developed as a modular open-source software, which allows us to extend our work by integrating novel features in the future versions. Although this version of the software can analyze multiple datasets, ranging from clinical and cytometry data to transcriptome, microbiome, and proteome with missing data, high dimensionality, and unbalanced distributions, future multi-omics datasets integrating different modalities or time-series datasets will require new methods, such as ensemble methods, automated feature selection,[67] and forecasting algorithms. Moreover, as the number of predictive models built using biomedical data increases, SIMON will be able to identify which algorithms work the best for a particular dataset.

Overall, SIMON is designed to provide a uniform knowledge discovery interface adaptable to the increasing size of biomedical datasets that can allow even non-expert biomedical researchers to solve important problems when faced with complex and heterogeneous datasets.

EXPERIMENTAL PROCEDURES

Resource Availability

Lead Contact

The lead contact for this article is Adriana Tomic (info@adrianatomic.com).

Materials Availability

Datasets used in Figure 1 were either obtained directly from authors (VAST[44] and Cyclists[43] datasets) or downloaded from publications[42] (SISA dataset) and R packages (Zeller dataset from the MetagenomicData[46] and LIHC from the GSEABenchmarkeR[47]) with help from the authors. The SISA dataset contains data from 543 individuals hospitalized due to arboviral infection with dengue, chikungunya, or Zika virus from a surveillance study in Ecuador collected from 2013 to 2017. In the SISA dataset we excluded columns with high level of missing values (pregnancy, "WomPreg," and complete blood count test, which was not performed for all donors and includes the columns "PLT_count," "Lymphocytes," "CBC_N%," "WBC_calc," and "CBC_HCT"). In addition, nine donors with missing values were removed. The final SISA dataset after removal of columns and rows with missing values is available as Table S2. The Cyclists dataset contains data from the immune responses of 120 elderly individuals with a high-level of physical activity, i.e., master cyclists, and 75 age-matched controls with a low level of physical activity (non-cyclists) analyzed using flow cytometry (Table S4). The VAST dataset contains data from 72 individuals enrolled in the clinical study to evaluate humoral responses in a typhoid vaccine efficacy trial in a controlled human infection model. Only day 0 (day of the challenge) log-transformed data were used and are available for download as Table S5. Individuals were vaccinated with either a purified Vi-PS vaccine (35 individuals) or the Vi-TT vaccine (37 individuals) 1 month prior to oral challenge with live *Salmonella* Typhi. Of 72 individuals, 26 developed an acute typhoid infection following challenge. The Zeller dataset contains information on the microbiome species abundance in healthy individuals and colorectal cancer patients (Table S8). The data were accessed through the MetagenomicData package. In total 184 individuals were included, of which 93 were healthy controls and 91 colorectal cancer patients. The LIHC dataset obtained from the GSEABenchmarkeR package contains RNA expression data from 374 LIHC cells and 50 adjacent normal cells (Table S9).

Data and Code Availability

The source code of SIMON is available at https://github.com/genular/simon-frontend. All data used in SIMON analysis are available as Supplemental tables, while ML models are available as Supplemental data in the RData format. Datasets are available for the download from the Zenodo data repository: VAST (Zenodo Data: http://doi.org/10.5281/zenodo.4121322),[68] SISA Zenodo Data: http://doi.org/10.5281/zenodo.4121831),[69] Cyclists (Zenodo Data: http://doi.org/10.5281/zenodo.4115626),[70] Zeller (Zenodo Data: http://doi.org/10.5281/zenodo.4121516),[71] and LIHC (Zenodo Data: http://doi.org/10.5281/zenodo.4121594).[72]

Installing SIMON

SIMON can be installed directly from the GitHub (https://github.com/genular/simon-frontend) or a pre-built version can be installed from DockerHub (https://www.docker.com/). Users need to install Docker (version 17.05 or later required) following instructions available on the Docker website (https://docs.docker.com/). Installation instructions for Windows (https://docs.docker.com/docker-for-windows/install/), MacOS (https://docs.docker.com/docker-for-mac/install/), and Linux (https://docs.docker.com/install/linux/docker-ce/

ubuntu/) are provided. After Docker installation, users must download and run a SIMON image from DockerHub. To do that users must run Terminal on Linux and MacOS or Windows Power Shell if using Windows OS and type following command:

docker run –rm –detach –name genular –tty –interactive –env IS_DOCKER='true' –env TZ=Europe/London –volume genular_data:/mnt/usrdata –publish 3010:3010 –publish 3011:3011 –publish 3012:3012 –publish 3013:3013 genular/simon:latest

Variable 'TZ=' stands for time zone and can be replaced with appropriate time zone. Once the command is executed, SIMON will be downloaded and started. To access SIMON, open a web browser (Firefox recommended, available at https://www.mozilla.org/firefox/) and type http://localhost:3010. Create an administrator account. SIMON will run until you shut down/restart your computer or stop it manually using the following command: docker stop genular. Advance instructions for installing a server version are provided on our GitHub page.

SUPPLEMENTAL INFORMATION

Supplemental Information can be found online at https://doi.org/10.1016/j.patter.2020.100178.

ACKNOWLEDGMENTS

We are grateful to all the individuals who participated in the research studies. We appreciate the helpful discussions with and support from many members of the Davis, Y. Chien, and Pollard labs. The clinical study on cyclists was supported by the NIHR Birmingham Biomedical Research Centre at the University Hospitals Birmingham NHS Foundation Trust and the University of Birmingham. The views expressed are those of the authors and not necessarily those of the NHS, the NIHR, or the Department of Health and Social Care. This work was supported by an NIH grant (U19 AI057229) and the Howard Hughes Medical Institute to M.M.D. and by the EU's Horizon 2020 research and innovation program under the Marie Sklodowska-Curie grant (FluPRINT, project 796636) to A.T.

AUTHOR CONTRIBUTIONS

A.T. and I.T. designed and developed SIMON, performed the analysis, processed and analyzed the data, and wrote the manuscript. L.W. and L.G. helped with the analysis of the Zeller and LIHC datasets, advised on analysis design, and revised the manuscript. M.K. helped with the integration of caret library and revised the manuscript. R.L.S., L.D., K.E.S., G.T., J.H., and A.J.P. conducted the VAST study, guided the analysis of the VAST dataset, pre-processed data for the analysis, and revised the manuscript. N.A.D., R.D.P., N.R.L., S.D.R.H., and J.M.L. performed the Cyclists study, provided the Cyclists data for the analysis, helped with the analysis, and revised the manuscript. P.K. guided the development of SIMON, supported standardization of the ML process in SIMON, and revised the manuscript. A.J.P. and M.M.D. supervised the study and revised and edited the manuscript.

DECLARATION OF INTERESTS

The authors declare no competing interests.

Received: August 23, 2020
Revised: October 27, 2020
Accepted: December 4, 2020
Published: January 8, 2021

REFERENCES

1. Stuart, T., and Satija, R. (2019). Integrative single-cell analysis. Nat. Rev. Genet. *20*, 257–272.
2. Nolan, J.P., and Condello, D. (2013). Spectral flow cytometry. Curr. Protoc. Cytom. *1*, 27.
3. Gregori, G., Patsekin, V., Rajwa, B., Jones, J., Ragheb, K., Holdman, C., and Robinson, J.P. (2012). Hyperspectral cytometry at the single-cell level using a 32-channel photodetector. Cytometry A *81*, 35–44.
4. Futamura, K., Sekino, M., Hata, A., Ikebuchi, R., Nakanishi, Y., Egawa, G., Kabashima, K., Watanabe, T., Furuki, M., and Tomura, M. (2015). Novel full-spectral flow cytometry with multiple spectrally-adjacent fluorescent proteins and fluorochromes and visualization of in vivo cellular movement. Cytometry A *87*, 830–842.
5. Bandura, D.R., Baranov, V.I., Ornatsky, O.I., Antonov, A., Kinach, R., Lou, X., Pavlov, S., Vorobiev, S., Dick, J.E., and Tanner, S.D. (2009). Mass cytometry: technique for real time single cell multitarget immunoassay based on inductively coupled plasma time-of-flight mass spectrometry. Anal. Chem. *81*, 6813–6822.
6. Bendall, S.C., Simonds, E.F., Qiu, P., Amir el-, A.D., Krutzik, P.O., Finck, R., Bruggner, R.V., Melamed, R., Trejo, A., Ornatsky, O.I., et al. (2011). Single-cell mass cytometry of differential immune and drug responses across a human hematopoietic continuum. Science *332*, 687–696.
7. Angelo, M., Bendall, S.C., Finck, R., Hale, M.B., Hitzman, C., Borowsky, A.D., Levenson, R.M., Lowe, J.B., Liu, S.D., Zhao, S., et al. (2014). Multiplexed ion beam imaging of human breast tumors. Nat. Med. *20*, 436–442.
8. Giesen, C., Wang, H.A., Schapiro, D., Zivanovic, N., Jacobs, A., Hattendorf, B., Schüffler, P.J., Grolimund, D., Buhmann, J.M., Brandt, S., and Varga, Z. (2014). Highly multiplexed imaging of tumor tissues with subcellular resolution by mass cytometry. Nat. Methods *11*, 417–422.
9. Bishop, C.M. (2006). Pattern Recognition and Machine Learning (Springer-Verlag).
10. Yip, K.Y., Cheng, C., and Gerstein, M. (2013). Machine learning and genome annotation: a match meant to be? Genome Biol. *14*, 205.
11. Chicco, D. (2017). Ten quick tips for machine learning in computational biology. BioData Min. *10*, 35.
12. Deo, R.C. (2015). Machine learning in medicine. Circulation *132*, 1920–1930.
13. Libbrecht, M.W., and Noble, W.S. (2015). Machine learning applications in genetics and genomics. Nat. Rev. Genet. *16*, 321–332.
14. Bonetta, R., and Valentino, G. (2020). Machine learning techniques for protein function prediction. Proteins *88*, 397–413.
15. Jurtz, V., Paul, S., Andreatta, M., Marcatili, P., Peters, B., and Nielsen, M. (2017). NetMHCpan-4.0: improved peptide-MHC class I interaction predictions integrating eluted ligand and peptide binding affinity data. J. Immunol. *199*, 3360–3368.
16. Lin, H.H., Ray, S., Tongchusak, S., Reinherz, E.L., and Brusic, V. (2008). Evaluation of MHC class I peptide binding prediction servers: applications for vaccine research. BMC Immunol. *9*, 8.
17. Kan, A. (2017). Machine learning applications in cell image analysis. Immunol. Cell Biol. *95*, 525–530.
18. Vamathevan, J., Clark, D., Czodrowski, P., Dunham, I., Ferran, E., Lee, G., Li, B., Madabhushi, A., Shah, P., Spitzer, M., and Zhao, S. (2019). Applications of machine learning in drug discovery and development. Nat. Rev. Drug Discov. *18*, 463–477.
19. Newell, E.W., Sigal, N., Bendall, S.C., Nolan, G.P., and Davis, M.M. (2012). Cytometry by time-of-flight shows combinatorial cytokine expression and virus-specific cell niches within a continuum of CD8+ T cell phenotypes. Immunity *36*, 142–152.
20. Horowitz, A., Strauss-Albee, D.M., Leipold, M., Kubo, J., Nemat-Gorgani, N., Dogan, O.C., Dekker, C.L., Mackey, S., Maecker, H., Swan, G.E., and Davis, M.M. (2013). Genetic and environmental determinants of human NK cell diversity revealed by mass cytometry. Sci. Transl. Med. *5*, 208ra145.
21. Chaudhury, S., Duncan, E.H., Atre, T., Storme, C.K., Beck, K., Kaba, S.A., Lanar, D.E., and Bergmann-Leitner, E.S. (2018). Identification of immune signatures of novel adjuvant formulations using machine learning. Sci. Rep. *8*, 17508.
22. Chaudhury, S., Duncan, E.H., Atre, T., Dutta, S., Spring, M.D., Leitner, W.W., and Bergmann-Leitner, E.S. (2020). Combining immunoprofiling

with machine learning to assess the effects of adjuvant formulation on human vaccine-induced immunity. Hum. Vaccin. Immunother. *16*, 400–411.

23. Warsinske, H.C., Rao, A.M., Moreira, F.M.F., Santos, P.C.P., Liu, A.B., Scott, M., Malherbe, S.T., Ronacher, K., Walzl, G., Winter, J., et al. (2018). Assessment of validity of a blood-based 3-gene signature score for progression and diagnosis of tuberculosis, disease severity, and treatment response. JAMA Netw. Open *1*, e183779.
24. Robinson, M., Sweeney, T.E., Barouch-Bentov, R., Sahoo, M.K., Kalesinskas, L., Vallania, F., Sanz, A.M., Ortiz-Lasso, E., Albornoz, L.L., Rosso, F., and Montoya, J.G. (2019). A 20-gene set predictive of progression to severe dengue. Cell Rep. *26*, 1104–1111.e4.
25. Sweeney, T.E., Perumal, T.M., Henao, R., Nichols, M., Howrylak, J.A., Choi, A.M., Bermejo-Martin, J.F., Almansa, R., Tamayo, E., Davenport, E.E., and Burnham, K. (2018). A community approach to mortality prediction in sepsis via gene expression analysis. Nat. Commun. *9*, 694.
26. Mayhew, M.B., Buturovic, L., Luethy, R., Midic, U., Moore, A.R., Roque, J.A., Shaller, B.D., Asuni, T., Rawling, D., Remmel, M., et al. (2020). A generalizable 29-mRNA neural-network classifier for acute bacterial and viral infections. Nat. Commun. *11*, 1177.
27. Kourou, K., Exarchos, T.P., Exarchos, K.P., Karamouzis, M.V., and Fotiadis, D.I. (2015). Machine learning applications in cancer prognosis and prediction. Comput. Struct. Biotechnol. J. *13*, 8–17.
28. Beckmann, J.S., and Lew, D. (2016). Reconciling evidence-based medicine and precision medicine in the era of big data: challenges and opportunities. Genome Med. *8*, 134.
29. R Development Core Team. (2013). R: A Language and Environment for Statistical Computing. http://www.R-project.org.
30. Kuhn, M. (2008). Building predictive models in R using the caret package. J. Stat. Softw. *28*, 1–26.
31. Guttag, J.V. (2016). Introduction to Computation and Programming Using Python: With Application to Understanding Data (The MIT Press).
32. Lang, M., Binder, M., Richter, J., Schratz, P., Pfisterer, F., Coors, S., Au, Q., Casalicchio, G., Kotthoff, L., Bischl, B., et al. (2019). mlr3: A modern object-oriented machine learning framework in R. J. Open Source Softw. *4*, 1903, https://doi.org/10.21105/joss.01903.
33. Kuhn, M., Contributions from, Jed Wing, S.W., Williams, A., Keefer, C., Engelhardt, Allan, Cooper, Tony, Mayer, Zachary, Kenkel, Brenton, the R Core Team, Benesty, Michael, Lescarbeau, Reynald, Ziem, Andrew, et al. (2018). Caret: classification and regression training. https://CRAN.R-project.org/package=caret.
34. Pedregosa, F., Varoquaux, G., Gramfort, A., Michel, V., Thirion, B., Grisel, O., Blondel, M., Prettenhofer, P., Weiss, R., Dubourg, V., et al. (2011). Scikit-learn: machine learning in python. J. Machine Learn. Res. *12*, 2825–2830.
35. Albanese, D., Visintainer, R., Merler, S., Riccadonna, S., Jurman, G., and Furlanello, C. (2012). Mlpy: machine learning Python. https://arxiv.org/abs/1202.6548.
36. Bell, M.L., Fiero, M., Horton, N.J., and Hsu, C.H. (2014). Handling missing data in RCTs; a review of the top medical journals. BMC Med. Res. Methodol. *14*, 118.
37. Pes, B. (2019). Handling Class Imbalance in High-Dimensional Biomedical Datasets. 8th International Conference on Enabling Technologies: Infrastructure for Collaborative Enterprises (WETICE). Napoli, Italy. https://doi.org/10.1109/WETICE.2019.00040.
38. Bellman, R.E. (1957). Dynamic Programming (Princeton University Press).
39. Tomic, A., Tomic, I., Rosenberg-Hasson, Y., Dekker, C.L., Maecker, H.T., and Davis, M.M. (2019). SIMON, an automated machine learning system, reveals immune signatures of influenza vaccine responses. J. Immunol. *203*, 749–759.
40. Tusher, V.G., Tibshirani, R., and Chu, G. (2001). Significance analysis of microarrays applied to the ionizing radiation response. Proc. Natl. Acad. Sci. U S A. *98*, 5116–5121.
41. Murty, J. (2009). Programming Amazon Web Services: S3, EC2, SQS, FPS, and SimpleDB (O'Reilly Media).
42. Sippy, R., Farrell, D.F., Lichtenstein, D.A., Nightingale, R., Harris, M.A., Toth, J., Hantztidiamantis, P., Usher, N., Cueva Aponte, C., and Barzallo Aguilar, J. (2020). Severity Index for Suspected Arbovirus (SISA): machine learning for accurate prediction of hospitalization in subjects suspected of arboviral infection. Plos Negl. Trop. Dis. *14*, e0007969.
43. Duggal, N.A., Pollock, R.D., Lazarus, N.R., Harridge, S., and Lord, J.M. (2018). Major features of immunesenescence, including reduced thymic output, are ameliorated by high levels of physical activity in adulthood. Aging Cell *17*, https://doi.org/10.1111/acel.12750.
44. Dahora, L.C., Jin, C., Spreng, R.L., Feely, F., Mathura, R., Seaton, K.E., Zhang, L., Hill, J., Jones, E., Alam, S.M., et al. (2019). IgA and IgG1 specific to Vi polysaccharide of salmonella typhi correlate with protection status in a typhoid fever controlled human infection model. Front. Immunol. *10*, 2582.
45. Zeller, G., Tap, J., Voigt, A.Y., Sunagawa, S., Kultima, J.R., Costea, P.I., Amiot, A., Böhm, J., Brunetti, F., Habermann, N., et al. (2014). Potential of fecal microbiota for early-stage detection of colorectal cancer. Mol. Syst. Biol. *10*, 766.
46. Pasolli, E., Schiffer, L., Manghi, P., Renson, A., Obenchain, V., Truong, D.T., Beghini, F., Malik, F., Ramos, M., Dowd, J.B., and Huttenhower, C. (2017). Accessible, curated metagenomic data through ExperimentHub. Nat. Methods *14*, 1023–1024.
47. Geistlinger, L., Csaba, G., Santarelli, M., Ramos, M., Schiffer, L., Turaga, N., Law, C., Davis, S., Carey, V., Morgan, M., et al. (2020). Toward a gold standard for benchmarking gene set enrichment analysis. Brief Bioinform. https://doi.org/10.1093/bib/bbz158.
48. Jin, C., Gibani, M.M., Moore, M., Juel, H.B., Jones, E., Meiring, J., Harris, V., Gardner, J., Nebykova, A., Kerridge, S.A., et al. (2017). Efficacy and immunogenicity of a Vi-tetanus toxoid conjugate vaccine in the prevention of typhoid fever using a controlled human infection model of Salmonella Typhi: a randomised controlled, phase 2b trial. Lancet *390*, 2472–2480.
49. Mkhadri, A. (1995). Shrinkage parameter for the modified linear discriminant analysis. Pattern Recogn. Lett. *16*, 267–275.
50. Bouveyron, C., Girard, S., and Schmid, C. (2007). High-dimensional discriminant analysis. Commun. Stat. Theor. Methods *36*, 2607–2623.
51. Ripley, B.D. (1996). Pattern Recognition and Neural Networks (Cambridge University Press).
52. Davis, J. and Goadrich, M. (2006). The relationship between precision-recall and ROC curves. Proceedings of the 23rd International Conference on Machine Learning. Pittsburgh, PA. https://doi.org/10.1145/1143844.1143874.
53. Sarathi, A., and Palaniappan, A. (2019). Novel significant stage-specific differentially expressed genes in hepatocellular carcinoma. BMC Cancer *19*, 663.
54. Oike, Y., Akao, M., Yasunaga, K., Yamauchi, T., Morisada, T., Ito, Y., Urano, T., Kimura, Y., Kubota, Y., Maekawa, H., et al. (2005). Angiopoietin-related growth factor antagonizes obesity and insulin resistance. Nat. Med. *11*, 400–408.
55. Lu, S., Lu, H., Jin, R., and Mo, Z. (2019). Promoter methylation and H3K27 deacetylation regulate the transcription of VIPR1 in hepatocellular carcinoma. Biochem. Biophys. Res. Commun. *509*, 301–305.
56. Xu, Z.G., Du, J.J., Zhang, X., Cheng, Z.H., Ma, Z.Z., Xiao, H.S., Yu, L., Wang, Z.Q., Li, Y.Y., Huo, K.K., and Han, Z.G. (2003). A novel liver-specific zona pellucida domain containing protein that is expressed rarely in hepatocellular carcinoma. Hepatology *38*, 735–744.
57. Witten, I.H., Frank, E., and Hall, M.A. (2016). The Weka workbench. In Data Mining: Practical Machine Learning Tools and Techniques (Morgan Kaufmann).
58. Demšar, J., Curk, T., Erjavec, A., Gorup, Č., Hočevar, T., Milutinovič, M., Možina, M., Polajnar, M., Toplak, M., Starič, A., et al. (2013). Orange: Data Mining Toolbox in Python. J. Mach. Learn. Res. *14*, 2349–2353.
59. Berthold, M.R., Cebron, N., Dill, F., Gabriel, T.R., Kötter, T., Meinl, T., Ohl, P., Thiel, K., Wiswedel, B., et al. (2009). KNIME: the Konstanz information

miner. ACM SIGKDD Explorations Newsletter *11*, https://doi.org/10.1145/1656274.1656280.

60. Achtert, E., Kriegel, H. and Zimek, A. (2008). ELKI: A Software System for Evaluation of Subspace Clustering Algorithms. 20th International Conference on Scientific and Statistical Database Management (SSDBM 2008). pp. 580-585.
61. Hutter, F., Kotthoff, L., and Vanschoren, J. (2018). Automated Machine Learning: Methods, Systems, Challenges (Springer).
62. Thornton, C., Hutter, F., Hoos, H.H., and Leyton-Brown, K. (2013). Auto-WEKA: combined selection and hyperparameter optimization of classification algorithms. Knowled. Discov. Data Mining *2013*, https://doi.org/10.1145/2487575.2487629.
63. Kotthoff, L., Thornton, C., Hoos, H.H., Hutter, F., and Leyton-Brown, K. (2016). Auto-WEKA 2.0: Automatic model selection and hyperparameter optimization in WEKA. J. Mach. Learn. Res. *17*, 1–5.
64. Feurer, M., Klein, A., Eggensperger, K., Springenberg, J., Blum, M., and Hutter, F. (2015). Efficient and robust automated machine learning. Adv. Neural Inf. Process. Syst. *28*, https://doi.org/10.5555/2969442.2969547.
65. Olson, R.S., Urbanowicz, R.J., Andrews, P.C., Lavender, N.A., Kidd, L.C., and Moore, J.H. (2016). Automating biomedical data science through tree-based pipeline optimization. Appl. Evol. Comput. 123–137, https://doi.org/10.1007/978-3-319-31204-0_9.
66. Alaa, A. and Schaar, M. (2018). AutoPrognosis: automated clinical prognostic modeling via bayesian optimization with structured kernel learning. International Conference on Machine Learning. http://medianetlab.ee.ucla.edu/papers/ICML2018_AP.pdf
67. Kuhn, M., and Johnson, K. (2019). Feature Engineering and Selection: A Practical Approach for Predictive Models (Chapman and Hall/CRC).
68. Tomic, A. (2020). Analysis of human humoral responses in a typhoid vaccine efficacy trial used for SIMON analysis. Zenodo. https://doi.org/10.5281/zenodo.4121322.
69. Tomic, A. (2020). Dataset of clinical biomarkers for prediction of the arboviral infection severity using SIMON analysis. Zenodo. https://doi.org/10.5281/zenodo.4121831.
70. Tomic, A. (2020). Immunophenotyping dataset of master cylists used for SIMON analysis. Zenodo. https://doi.org/10.5281/zenodo.4115626.
71. Tomic, A. (2020). Microbiome dataset used in SIMON analysis for early-stage detection of cancer. Zenodo. https://doi.org/10.5281/zenodo.4121516.
72. Tomic, A. (2020). RNA sequencing dataset for prediction of liver hepatocellular carcinoma using SIMON analysis. Zenodo. https://doi.org/10.5281/zenodo.4121594.

PATTER, Volume *2*

Supplemental Information

SIMON: Open-Source Knowledge Discovery Platform

Adriana Tomic, Ivan Tomic, Levi Waldron, Ludwig Geistlinger, Max Kuhn, Rachel L. Spreng, Lindsay C. Dahora, Kelly E. Seaton, Georgia Tomaras, Jennifer Hill, Niharika A. Duggal, Ross D. Pollock, Norman R. Lazarus, Stephen D.R. Harridge, Janet M. Lord, Purvesh Khatri, Andrew J. Pollard, and Mark M. Davis

Supplemental materials

Supplemental Experimental Procedures

Step-by-step instructions how to run SIMON analysis for the following use cases: (1) Identifying clinical biomarkers that can predict the severity of the arboviral infection severity; (2) Predicting antibody signature to mediate protection against *Salmonella* Typhi challenge infection; (3) Identifying cellular immune signature associated with high-level of physical activity; (4) Building predictive model for the early-stage detection of colorectal cancer using microbiome; and (5) Building predictive model for detection of liver hepatocellular carcinoma cells using transcriptome data.

Use case 1. Identifying clinical biomarkers that can predict the severity of the arboviral infection severity.

Step 1. Uploading data. SISA dataset (available as **Supplementary table 2**) needs to be uploaded as CSV file in the following format: donors/samples in rows and features that were measured (i.e. clinical measurements) in columns (*'Click here'* red arrow on image below). One of the columns contains information about the outcome, in this case this is the column named *'Hospitalized'* and the outcome is labelled with zero if '*non-hospitalized'* and with one is *'hospitalized'*. Note that SIMON can analyze data using either text or numeric values for the outcome variable.

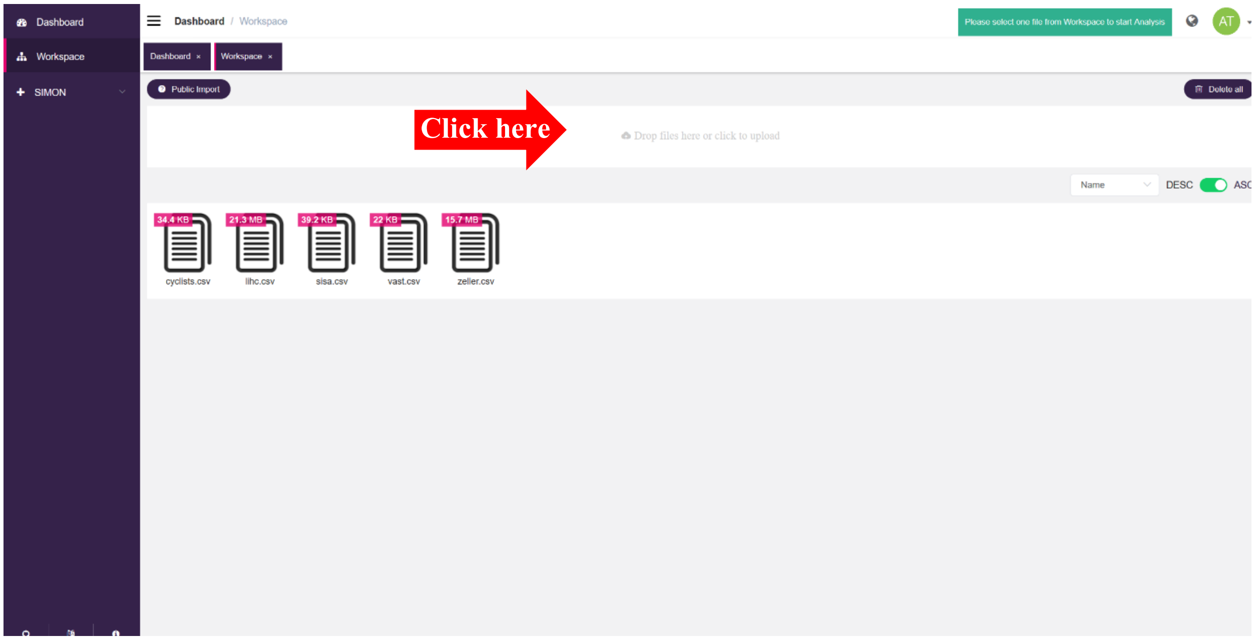

Step 2. Selecting dataset. To start the analysis, user must select the SISA dataset by clicking the document icon (A). The selected SISA dataset will be highlighted in grey and in the upper right-hand side the green

tab will show the name of the SISA dataset (B). Now in the left-hand side menu (in purple) the '*Analysis*' tab becomes available (C).

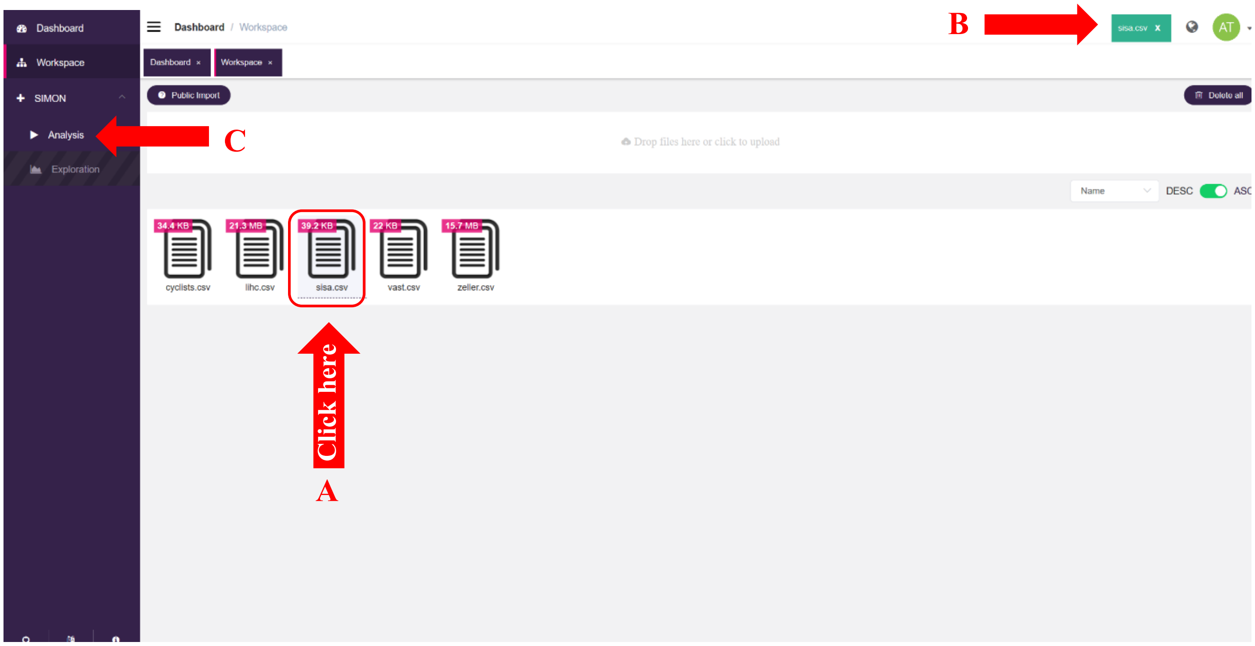

Step 3. Setting-up analysis. Once SISA dataset is selected, users can start with the analysis by clicking the '*Analysis*' on the left-hand side menu (in purple). This opens new window '*Analysis*' where users can select analysis parameters and ML algorithms. For the SISA dataset, in the Box 1, users must select all predictor variables by clicking the button next to the input form (A) since we want to use all clinical measurements for the prediction model. Alternatively, if users want to select only some features, by clicking in the input field '*Predictor variables*' first 50 available columns are shown in the drop-down menu and users can choose which columns they want to use for analysis. If there are more than 50 columns available, users can type which columns they want to use. Next, we select the outcome we want to predict in the '*Response*' input field, in the SISA dataset that is the '*Hospitalized*' column (B). We then select which columns to exclude (C). In the SISA dataset we have excluded column without any information for the predictive model (donor identification numbers in the *IDindex* column). The initial SISA dataset is split into training (85% of the data) and test sets (15% of the data) (D). Finally, for the pre-processing step, data was centered (mean subtracted from values) and scaled (values divided by standard deviation) (E).

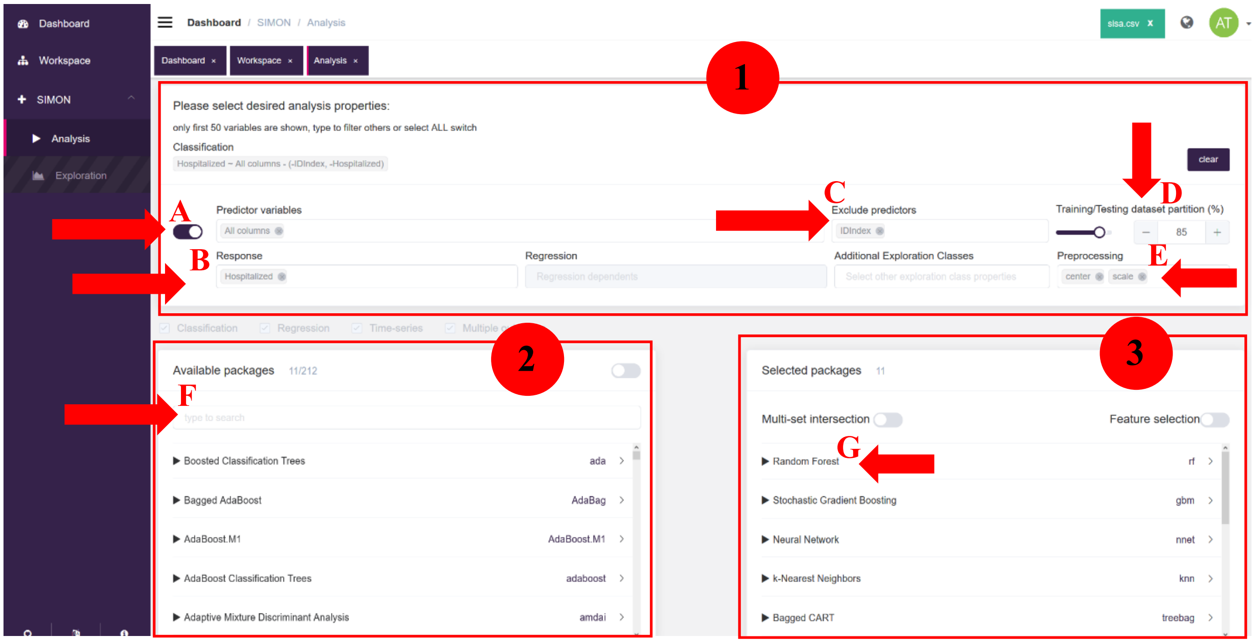

Users can choose which pre-processing functions they want to apply. Available pre-processing functions are Box-Cox transformation (BoxCox), power transformation (expoTrans), Yeo-Johnson transformation (YeoJohnson), subtract mean from values (center), divide by standard deviation (scale), normalize values (range), K-nearest neighbors Imputation (knnimpute), Imputation using Bagging of regression tress (bagImpute), median impute (medianImpute), prinicipal component analysis (pca), data projected onto a unit circle (spatialSign), correlation filtering (corr), remove zero-variance (zv), remove near zero-variance (nzv) and exclude predictors that have only one unique value (conditional).

In the Box 2, users select which ML algorithms to use, while 5 of the default ML algorithms are already selected in the Box 3. For the analysis of the SISA dataset, we will, in addition to five already selected, select additional six ML algorithms: shrinkage discriminant analysis, treebag, k nearest neighbors, random forest, stochastic generalized boosting model and neural network. Name of the ML algorithm is typed in the input field (F). The full names of the packages for the selected algorithms are: *'Shrinkage discriminant analysis'*, Shrinkage discriminant analysis (sda); *'Treebag'*, Bagged CART; *'k nearest neighbors'*, k-Nearest Neighbors (knn); *'Random forest',* Random forest (rf); '*Stochastic generalized boosting model'*, Stochastic gradient boosting (gbm) and *'Neural network',* Neural network (nnet). Once the name of the algorithm is typed and user clicks on the desired package, that algorithm is automatically added to the list of selected algorithms in the Box 3. Note, that sometimes different R packages are available for same algorithm, as it is the case for Random forest algorithm. SIMON allows users to inspect selected algorithms by clicking on their names (G). Users then obtain additional information about the algorithm (H) and they

can click to obtain the reference to the original publication (I) to be sure that they select appropriate algorithms.

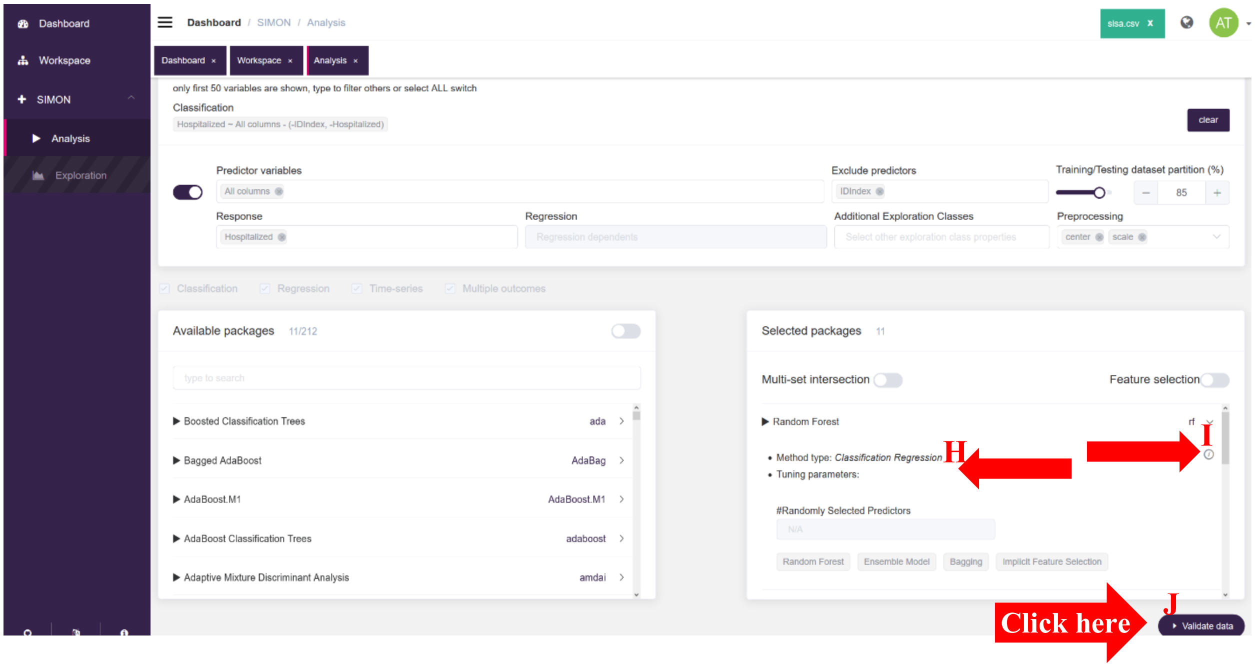

Finally, analysis is initiated by clicking the *'Validate data'* button (J). The following screen shows and analysis is started by clicking on the *'Process'* button (K).

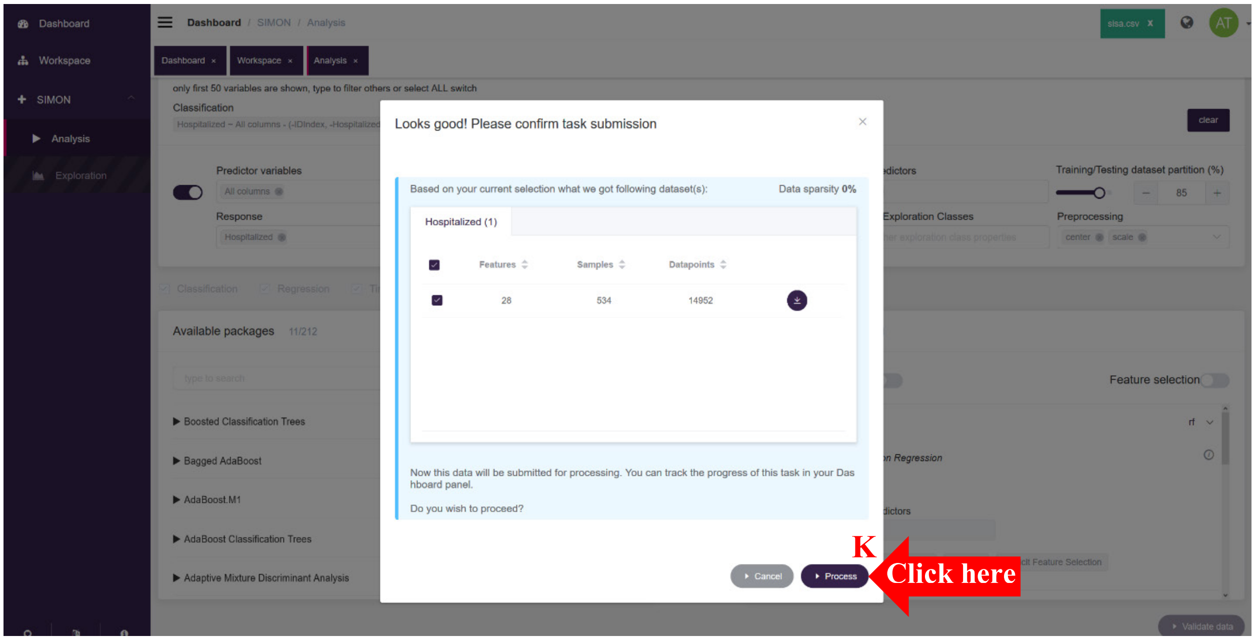

New window *'Dashboard'* will open immediately and SISA analysis will be created and initiated (L). To assess the current status of the analysis, we look under *'Status'* column (M), *'Processing'* means that the analysis is going, once analysis is complete it says *'Completed'* in green. *'Sparsity (%)'* column tells us the

percentage of missing values in the dataset; *'Models processed'*, number of processed models; *'Successful models'*, number of successfully finished models. In the *'Operations'* column users can get information about the models and their performance measures during the analysis (first button, "circled *i*" icon), download dataset (second full green button) and delete the analysis (third red button).

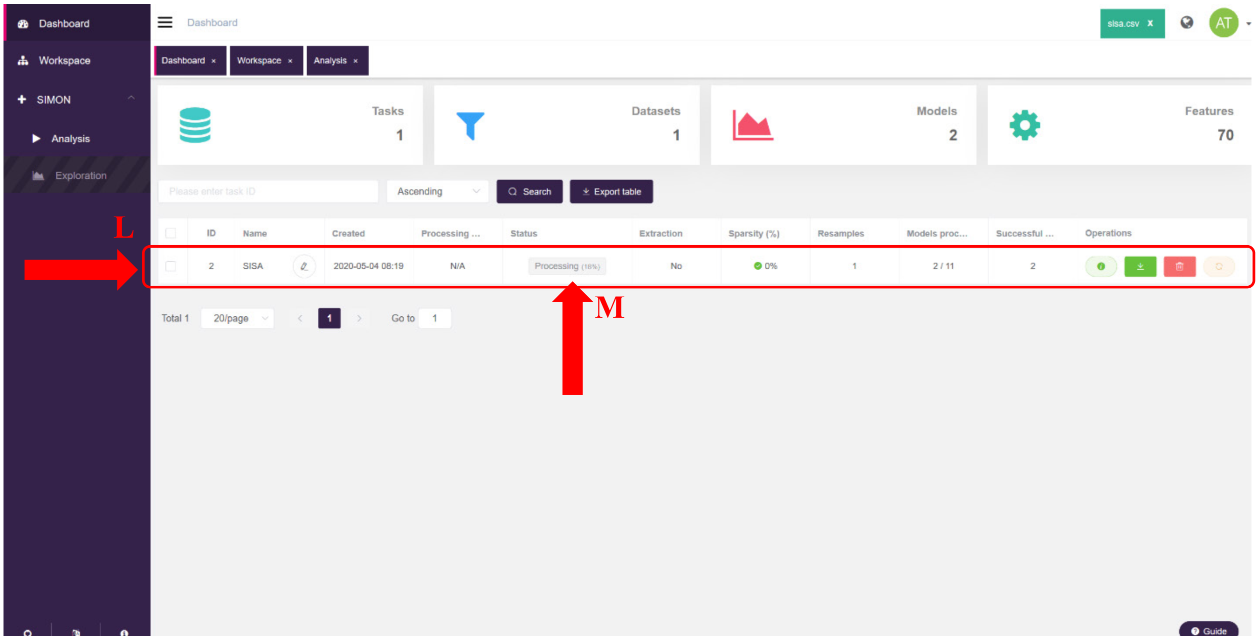

Step 4. Model evaluation and selection. After the analysis is done, users can explore built predictive models by clicking on the checkbox next to the SISA analysis row (A). Upon selection of SISA analysis row, the *'Exploration'* tab (B) becomes available in the menu on the left-hand side. By clicking on the *'Exploration'* tab, new window opens where users can explore built models.

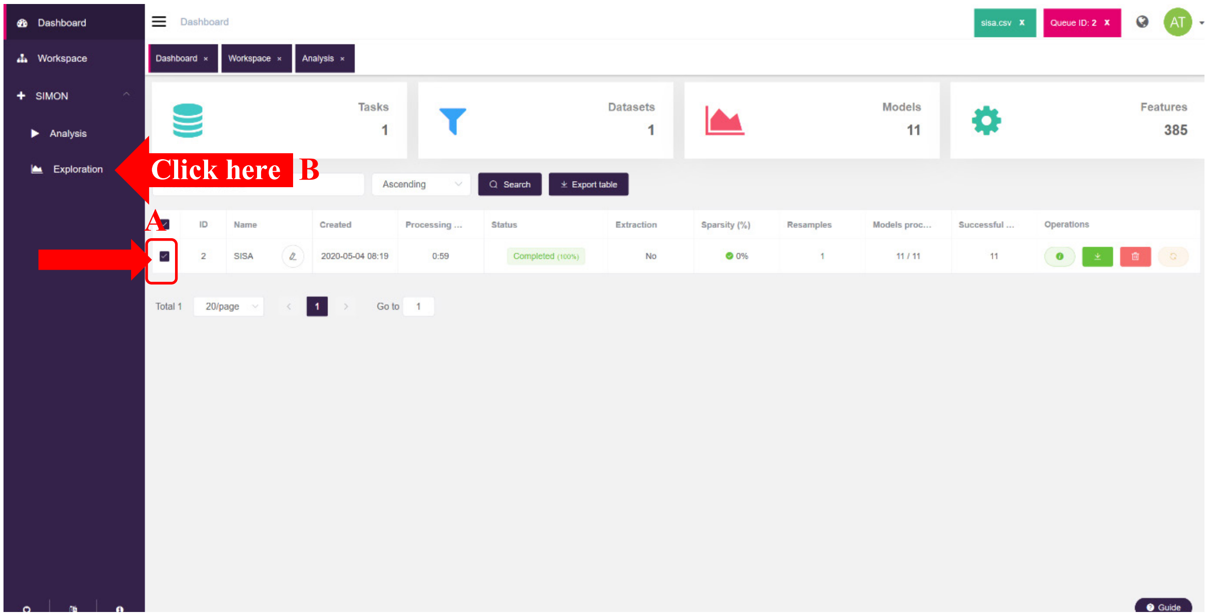

Now, in the new window, users select desired performance measurements by clicking on the drop-down menu in the input Box 1. SIMON calculates different performance measurements for training set (train AUC, train F1, train prAUC, train recall, train precision, train sensitivity and train specificity) and test set (accuracy, F1, kappa, predict AUC, predict prAUC, precision, recall, sensitivity and specificity). For the SISA dataset, we choose AUROC as performance measurement by selecting train AUC and predict AUC. Now, we click in the dataset in the Box 2 (C). This opens Box 3 containing table of all models that were built. We order models based on the train AUC value (D). The model built with the highest train AUC was built with the *sda* algorithm. To compare models, users select desired models by clicking the check box next to each model. We will compare top five models by clicking in the 'select all' check box (E). Once all five models are selected, users can download the table with all models and performance measurements as CSV file and models with the data as RData objects by clicking on F. The initial dataset, training and test set can be saved as CSV files by clicking the download icon next to the dataset row in the Box 2 (G).

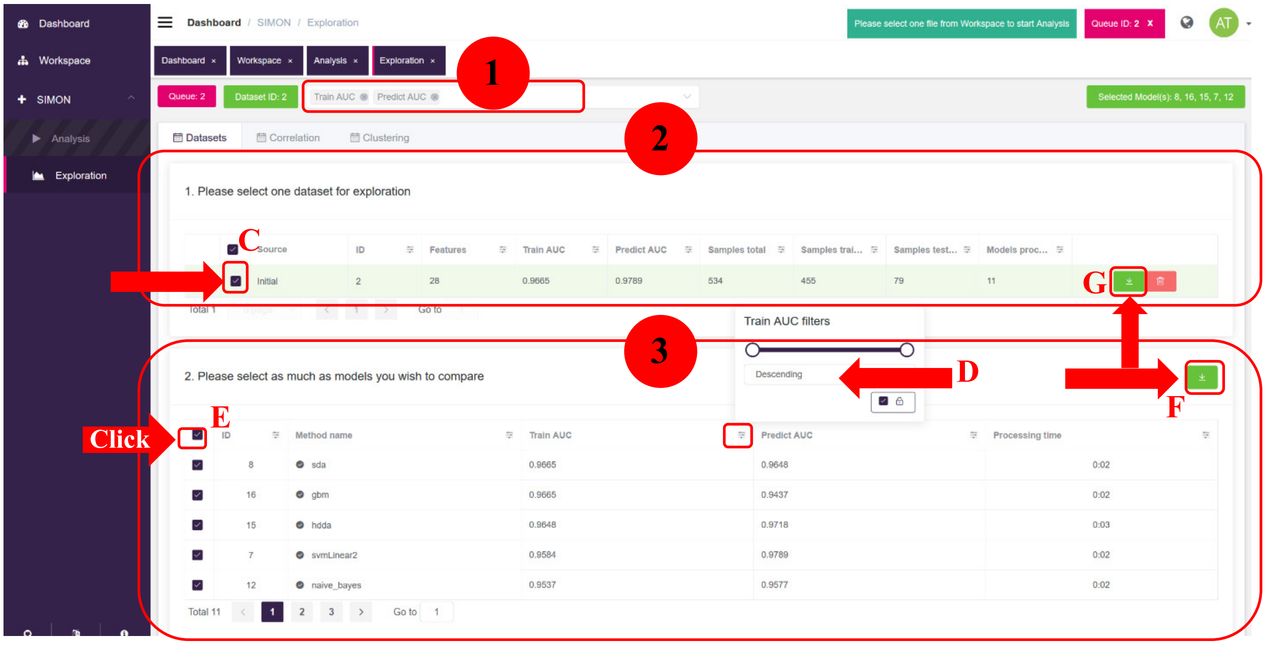

By selecting models to compare, the *'Training Summary'* tab will appear below Box 3 (H). Users must select at least two models for the tab to appear. Here, users visualize model comparison and can download box plots graphs showing performance measures calculated for the training set (I) and ROC plots for the training set (J) for all models selected as SVG files. To select all 11 models, as we did in the Figure 1, users must navigate to pages 2 and 3 and click select all check box.

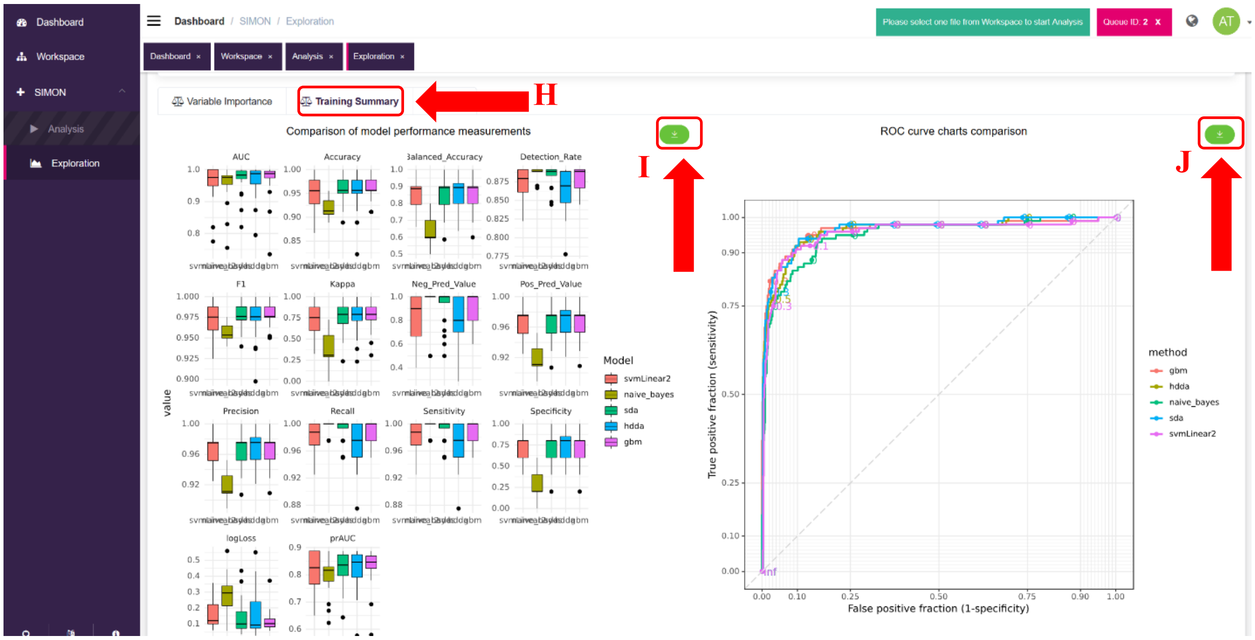

Step 5. Feature selection. To explore features that contributed the most to the sda model, we select only sda model in the Box 3 and click the *'Variable importance'* tab next to the *Training Summary'* tab (A). This opens table where features are ranked based on the Variable Importance Score (*'Score'* column). Features that have Variable Importance score above 50 are highlighted in green (B). The table can be downloaded as the CSV file by clicking download button (C). Users can select two or more models and compare ranking of features across models.

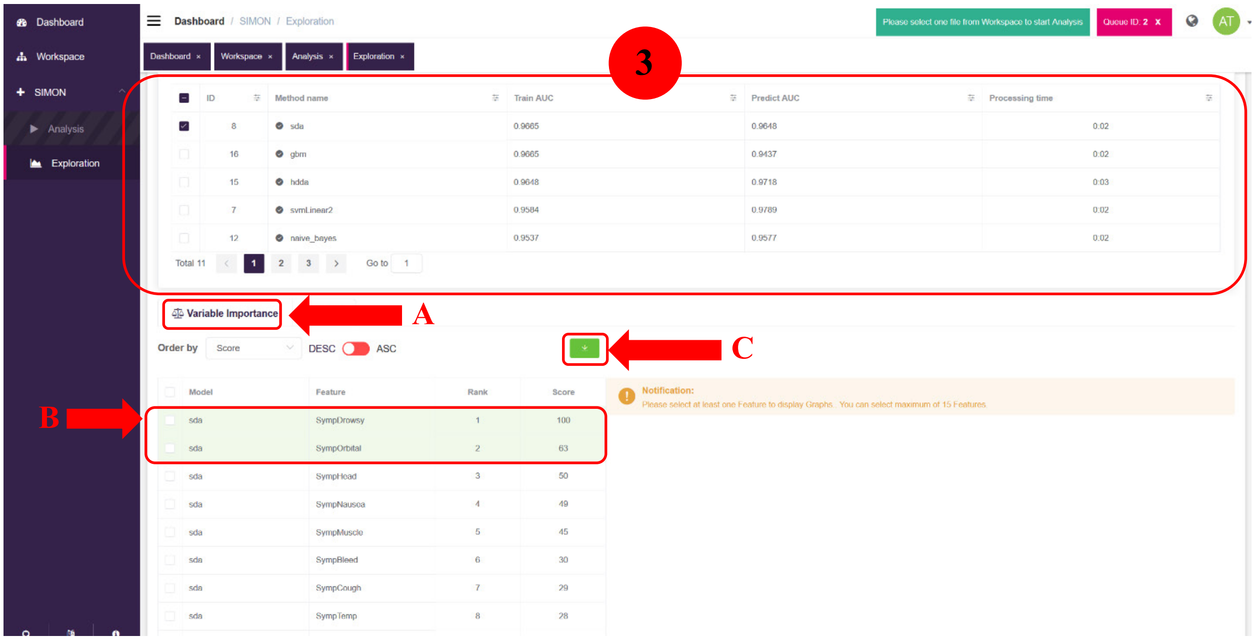

By selecting desired features, users can visualize distribution of data between both groups using dot plots. Plots can be adjusted by selecting *'Theme'*, *'Color'*, font size (*'Font'*), dot size (*'Dot'*) and height/width ratio (*'Ratio'*) as described in the ggplot2 R package (https://ggplot2.tidyverse.org/) (D). To apply changes to the graphs users must press *'Redraw plot'* red button (E) and graphs can be downloaded as SVG files by pressing download button (F).

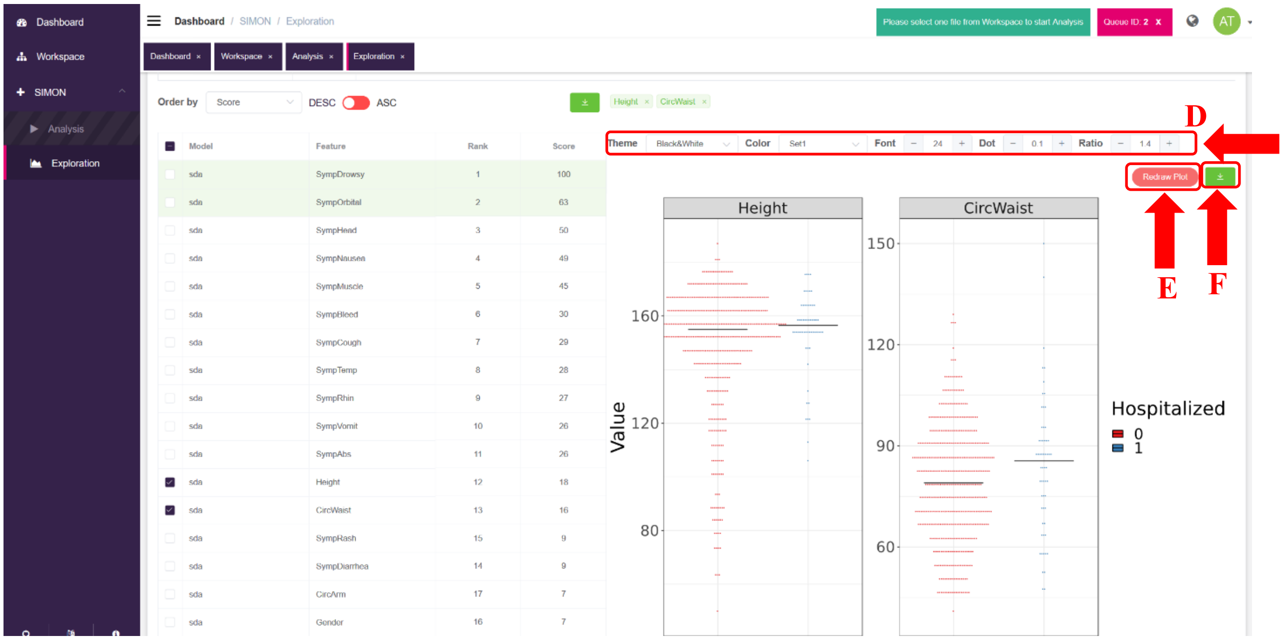

Step 6. Exploratory analysis. In the 'Exploration' window, upon selection of dataset for analysis, two tabs are shown: *'Correlation'* and *'Clustering'* (A). By clicking on *'Correlation'* tab, users can perform correlation analysis on the selected dataset using three different correlation methods (Pearson, Kendall and Spearman) and different parameters can be applied by clicking the *'Plot image'* red button (B). Correlation plot can be saved as SVG file by clicking download button (C). *'Clustering'* tab will be explained in the Use case 2.

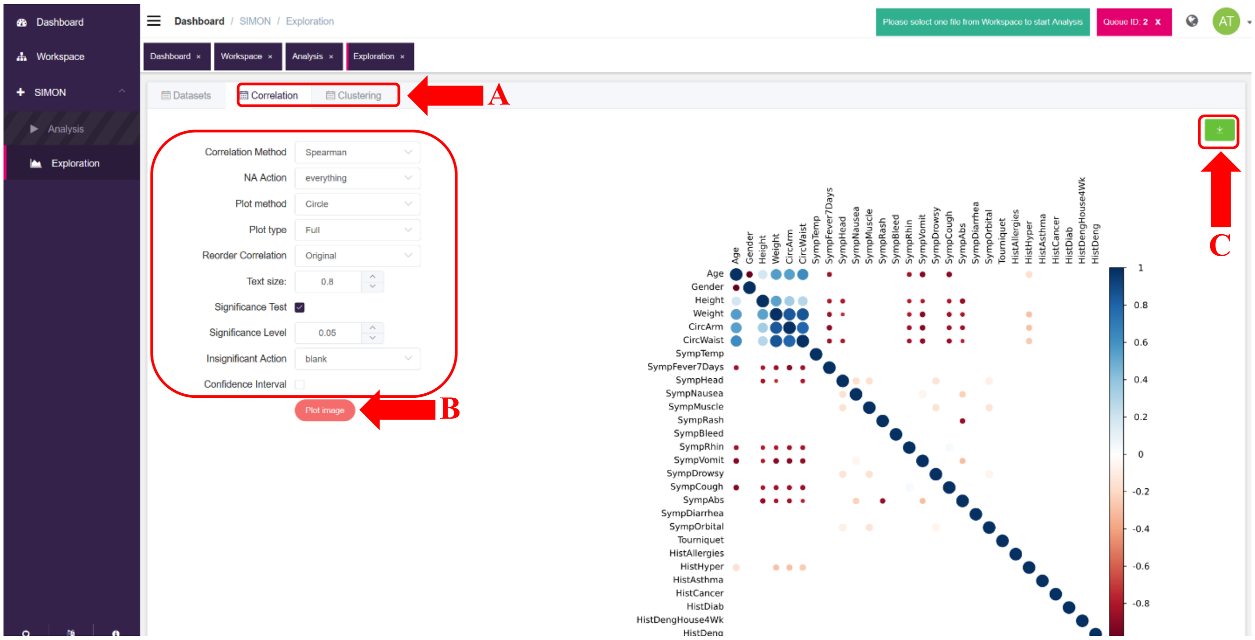

Use case 2. Predicting antibody signature to mediate protection against *Salmonella* Typhi challenge infection.

The first two steps (Step 1. Uploading data and Step 2. Selecting dataset.) are the same as explained under Use case 1, therefore we will start from Step 3.

Step 3. Setting-up analysis. To perform SIMON analysis on the VAST dataset, users must select all predictor variables by clicking the button next to the *'Predictor variables'* input form (A) and *'Diagnosis'* column as the outcome in the *'Response'* input form (B). *'Vaccine'* column is selected under *'Additional Exploration Classes'* (C). The initial dataset is split into training (75% of the data) and test sets (25% of the data) (D) and we applied 'center' and 'scale' as pre-processing steps (E). In total, five ML algorithms were selected (F). Since the VAST dataset has missing values, in the first step of SIMON we will use *'Multi-set intersection'* function (G).

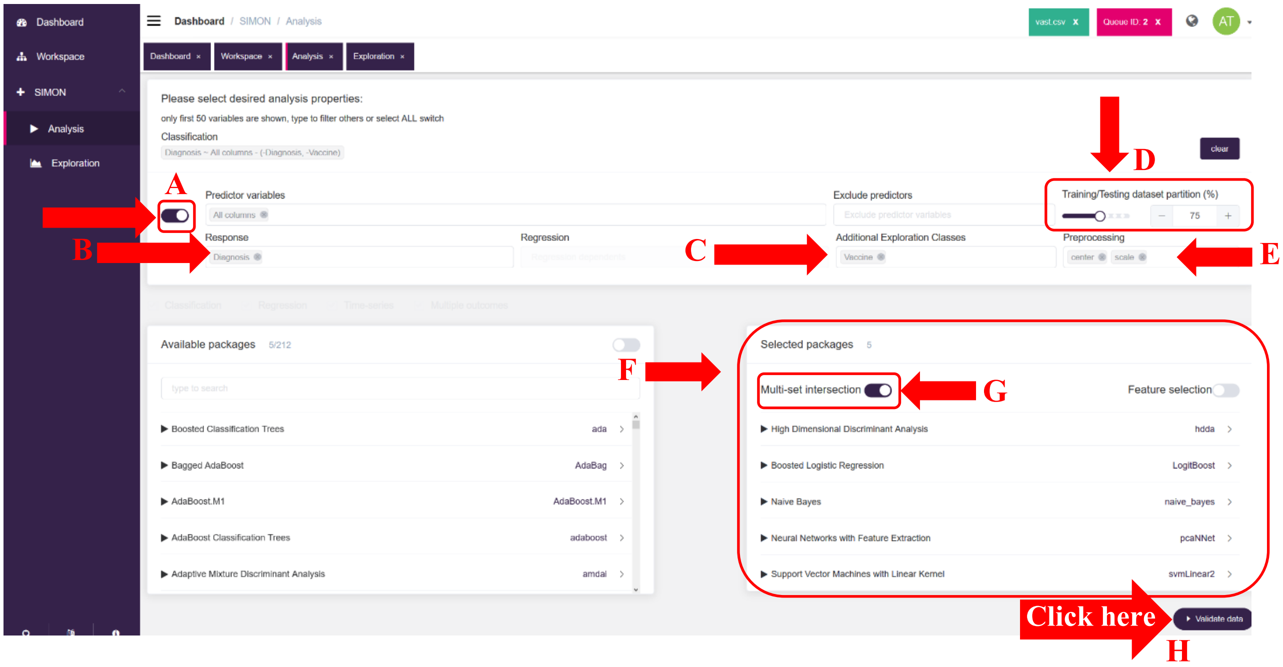

By clicking *'Validate data'* button (H), multi-set intersection function will generate resamples and the pop-up window shows 206 generated resamples with different number of 'Features' and donors ('Samples' column). Each resample can be saved by clicking on the download button and analysis can be performed by selected resamples. In the VAST dataset, we performed analysis on 58 resamples with 40 or more samples in total. Click *'Process'* button to start analysis.

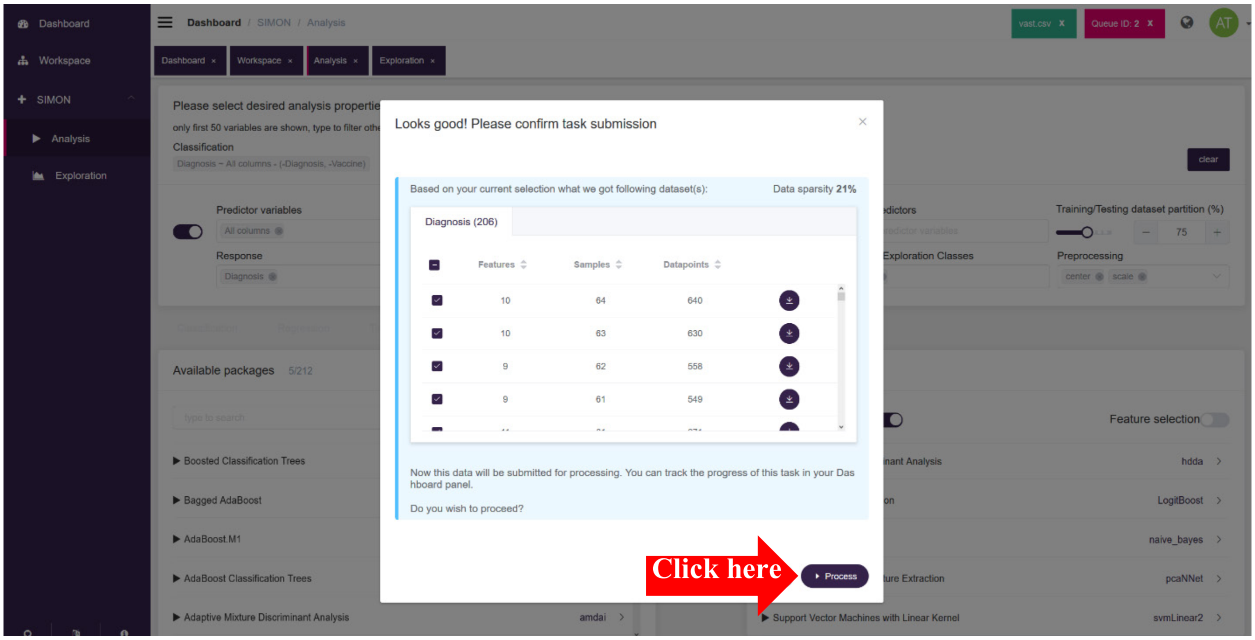

Step 4. Model evaluation and selection. We open *'Exploration'* window (tab becomes available upon selection of VAST analysis row in the *'Dashboard'*) and select train AUC and predict AUC as performance

measurements (A). Then, we order datasets (i.e. resamples) based on the maximum train AUC value and using slider remove all models with train AUC value below 0.72 (B). Now, we select the first dataset with 10 ‘Features’ (C) and 45 ‘Samples total’ (D) and in the table below, we explore all models built for that dataset by ordering models based on the train AUC (E). The model built with the highest performace measurements was built with the pcaNNet algorithm (F), but despite high train AUC value, that model does not perform so well on the unseen test data (predict AUC 0.64). Such a model is considered overfitted. We must examine other resamples to find optimal models with better perfomance on the test data. Each dataset/resample and accompanying split into training and test sets can be saved as CSV file by clicking the download button (G). To highest perfoming model was built on the dataset/resample that has 13 features and 47 donors (samples) (ID 38). We select that dataset and evaluate models using box plots and ROC curves as described for Use case 1.

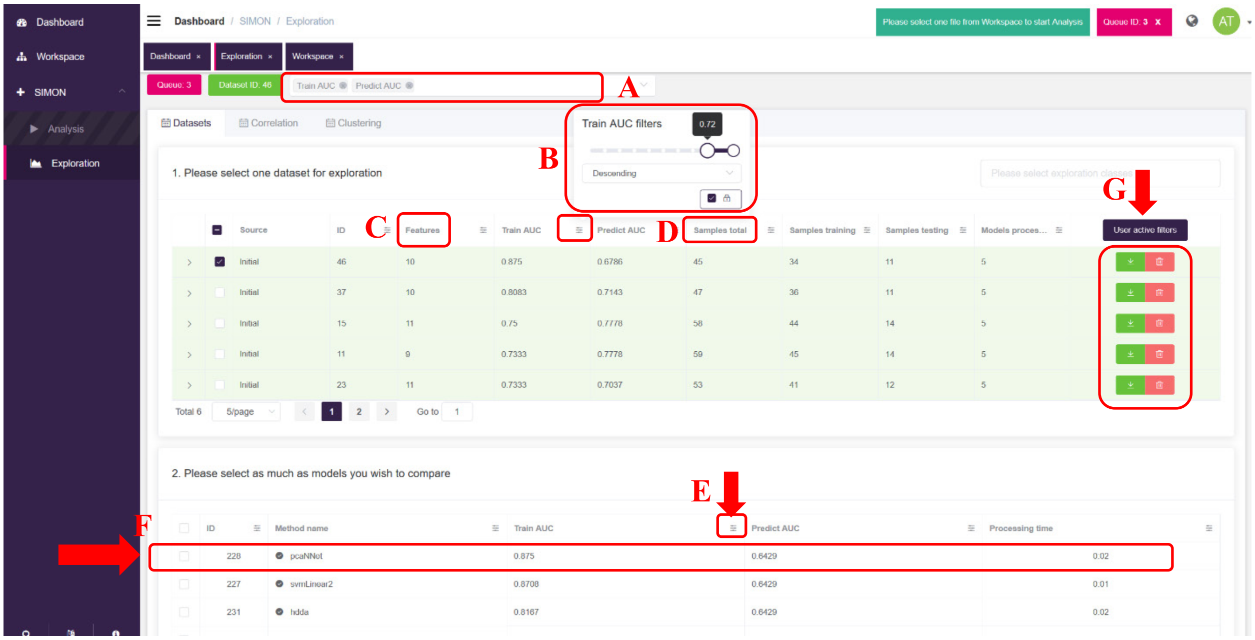

Step 5. Feature selection. Upon selecting the best performing model built with the naïve Bayes algorithm, we can explore the features that contributed the most to this model in the *‘Variable importance’* tab. The variable importance score table can be downloaded as a CSV file and graphs as SVG files by clicking download buttons (A and B).

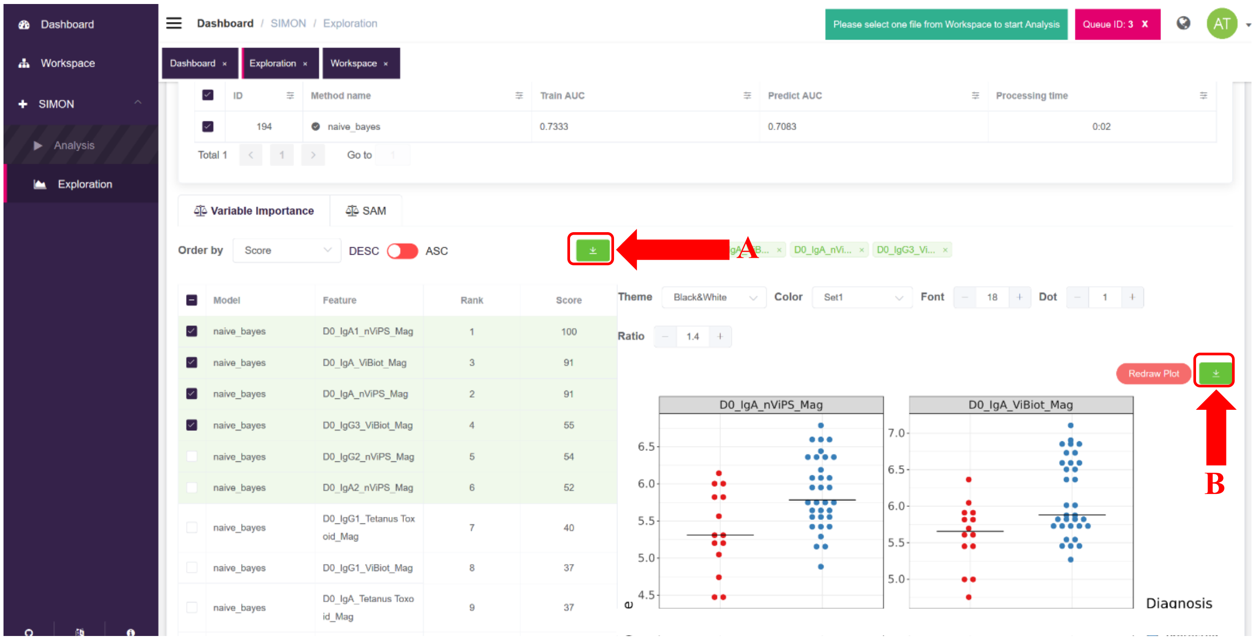

Step 6. Exploratory analysis. In addition to the *'Correlation'* tab explained above in the Use case 1, users can perform clustering analysis in the *'Clustering'* tab. In the VAST dataset we want to explore if the individuals are grouped based on the vaccine they received ('*Vaccine'* column selected under *'Additional Exploration Classes'*). We select *'Diagnosis'* and *'Vaccine'* as columns and 3 top features as rows. After setting up the desired parameters for the clustering analysis, we click *'Plot image'* button (A). The heatmap can be saved as a CSV file by clicking on the download button (B). We can also perform clustering analysis as described above in Use case 1.

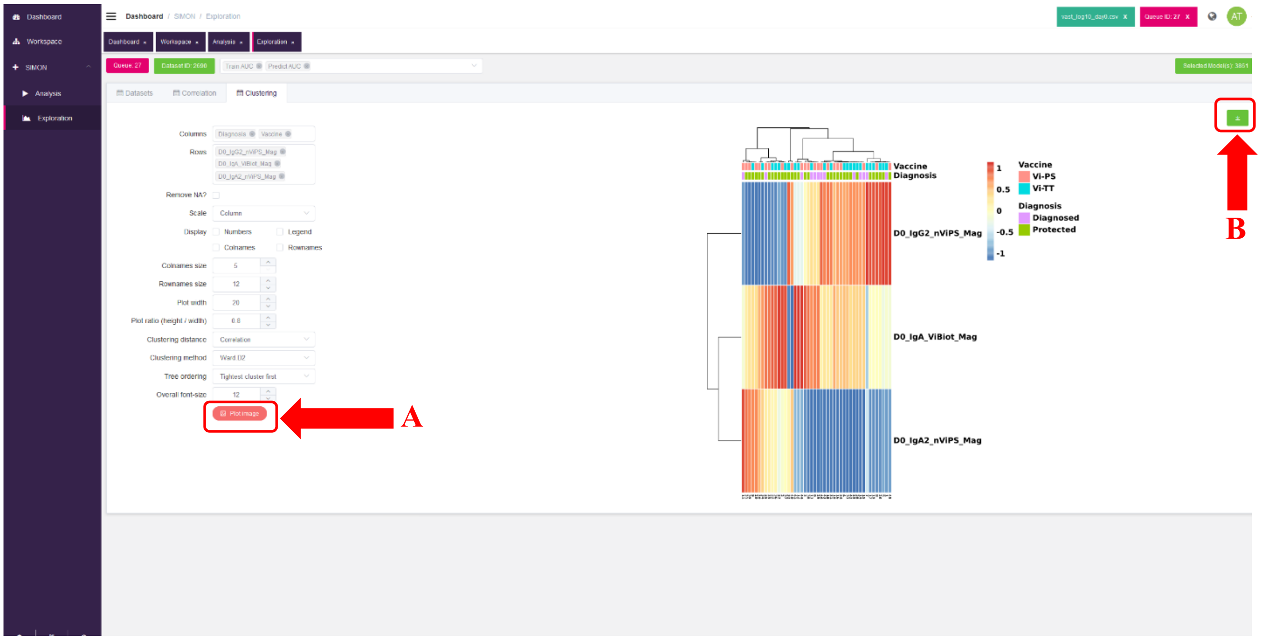

Use case 3. Identifying cellular immune signature associated with high-level of physical activity.

Steps 1-2 and 4-6 were performed as described above for the first two use cases.

Step 3. Setting-up analysis. For the Cyclists dataset, we used all columns as *'Predictors variables'* (A) and outcome column as the *'Response'*(B). Other parameters, training/test split and preprocessing were performed as shown in the screenshot below. Similar to the use case 2, we used multi-set intersection function for the initial dataset to find resamples (C). In total, 146 resamples were identified and analysis was performed using all resamples.

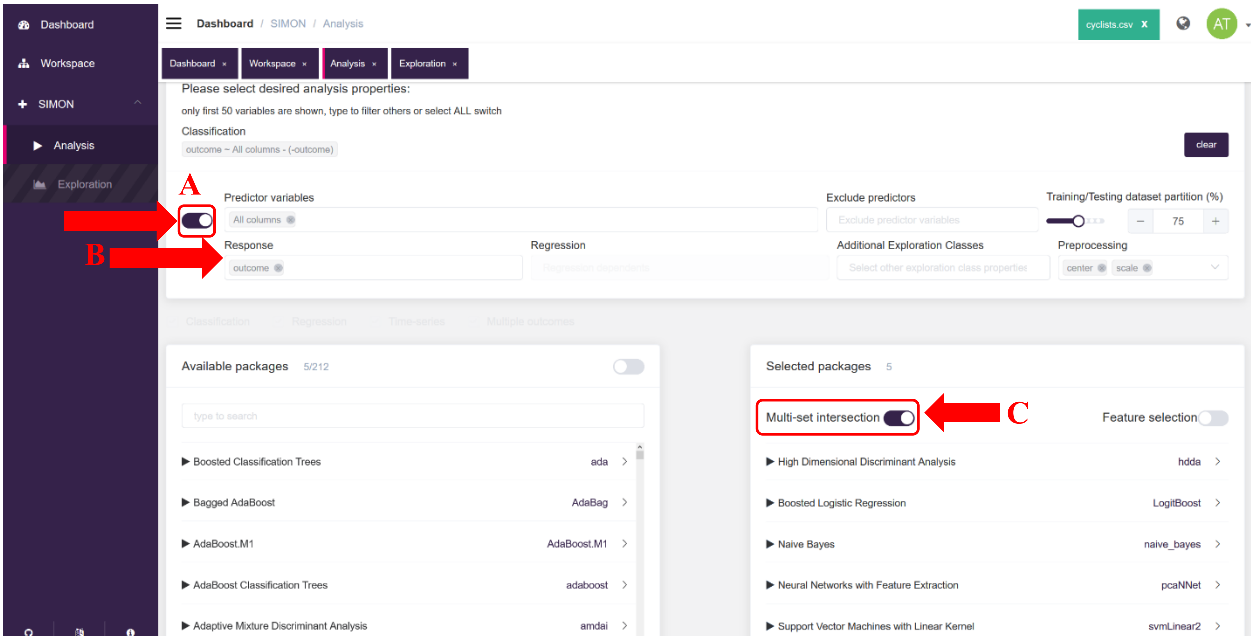

Use case 4. Building predictive model for the early-stage detection of colorectal cancer using microbiome.

Steps 1-2 and 4-6 were performed as described above.

Step 3. Setting-up analysis. After uploading and selecting the Zeller dataset, in the *'Analysis'* window, we selected all columns as *'Predictors variables'* (A) and we typed *'Study condition'* in the *'Response'* input form to find the outcome column (B). The initial dataset was divided 75% into training and 25% test set (C). For the preprocessing we applied '*center*', *'scale'* and remove near zero-variance ('*nzv*') (D). In total, five ML algorithms were selected (E) and analysis was started by pressing *'Validate data'* button.

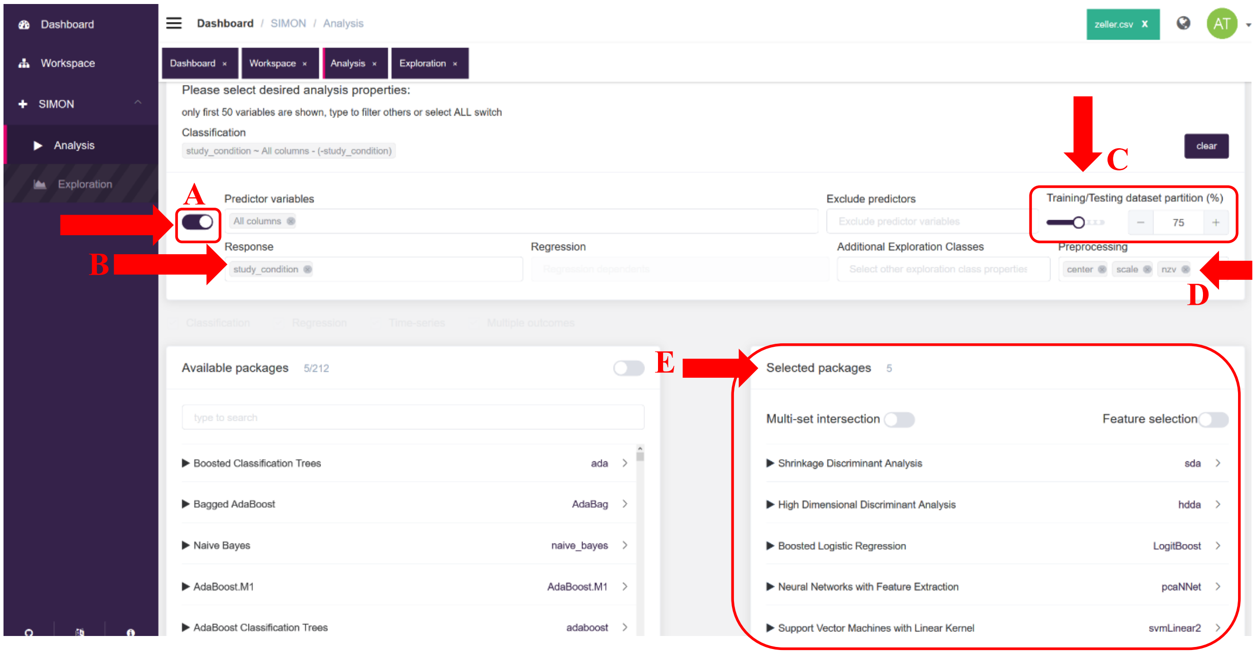

Use case 5. Building predictive model for detection of liver hepatocellular carcinoma cells using transcriptome data.

Steps 1-2 and 5-6 were performed as described above for the other Use cases.

Step 3. Setting-up analysis. For the LIHC dataset, analysis was started with selecting all columns as *'Predictors variables'* (A) and *'Group'* column (tumor or healthy cells) as the *'Response'* (B). The initial dataset was divided 75% into training and 25% test set (C). For the preprocessing we applied '*center*', *'scale'* and remove near zero-variance ('*nzv'*) (D). In total, five ML algorithms were selected (E) and analysis was started (*'Validate data'* button).

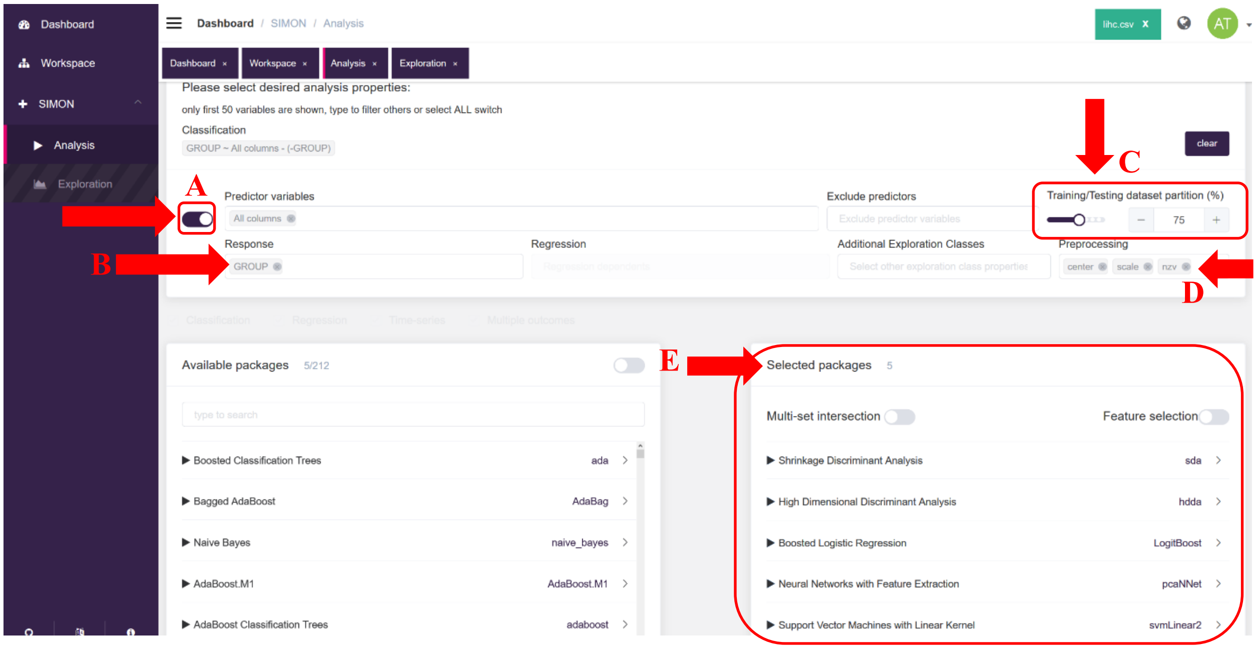

Step 4. Model evaluation and selection. The LIHC is the example of highly imballanced dataset, therefore in the *'Exploration'* window (tab becomes available upon selection of LIHC analysis row in the *'Dashboard'*) and select precision-recall AUC (train prAUC for the training set and prAUC for the test set) (A). The models are then ranked based on the train prAUC (B). The first model that has high train prAUC value, also performed well on the left-out test set. We save the generated model by clicking the download button (C). Visualization of model performance measurements is performed as described for other Use cases.

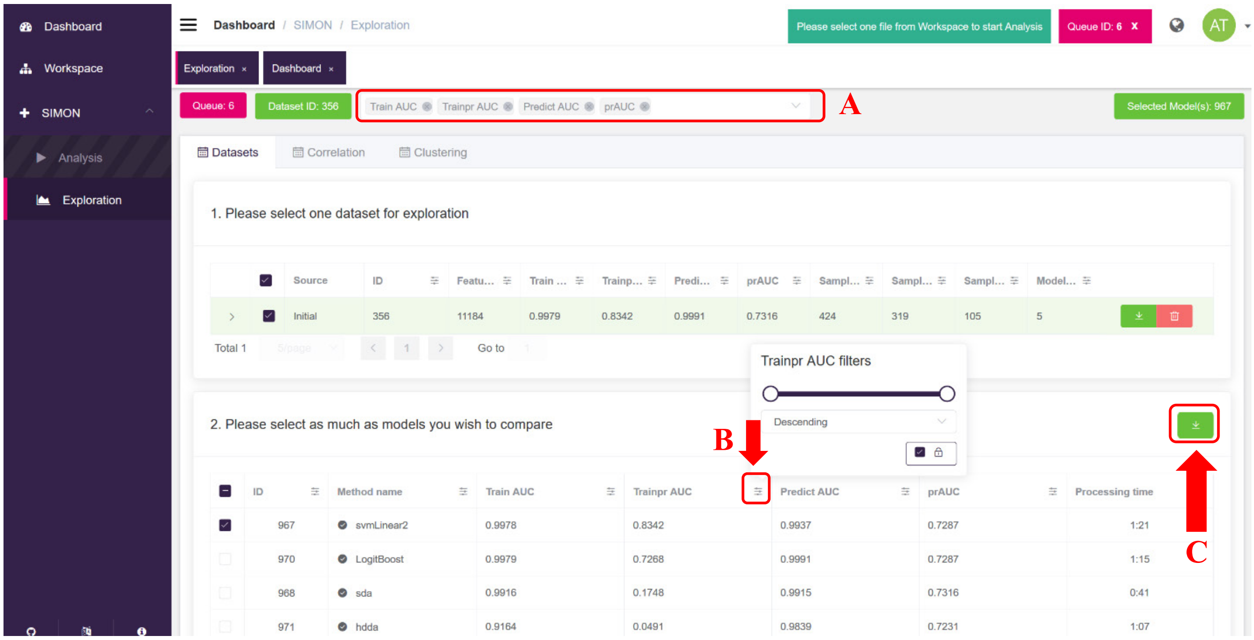

Supplemental figures

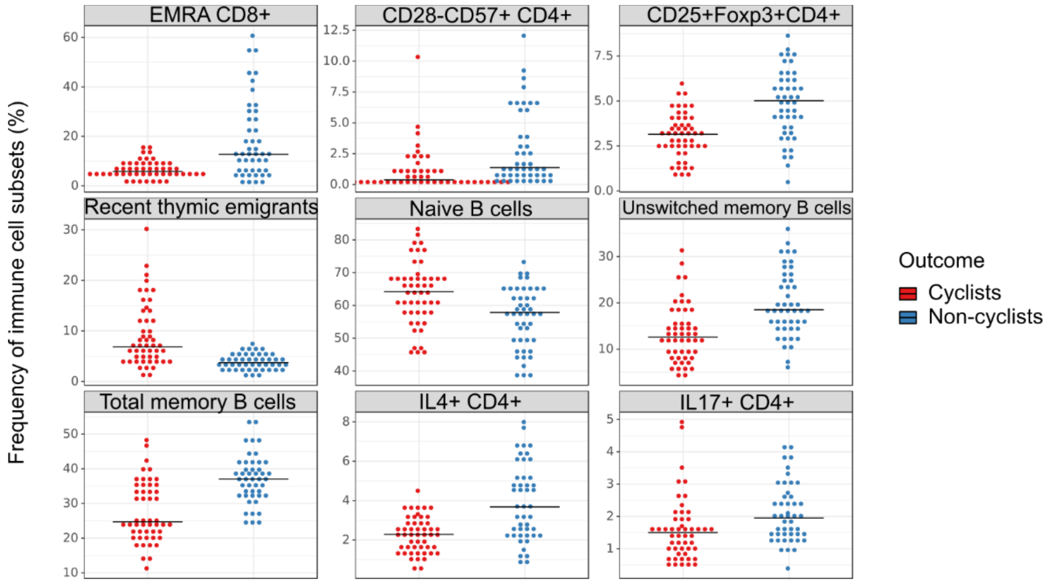

Figure S1. Frequency of immune cell subsets associated with high-level of physical activity. Dot plots represent distribution of immune cell subsets between cyclists (red dots) and non-cyclists (blue dots) as frequency (percentage of parent immune cell population) for the top nine selected features that contribute the most to the Cyclists model to discriminate between cyclists and non-cyclists. Each dot is one individual, lines indicate median values.

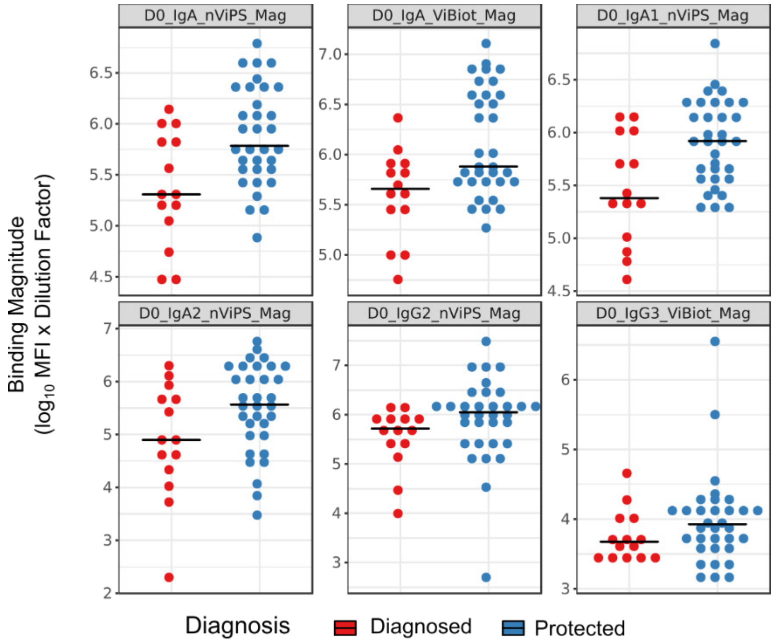

Figure S2. Antibody-mediated signature associated with the effective vaccine against *Salmonella* Typhi infection. Dot plots represent binding magnitude of indicated antibodies between diagnosed (red dots) and protected (blue dots) individuals. Each dot represents one individual, while lines indicate median values. The binding magnitude is log-transformed and given as Mean Fluorescence Intensity (MFI) multiplied by dilution factor. *D0*, day 0 (day of the challenge); *nViPS*, native Vi polysaccharide antigen; *ViBiot*, biotinylated Vi polysaccharide antigen; *Mag*, magnitude.

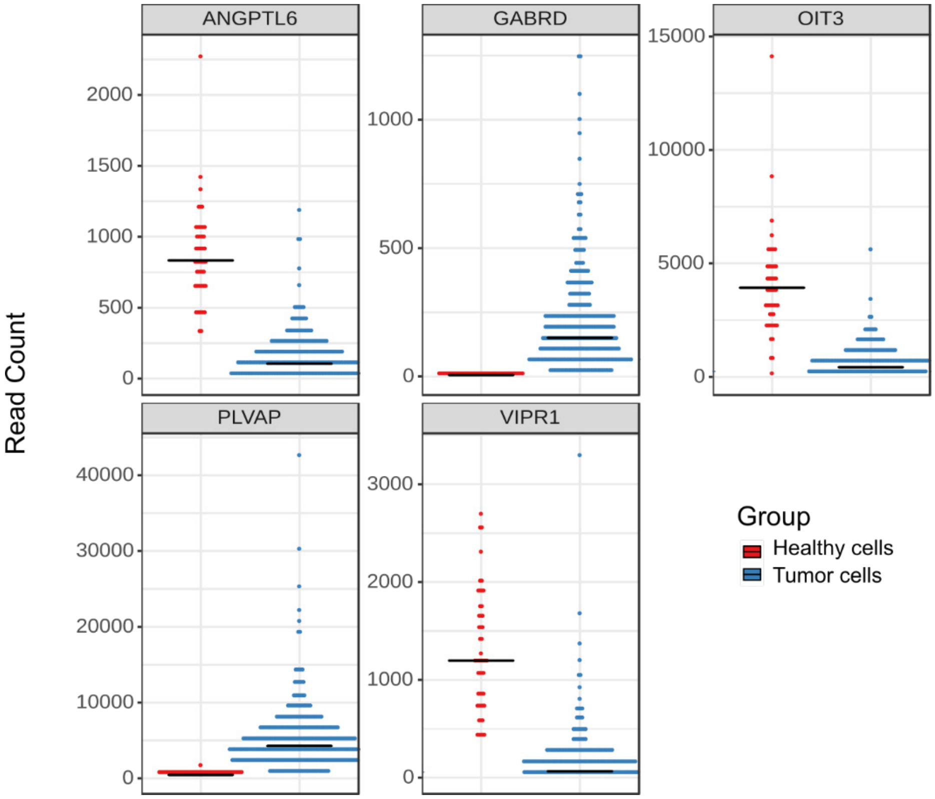

Figure S3. Gene expression signature specific for tumor cells. Dot plots represent read counts for top five genes that discriminate between healthy (red dots) and tumor (blue dots) cells selected in the top performing model. Each dot represents one sample, while lines indicate median values.

List of references of R packages used for Table S1:

1. Package: ada - Culp, M., Johnson, K. and Michailidis, G. (2016). ada: The R Package Ada for Stochastic Boosting. R package version 2.0-5. https://CRAN.R-project.org/package=ada
2. Package: plyr – Wickham, H. (2011). The Split-Apply-Combine Strategy for Data Analysis. Journal of Statistical Software 40(1), 1-29. URL http://www.jstatsoft.org/v40/i01/
3. Package: adabag – Alfaro, E., Gamez, M. and Garcia, N. (2013). adabag: An R Package for Classification with Boosting and Bagging. Journal of Statistical Software, 54(2), 1-35. URL http://www.jstatsoft.org/v54/i02/
4. Package: fastAdaboost – Chatterjee, S. (2016). fastAdaboost: a Fast Implementation of Adaboost. R package version 1.0.0. https://CRAN.R-project.org/package=fastAdaboost
5. Package: adaptDA – Bouveyron, C. (2014). adaptDA: Adaptive Mixture Discriminant Analysis. R package version 1.0. https://CRAN.R-project.org/package=adaptDA
6. Package: nnet - Venables, W. N. and Ripley, B. D. (2002) Modern Applied Statistics with S. Fourth Edition. Springer, New York. ISBN 0-387-95457-0
7. Package: bnclassify – Mihaljevic, B., Larranaga, P. and Bielza, C. (2018). bnclassify: Learning Bayesian Network Classifiers. The R Journal 10 (2): 455-468
8. Package: caret – Kuhn, M. (2020). caret: Classification and Regression Training. R package version 6.0-86. https://CRAN.R-project.org/package=caret
9. Package: earth – Milborrow, S. Derived from mda:mars by Trevor Hastie and Rob Tibshirani. Uses Alan Miller's Fortran utilities with Thomas Lumley's leaps wrapper. (2017). earth: Multivariate Adaptive Regression Splines. R package version 4.5.0. https://CRAN.R-project.org/package=earth
10. Package: mgcv - Wood, S.N. (2011) Fast stable restricted maximum likelihood \nand marginal likelihood estimation of semiparametric generalized linear \nmodels. Journal of the Royal Statistical Society (B) 73(1):3-36
11. Package: arm – Gelman, A. and Su, Y. (2018). arm: Data Analysis Using Regression and Multilevel/HierarchicalModels. R package version 1.10-1. https://CRAN.R-project.org/package=arm

12. Package: binda – Gibb, S. and Strimmer, K. (2015). binda: Multi-Class Discriminant Analysis using Binary Predictors. R package version 1.0.3. https://CRAN.R-project.org/package=binda
13. Package: party – Hothorn, T., Hornik, K.and Zeileis, A. (2006). Unbiased Recursive Partitioning: A Conditional Inference Framework. Journal of Computational and Graphical Statistics, 15(3), 651--674.
14. Package: partykit - Hothorn, T. and Zeileis, A. (2015). partykit: A Modular Toolkit for Recursive Partytioning in R. Journal of Machine Learning Research 16, 3905-3909. URL http://jmlr.org/papers/v16/hothorn15a.html
15. Package: bst – Wang, Z. (2019). bst: Gradient Boosting. R package version 0.3-17. https://CRAN.R-project.org/package=bst
16. Package: C50 – Kuhn, M. and Quinlan, R. (2020). C50: C5.0 Decision Trees and Rule-Based Models. R package version 0.1.3. https://CRAN.R-project.org/package=C50
17. Package: CHAID - The FoRt Student Project Team (2015). CHAID: CHi-squared Automated Interaction Detection R package version 0.1-2.
18. Package: rrcov – Todorov, V. and Filzmoser, P. (2009). An Object-Oriented Framework for Robust Multivariate Analysis. Journal of Statistical Software 32(3), 1-47. URL http://www.jstatsoft.org/v32/i03/
19. Package: rrcovHD – Todorov, V. (2019). rrcovHD: Robust Multivariate Methods for High Dimensional Data. R package version 0.2-6. https://CRAN.R-project.org/package=rrcovHD
20. Package: sparsediscrim – Ramey, J.A. (2017). sparsediscrim: Sparse and Regularized Discriminant Analysis. https://github.com/ramhiser/sparsediscrim; http://ramhiser.com
21. Package: deepboost – Marcous, D. and Sandbank, Y. (2017). deepboost: Deep Boosting Ensemble Modeling. R package version 0.1.6. https://CRAN.R-project.org/package=deepboost
22. Package: deepnet – Rong, X. (2014). deepnet: deep learning toolkit in R. R package version 0.2. https://CRAN.R-project.org/package=deepnet
23. Package: kerndwd - Wang, B. and Zou, H. (2018). kerndwd: Distance Weighted Discrimination (DWD) and Kernel Methods. R package version 2.0.2. https://CRAN.R-project.org/package=kerndwd

24. Package: kernlab - Karatzoglou, A., Smola, A., Hornik, K. and Zeileis, A. (2004). kernlab - An S4 Package for Kernel Methods in R. Journal of Statistical Software 11(9), 1-20. URL http://www.jstatsoft.org/v11/i09/
25. Package: earth – Milborrow, S.. Derived from mda:mars by Trevor Hastie and Rob Tibshirani. Uses Alan Miller's Fortran utilities with Thomas Lumley's leaps wrapper. (2017). earth: Multivariate Adaptive Regression Splines. R package version 4.5.0. https://CRAN.R-project.org/package=earth
26. Package: elmNN – Gosso, A. (2012). elmNN: Implementation of ELM (Extreme Learning Machine) algorithm for SLFN (Single Hidden Layer Feedforward Neural Networks). R package version 1.0. https://CRAN.R-project.org/package=elmNN
27. Package: evtree - Grubinger, T., Zeileis, A. and Pfeiffer, K. (2014). evtree: Evolutionary Learning of Globally Optimal Classification and Regression Trees in R. Journal of Statistical Software, 61(1), 1-29. URL http://www.jstatsoft.org/v61/i01
28. Package: caret – Kuhn, M. Contributions from Wing, J., Weston, S. Williams, A., Keefer, C., Engelhardt, A., Cooper, T., Mayer, Z., Kenkel, B., the R Core Team, Benesty, M., Lescarbeau, R., Ziem, A., Scrucca, L., Tang, Y., Candan, C. and Hunt, T. (2017). caret: Classification and Regression Training. R package version 6.0-76. https://CRAN.R-project.org/package=caret
29. Package: frbs - Riza, L.S., Bergmeir, C., Herrera, F. and Benitez, J.M. (2015). frbs: Fuzzy Rule-Based Systems for Classification and Regression in R. Journal of Statistical Software, 65(6), 1-30. URL http://www.jstatsoft.org/v65/i06/
30. Package: mgcv - Wood, S.N. (2011) Fast stable restricted maximum likelihood \nand marginal likelihood estimation of semiparametric generalized linear \nmodels. Journal of the Royal Statistical Society (B) 73(1):3-36
31. Package: mboost - Hothorn, T., Buehlmann, P., Kneib, T., Schmid, M. and Hofner, B.(2017). mboost: Model-Based Boosting, R package version 2.8-0, https://CRAN.R-project.org/package=mboost.
32. Package: gam – Hastie, T. (2017). gam: Generalized Additive Models. R package version 1.14-4. https://CRAN.R-project.org/package=gam
33. Package: h2o - LeDell, E., Gill, N., Aiello, S., Fu, A., Candel, A., Click, C., Kraljevic, T., Nykodym, T., Aboyoun, P., Kurka, M. and Malohlava, M. (2020). h2o: R Interface for the

'H2O' Scalable Machine Learning Platform. R package version 3.28.0.4. https://CRAN.R-project.org/package=h2o

34. Package: gbm - Greenwell, B., Boehmke, B., Cunningham, J. and GBM Developers (2019). gbm: Generalized Boosted Regression Models. R package version 2.1.5. https://CRAN.R-project.org/package=gbm
35. Package: glm2 – Marschner, I.C. (2011). Fitting generalized linear models with convergence problems. The R Journal, 3(2), 12-15. URL https://CRAN.R-project.org/package=glm2
36. Package: MASS - Venables, W. N. and Ripley, B. D. (2002) Modern Applied Statistics with S. Fourth Edition. Springer, New York. ISBN 0-387-95457-0
37. Package: gpls – Ding, B. (2018). gpls: Classification using generalized partial least squares. R package version 1.54.0.
38. Package: hda – Szepannek, G. (2016). hda: Heteroscedastic Discriminant Analysis. R package version 0.2-14. https://CRAN.R-project.org/package=hda
39. Package: HDclassif - Berge, L., Bouveyron, C. and Girard, S. (2012). HDclassif: An R Package for Model-Based Clustering and Discriminant Analysis of High-Dimensional Data. Journal of Statistical Software, 46(6), 1-29. URL http://www.jstatsoft.org/v46/i06/
40. Package: pls – Mevik, B., Wehrens, R. and Liland, K.H. (2019). pls: Partial Least Squares and Principal Component Regression. R package version 2.7-2. https://CRAN.R-project.org/package=pls
41. Package: kknn – Schliep, K. and Hechenbichler, K. (2016). kknn: Weighted k-Nearest Neighbors. R package version 1.3.1. https://CRAN.R-project.org/package=kknn
42. Package: klaR - Weihs, C., Ligges, U., Luebke, K. and Raabe, N. (2005). klaR Analyzing German Business Cycles. In Baier, D., Decker, R. and Schmidt-Thieme, L. (eds.). Data Analysis and Decision Support, 335-343, Springer-Verlag, Berlin.
43. Package: logicFS – Schwender, H. and Tietz, T. (2018). logicFS: Identification of SNP Interactions. R package version 2.2.0.
44. Package: caTools – Tuszynski, J. (2020). caTools: Tools: moving window statistics, GIF, Base64, ROC, AUC, etc. R package version 1.17.1.4. https://CRAN.R-project.org/package=caTools

45. Package: LogicReg – Kooperberg, C. and Ruczinski, I. (2019). LogicReg: Logic Regression. R package version 1.6.2. https://CRAN.R-project.org/package=LogicReg
46. Package: class - Venables, W. N. and Ripley, B. D. (2002) Modern Applied Statistics with S. Fourth Edition. Springer, New York. ISBN 0-387-95457-0
47. Package: mda - S original by Hastie, T. and Tibshirani, R. Original R port by Leisch, F., Hornik, K. and Ripley, B.D. (2017). mda: Mixture and Flexible Discriminant Analysis. R package version 0.4-10. https://CRAN.R-project.org/package=mda
48. Package: HiDimDA - Duarte Silva, A.P. (2015). HiDimDA: High Dimensional Discriminant Analysis. R package version 0.2-4. https://CRAN.R-project.org/package=HiDimDA
49. Package: RSNNS - Bergmeir, C. and Benitez, J.M. (2012). Neural Networks in R Using the Stuttgart Neural Network Simulator: RSNNS. Journal of Statistical Software, 46(7), 1-26. URL http://www.jstatsoft.org/v46/i07/
50. Package: keras – Allaire, J.J. and Chollet, F. (2019). keras: R Interface to 'Keras'. R package version 2.2.5.0. https://CRAN.R-project.org/package=keras
51. Package: FCNN4R – Klima, G. (2016), FCNN4R: Fast Compressed Neural Networks for R, R package version 0.6.2.
52. Package: monmlp – Cannon, A.J. (2017). monmlp: Monotone Multi-Layer Perceptron Neural Network. R package version 1.1.4. https://CRAN.R-project.org/package=monmlp
53. Package: nnet - Venables, W. N. and Ripley, B. D. (2002) Modern Applied Statistics with S. Fourth Edition. Springer, New York. ISBN 0-387-95457-0
54. Package: naivebayes – Majka, M. (2017). naivebayes: High Performance Implementation of the Naive Bayes Algorithm. R package version 0.9.1. https://CRAN.R-project.org/package=naivebayes
55. Package: nodeHarvest – Meinshausen, N. (2015). nodeHarvest: Node Harvest for Regression and Classification. R package version 0.7-3. https://CRAN.R-project.org/package=nodeHarvest
56. Package: ordinalNet - Wurm, M., Rathouz, P. and Hanlon, B. (2020). ordinalNet: Penalized Ordinal Regression. R package version 2.7. https://CRAN.R-project.org/package=ordinalNet

57. Package: e1071 - Meyer, D., Dimitriadou, E., Hornik, K., Weingessel, A. and Leisch, F. (2019). e1071: Misc Functions of the Department of Statistics Probability Theory Group (Formerly: E1071) TU Wien. R package version 1.7-3. https://CRAN.R-project.org/package=e1071
58. Package: ranger – Wright, M.N. and Ziegler, A. (2017). ranger: A Fast Implementation of Random Forests for High Dimensional Data in C++ and R. Journal of Statistical Software, 77(1), 1-17. doi:10.18637/jss.v077.i01
59. Package: dplyr - Wickham, H., François, R., Henry, L. and Müller, K. (2020). dplyr: A Grammar of Data Manipulation. R package version 0.8.5. https://CRAN.R-project.org/package=dplyr
60. Package: obliqueRF – Menze, B. and Splitthoff, N. (2012). obliqueRF: Oblique Random Forests from Recursive Linear Model Splits. R package version 0.3. https://CRAN.R-project.org/package=obliqueRF
61. Package: snn - Sun, W., Qiao, X. and Cheng, G. (2015). snn: Stabilized Nearest Neighbor Classifier. R package version 1.1. https://CRAN.R-project.org/package=snn
62. Package: pamr - Hastie, T., Tibshirani, R., Narasimhan, B. and Chu, G. (2019). pamr: Pam: Prediction Analysis for Microarrays. R package version 1.56.1. https://CRAN.R-project.org/package=pamr
63. Package: randomForest – Liaw, A. and Wiener, M. (2002). Classification and Regression by randomForest. R News 2(3), 18-22.
64. Package: foreach - Microsoft and Weston, S. (2020). foreach: Provides Foreach Looping Construct. R package version 1.5.0. https://CRAN.R-project.org/package=foreach
65. Package: partDSA – Molinaro, A.M., Lostritto, K. and van der Laan, M.J. (2010). partDSA: Deletion/Substitution/Addition Algorithm for Partitioning the Covariate Space in Prediction. Bioinformatics, 26(10), 1357-63.
66. Package: penalizedLDA – Witten, D. (2015). penalizedLDA: Penalized Classification using Fisher's Linear Discriminant. R package version 1.1. https://CRAN.R-project.org/package=penalizedLDA
67. Package: stepPlr – Park, M.Y. and Hastie, T. (2018). stepPlr: L2 Penalized Logistic Regression with Stepwise VariableSelection. R package version 0.93. https://CRAN.R-project.org/package=stepPlr

68. Package: proxy – Meyer, D. and Buchta, C. (2019). proxy: Distance and Similarity Measures. R package version 0.4-23. https://CRAN.R-project.org/package=proxy
69. Package: protoclass – Bien, J. and Tibshirani, R. (2013). protoclass: Interpretable classification with prototypes. R package version 1.0. https://CRAN.R-project.org/package=protoclass
70. Package: randomGLM – Song, L. and Langfelder, P. (2013). randomGLM: Random General Linear Model Prediction. R package version 1.02-1. https://CRAN.R-project.org/package=randomGLM
71. Package: rBorist – Seligman, M. (2019). Rborist: Extensible Parallelizable Implementation of the Random Forest Algorithm. R package version 0.2-3. https://CRAN.R-project.org/package=Rborist
72. Package: LiblineaR – Helleputte, T. (2017). LiblineaR: Linear Predictive Models Based On The Liblinear C/C++ Library.R package version 2.10-8.
73. Package: rFerns – Kursa, M.B. (2014). rFerns: An Implementation of the Random Ferns Method for General-Purpose Machine Learning. Journal of Statistical Software 61(10) 1-13. URL http://www.jstatsoft.org/v61/i10/
74. Package: robustDA – Bouveyron, C. and Girard, S. (2015). robustDA: Robust Mixture Discriminant Analysis. R package version 1.1. https://CRAN.R-project.org/package=robustDA
75. Package: rocc – Lauss, M. (2019). rocc: ROC Based Classification. R package version 1.3. https://CRAN.R-project.org/package=rocc
76. Package: rotationForest – Ballings, M. and Van den Poel, D. (2017). rotationForest: Fit and Deploy Rotation Forest Models. R package version 0.1.3. https://CRAN.R-project.org/package=rotationForest
77. Package: rpart – Therneau, T., Atkinson, B. and Ripley, B. (2017). rpart: Recursive Partitioning and Regression Trees. R package version 4.1-11. https://CRAN.R-project.org/package=rpart
78. Package: rpartScore - Galimberti, G., Soffritti, G. and Di Maso, M. (2012). Classification Trees for Ordinal Responses in R: The rpartScore Package. Journal of Statistical Software, 47(10), 1-25. URL http://www.jstatsoft.org/v47/i10/

79. Package: RRF – Deng, H. (2013). Guided Random Forest in the RRF Package. arXiv:1306.0237.

80. Package: sda - Ahdesmaki, M., Zuber, V., Gibb, S. and Strimmer, K. (2015). sda: Shrinkage Discriminant Analysis and CAT Score Variable Selection. R package version 1.3.7. https://CRAN.R-project.org/package=sda

81. Package: sdwd – Wang, B. and Zou, H. (2020). sdwd: Sparse Distance Weighted Discrimination. R package version 1.0.3. https://CRAN.R-project.org/package=sdwd

82. Package: ipred – Peters, A. and Hothorn, T. (2019). ipred: Improved Predictors. R package version 0.9-9. https://CRAN.R-project.org/package=ipred

83. Package: sparseLDA – Clemmensen, L. and contributions by Kuhn, M. (2016). sparseLDA: Sparse Discriminant Analysis. R package version 0.1-9. https://CRAN.R-project.org/package=sparseLDA

84. Package: spls - Chung, D., Chun, H. and Keles, S. (2019). spls: Sparse Partial Least Squares (SPLS) Regression and Classification. R package version 2.2-3. https://CRAN.R-project.org/package=spls

85. Package: vbmp – Lama, N. and Girolami, M. (2018). vbmp: Variational Bayesian Multinomial Probit Regression. R package version 1.50.0. http://bioinformatics.oxfordjournals.org/cgi/content/short/btm535v1

86. Package: VGAM – Yee, T.W. (2015). Vector Generalized Linear and Additive Models: With an Implementation in R. New York, USA: Springer.

87. Package: wsrf - Zhao, H., Williams, G.J. and Huang, J.Z. (2017). wsrf: An R Package for Classification with Scalable Weighted Subspace Random Forests. Journal of Statistical Software, 77(3), 1-30. doi:10.18637/jss.v077.i03

88. Package: xgboost - Chen, T., He, T., Benesty, M., Khotilovich, V., Tang, Y., Cho, H., Chen, K., Mitchell, R., Cano, I., Zhou, T., Li, M., Xie, J., Lin, M., Geng, Y. and Li, Y. (2020). xgboost: Extreme Gradient Boosting. R package version 1.0.0.2. https://CRAN.R-project.org/package=xgboost
